# Genetic Evidence That the Non-Homologous End-Joining Repair Pathway Is Involved in LINE Retrotransposition

**DOI:** 10.1371/journal.pgen.1000461

**Published:** 2009-04-24

**Authors:** Jun Suzuki, Katsumi Yamaguchi, Masaki Kajikawa, Kenji Ichiyanagi, Noritaka Adachi, Hideki Koyama, Shunichi Takeda, Norihiro Okada

**Affiliations:** 1 Graduate School of Bioscience and Biotechnology, Tokyo Institute of Technology, Midori-ku, Yokohama, Kanagawa, Japan; 2 Division of Human Genetics, Department of Integrated Genetics, National Institute of Genetics, Research Organization of Information and Systems, Mishima, Shizuoka, Japan; 3 International Graduate School of Arts and Sciences, Yokohama City University, Kanazawa-ku, Yokohama, Kanagawa, Japan; 4 Radiation Genetics, Graduate School of Medicine, Kyoto University, Konoe Yoshida, Kyoto, Kyoto, Japan; Fred Hutchinson Cancer Research Center, United States of America

## Abstract

Long interspersed elements (LINEs) are transposable elements that proliferate within eukaryotic genomes, having a large impact on eukaryotic genome evolution. LINEs mobilize via a process called retrotransposition. Although the role of the LINE-encoded protein(s) in retrotransposition has been extensively investigated, the participation of host-encoded factors in retrotransposition remains unclear. To address this issue, we examined retrotransposition frequencies of two structurally different LINEs—zebrafish ZfL2-2 and human L1—in knockout chicken DT40 cell lines deficient in genes involved in the non-homologous end-joining (NHEJ) repair of DNA and in human HeLa cells treated with a drug that inhibits NHEJ. Deficiencies of NHEJ proteins decreased retrotransposition frequencies of both LINEs in these cells, suggesting that NHEJ is involved in LINE retrotransposition. More precise characterization of ZfL2-2 insertions in DT40 cells permitted us to consider the possibility of dual roles for NHEJ in LINE retrotransposition, namely to ensure efficient integration of LINEs and to restrict their full-length formation.

## Author Summary

Long interspersed elements (LINEs) are transposable elements that mobilize and amplify their own copies within eukaryotic genomes. Although LINEs had been considered as “junk” DNA, recent studies have suggested that the LINE-induced alterations of host chromosomes are a major driving force for eukaryotic genome evolution. LINEs mobilize via a mechanism called retrotransposition, in which transcribed LINE RNA is reverse transcribed into DNA that is then integrated into the host chromosome. Although the role of LINE-encoded proteins in retrotransposition has been revealed, the participation of host-encoded proteins has not been well investigated. Here, using knockout chicken DT40 cell lines, we present genetic evidence that the host-encoded proteins involved in repair of DNA double-strand breaks participate in LINE retrotransposition. More precise characterization of LINE insertions in DT40 cells suggested dual roles for these host DNA repair proteins in LINE retrotransposition; one function is required for efficient integration of LINEs and the other restricts their full-length formation.

## Introduction

Long interspersed elements (LINEs) and short interspersed elements (SINEs) are transposable elements widely distributed in eukaryotic genomes [1],[2]; as such, they substantially affect genome complexity and evolution [3],[4]. These elements mobilize and amplify their own sequences by a mechanism called retrotransposition. LINEs are 4–7 kbp in length and typically encode two open reading frames (ORFs), ORF1 and ORF2, both of which are essential for LINE retrotransposition [5],[6]. During retrotransposition, LINEs are first transcribed into messenger RNA (mRNA) from which the LINE-encoded proteins are translated (Figure S1A). Next, the LINE mRNA and proteins form a complex [7],[8] and move to target sites on a host chromosome where the LINE-encoded endonuclease (EN) nicks a strand on the DNA duplex. The LINE-encoded reverse transcriptase (RT) then reverse transcribes the LINE mRNA using the 3′ hydroxyl group generated by the nick as a primer; this reaction is called target-primed reverse transcription [5],[9],[10]. Thereafter, the newly synthesized LINE is integrated into the host chromosome, at which time sequence alterations are generated at the target site. The position of the second strand cleavage is considered to define which kind of target site alteration is generated (Figure S1B) [11]. In the model, second-strand cleavage downstream of the initial first-strand nick generates target site duplication (TSD), cleavage at the same site generates blunt end joining (BEJ), and cleavage upstream generates target site truncation (TST). However, the precise mechanism of the integration remains unclear (Figure S1A). A DNA double-strand break (DSB) would necessarily need to be generated at the target site to integrate the newly synthesized LINE element. In fact, overexpression of human LINE L1 in mammalian cultured cells induces DSBs in the host chromosomal DNA [12]. Accumulating evidence has revealed that several host-encoded DNA repair proteins are involved in the mobility reactions of retrotransposons, such as yeast LTR retrotransposons and bacterial group II introns [for review, 13]. However, the roles of host factors in LINE retrotransposition remain unclear. Only a few genetic studies have identified host proteins that are involved in LINE retrotransposition: the ataxia-telangiectasia mutated (ATM) protein—a protein kinase involved in cellular responses to DSBs—is suggested to participate in L1 retrotransposition [12], and the ERCC1/XPF endonuclease—which functions in nucleotide excision repair—is involved in limiting L1 retrotransposition [14]. It is conceivable that the LINE retrotransposition reactions involve other host factors, such as proteins of the non-homologous end-joining (NHEJ) pathway, that predominate in DSB repair in vertebrate cells [15].

The core components involved in vertebrate NHEJ are the Ku70 and Ku80 heterodimer (Ku70/80), the catalytic subunit of DNA protein kinase (DNA-PKcs), DNA ligase IV (LigIV), and Xrcc4. Initially, Ku70/80 binds to the broken DNA ends. DNA-PKcs is recruited to the ends by Ku70/80, with which it maintains the broken ends in proximity and provides a platform for the recruitment of other enzymes [16]. The kinase activity of DNA-PKcs—which is activated upon recruitment to the broken ends—is considered to enhance the DSB signal via phosphorylation of many downstream targets, although physiological targets of the phosphorylation remain obscure [for example, 17]. LigIV, which forms a tight complex with Xrcc4, is responsible for ligation of the broken DNA ends [18],[19]. There are other proteins implicated in NHEJ. During NHEJ, a pair of broken ends that are incompatible for ligation is processed into compatible ends by a nuclease(s), such as Artemis, and/or a polymerase(s), although their significance in NHEJ is less clear. Interestingly, broken ends are still repaired by NHEJ in cells deficient in the core NHEJ components such as Ku proteins or LigIV, suggesting that NHEJ can be achieved by at least two distinct pathways [20],[21]. To distinguish these two processes, the NHEJ pathway that depends on the core components is denoted ‘classical’, and the other pathway, which can occur without the core components, is called ‘alternative’. In contrast to the classical NHEJ, the enzymes responsible for the alternative NHEJ remain uncharacterized.

Recently, we established a new system to detect LINE retrotransposition in the chicken B lymphocyte cell line, DT40, using two different kinds of LINEs, zebrafish ZfL2-2 and human L1 [22] (Figure S2). Here, we applied this system to Ku70^−/−^, Artemis^−/−^, and LigIV^−/−^ DT40 cell lines to determine the effect of these knockouts on the retrotransposition frequencies (RFs) of ZfL2-2 and L1. We then characterized ZfL2-2 insertions retrotransposed in the chromosomal DNA of DT40 cells to obtain evidence for the involvement of NHEJ factors in the LINE integration reaction. In addition, we examined the possible involvement of DNA-PKcs in LINE integration in human HeLa cells using NU7026, an inhibitor of DNA-PKcs activity.

## Results

### Disruption of Genes Involved in NHEJ Decreases the RF of Zebrafish ZfL2-2 and Human L1 LINEs in Chicken DT40 Cells

**Figure 1:**
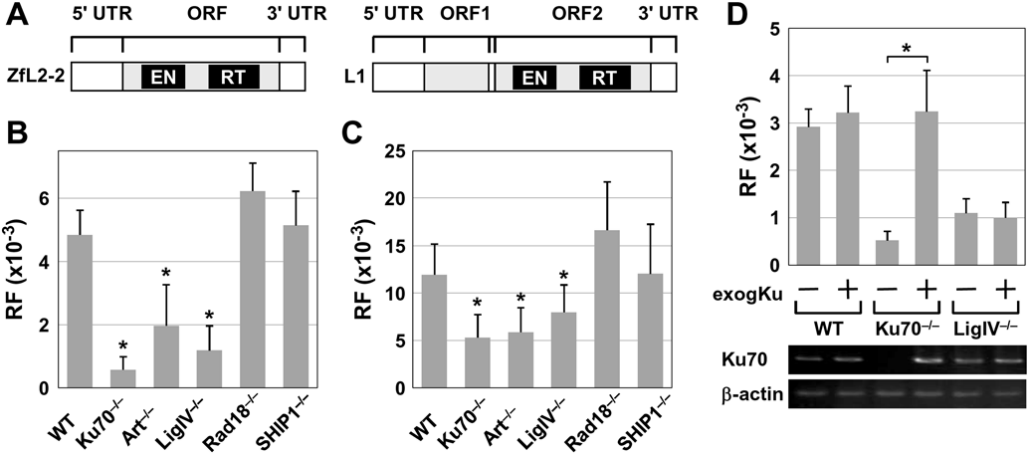
Retrotransposition frequencies (RFs) in DT40 cell lines. (A) Schematic of zebrafish ZfL2-2 and human L1. ZfL2-2 encodes only one ORF, but L1 encodes two ORFs. (B) RFs of ZfL2-2 in six different DT40 cell lines. (C) RFs of L1 in six different DT40 cell lines. (B, C) Means and standard deviations of RFs. WT, wild-type DT40 cell line. Ku70^−/−^, Ku70-deficient DT40 cell line. Art^−/−^, Artemis-deficient DT40 cell line. LigIV^−/−^, DNA ligase IV-deficient DT40 cell line. Rad18^−/−^, Rad18-deficient DT40 cell line. SHIP1^−/−^, hematopoietic-restricted SH2-containing inositol 5′-phosphatase-1-deficient DT40 cell line. An asterisk indicates *P*<0.01 by two-tailed Student's t-test. For ZfL2-2: WT vs Ku70^−/−^, *P* = 1.7×10^−8^; WT vs Art^−/−^, *P* = 2.8×10^−4^; WT vs LigIV^−/−^, *P* = 1.2×10^−6^. For L1: WT vs Ku70^−/−^, *P* = 1.1×10^−4^; WT vs Art^−/−^, *P* = 6.6×10^−5^; WT vs LigIV^−/−^, *P* = 5.7×10^−3^. (D) Ku70 complementation assay. The control expression vector, pAneo, or the Ku70 expression vector, chicken Ku70/pAneo, was transiently transfected into the WT, Ku70^−/−^, and LigIV^−/−^ DT40 cell lines (exogKu − or +, respectively). Using these transiently transfected cell lines, the ZfL2-2 retrotransposition assay was performed. Mean values (with standard deviations) of the ZfL2-2 RFs are shown. Transcription of the exogenous (cloned) and/or endogenous Ku70 gene was detected by RT-PCR (middle). Transcription of the β-actin gene was also detected by RT-PCR as a control (bottom). The asterisk indicates *P*<1×10^−4^ by two-tailed Student's t-test (for Ku70^−/−^ cells, *P* = 4.7×10^−5^).

To investigate whether host factors participating in NHEJ are involved in LINE retrotransposition, we examined RFs of two types of LINEs that have different structural characteristics—zebrafish ZfL2-2 and human L1—using wild-type (WT) and five knockout DT40 cell lines. The knockout DT40 cell lines were deficient in the genes encoding Ku70, Artemis, LigIV, SHIP1, or Rad18; the first three cell lines are related to NHEJ, and the others are not (Figure 1, Tables S1, S2). Because the intrinsic colony-forming capacities varied among these cell lines, we compensated for this aspect by including the plating efficiency in the RF calculation (see Materials and Methods). The RF of ZfL2-2 decreased by about 2- to 8-fold relative to the WT DT40 in all NHEJ-deficient cell lines examined here (Figure 1B; Ku70^−/−^, Art^−/−^ and LigIV^−/−^). On the other hand, knockout of the Rad18 or SHIP1 gene, neither of which is related to the NHEJ pathway, did not affect the RF (Figure 1B; Rad18^−/−^and SHIP1^−/−^). These results suggest that the NHEJ pathway plays a role in ZfL2-2 retrotransposition in these chicken cells. Similar retrotransposition results were obtained using L1, although the decrease in the L1 RF in Ku70^−/−^ and LigIV^−/−^ was smaller than that for ZfL2-2 (Figure 1C; see also Table S1, S2).

### Expression of Cloned Chicken Ku70 Rescues the RF Decrease of ZfL2-2 in Ku70^−/−^ Cells

To confirm that the RF decrease in the Ku70-defective cells was caused by Ku70 disruption, ZfL2-2 retrotransposition was assessed in three DT40 cell lines, WT, Ku70^−/−^ and LigIV^−/−^, with transient expression of a cloned chicken Ku70 gene (Figure 1D, Table S3). Transcription of the cloned and/or endogenous Ku70 genes in each sample was verified by RT-PCR. Expression of the cloned Ku70 in WT and LigIV^−/−^ cells did not significantly alter the ZfL2-2 RF. In contrast, exogenous Ku70 expression in Ku70^−/−^ cells dramatically increased the ZfL2-2 RF to a level comparable to WT cells. These results indicate that the decrease of ZfL2-2 RF in Ku70^−/−^ cells was indeed caused by Ku70 disruption.

### The EN Activity of ZfL2-2 and L1 Does Not Influence Viability of WT and NHEJ-Defective Cell Lines

**Figure 2:**
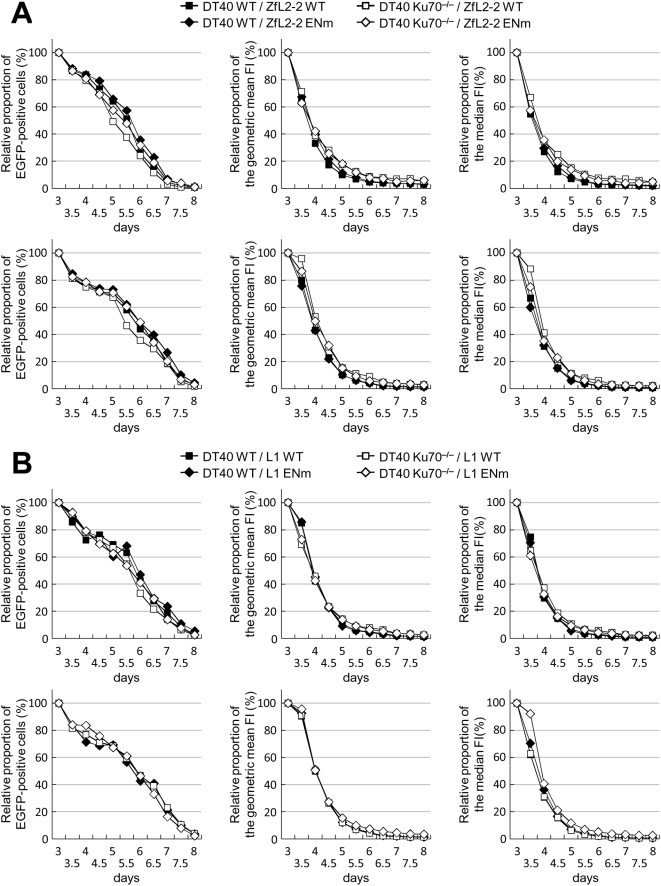
Effect of LINE expression on DT40 cell viability. DT40 cells were co-transfected with pEGFP-FLAG-1 and one of the LINE expression vectors (pBZ2-5, p131.11, pJM102/L1.3, or pJM102/L1.3 H230A) by electroporation (see *Tracing of EGFP-positive cells* in the Materials and Methods section). After transfection, the cells were monitored for 8 days. (A) ZfL2-2 expression in DT40 cells. The relative proportion of EGFP-expressing cells (left), the geometric mean of the EGFP fluorescence intensity (FI) (middle) and the median of the EGFP FI (right) calculated using the values 3 days after electroporation as the standard are indicated (the raw data are shown in Figures S5 and S6). DT40 WT, wild-type DT40 cell line. DT40 Ku70^−/−^, Ku70-deficient DT40 cell line. ZfL2-2 WT, wild-type ZfL2-2 element. ZfL2-2 ENm, endonuclease-mutated ZfL2-2 element. Two independent experiments were performed (upper and lower panels). (B) L1 expression in DT40 cells. The relative proportion of EGFP-expressing cells (left), the geometric mean of the EGFP FI (middle) and the median of the EGFP FI (right) calculated using the values 3 days after electroporation as the standard are indicated (the raw data are shown in Figures S9 and S10). DT40 WT, wild-type DT40 cell line. DT40 Ku70^−/−^, Ku70-deficient DT40 cell line. L1 WT, wild-type L1 element. L1 ENm, endonuclease-mutated L1 elements. Two independent experiments were performed (upper and lower panels).

The NHEJ-defective DT40 cell lines are sensitive to intense ionizing radiation [23],[24], indicating that the cells cannot efficiently repair radiation-induced DSBs, causing cell death. If the expression of ZfL2-2 or L1 in DT40 cells induces DSBs in chromosomal DNA as in the case of the L1 expression in HeLa cells [12], the NHEJ-defective DT40 cells may be more sensitive to such LINE-induced DSBs than WT cells. If this is the case, it is possible that the decrease of ZfL2-2 and L1 RF observed in the NHEJ-defective DT40 cells only reflects cell death caused by the LINE-induced DSBs, which cannot be compensated for by the plating efficiency in our assay (see Figure S3). To examine this possibility, we monitored the viability of WT and mutant DT40 cells transfected with the LINE expression vector. As shown in Figure S4, when two different fluorescence protein expression vectors (enhanced green fluorescence protein (EGFP) and DsRed-Express) were mixed and co-electroporated into DT40 cells, most transfected (fluorescence-positive) cells (>80%) express both of the two fluorescent proteins, and the amounts of proteins expressed from the co-transfected vectors were roughly proportional to each other (Figure S4D and S4E). Hence, to trace the LINE-expressing cells, an EGFP expression vector was electroporated together with the LINE expression vector into DT40 cells, and the EGFP expression and its intensity were monitored as shown in Figures S5, S6, S7, S8, S9, S10, S11, and S12. This EGFP monitoring was conducted from 3 to 8 days after electroporation, during which cell division occurred at least eight times (data not shown). EGFP expression observed on the eighth day was minimal, showing the detection limit. As shown in Figure 2, the relative ratio of the amount of EGFP-expressing cells at each time point to that of the third day was similar between WT and Ku70^−/−^ DT40 cells up to the end of the monitoring. In addition, the time course of the relative ratio of the geometric mean and the median of the EGFP intensity was similar between the WT and Ku70^−/−^ DT40 cells (Figure 2). Moreover, the time course of the values did not change when a point mutation that abolishes LINE EN activity was introduced in the ZfL2-2 and L1 elements. Similar results were obtained from Artemis^−/−^ and LigIV^−/−^ cell lines (Figure S13, S14). These results indicate that LINE EN expression does not influence the viability of WT and NHEJ-defective cell lines. Thus, the decrease in LINE RF in the NHEJ-defective cell lines is likely to be related to the involvement of NHEJ in LINE retrotransposition in DT40 cells.

### Retrotransposition of ZfL2-2 and L1 in DT40 Cells Depends on Their Own ENs

LINE retrotransposition typically depends on the LINE's own EN [5],[25],[26]. In contrast, a previous study reported that a fraction of human L1 retrotransposition in Chinese hamster ovary (CHO) cells was not dependent on L1 EN [27],[28]. This L1 EN-independent retrotransposition was enhanced in mutant CHO cells defective in a gene involved in NHEJ [27]. This report prompted us to consider that ZfL2-2 and L1 might atypically retrotranspose in NHEJ-defective DT40 cells through an EN-independent manner. To examine this possibility, we examined RFs of the EN-defective ZfL2-2 and L1 elements in the NHEJ-defective DT40 cell lines (Table S1, S2). In both NHEJ-defective and WT cell lines, no G418-resistant colonies were formed with these EN mutants, indicating that mobilization of ZfL2-2 and L1 in the DT40 cell lines examined here is dependent on their own ENs. Although we have not resolved the reason why the dependence of LINE retrotransposition on EN differs in chicken and hamster cells, this may reflect the differences in DNA repair that exist between these cells as discussed by Morrish *et al.* [27].

### NHEJ Defect–Dependent Structural Alterations of ZfL2-2 Insertions in Chicken DT40 Cells

**Figure 3:**
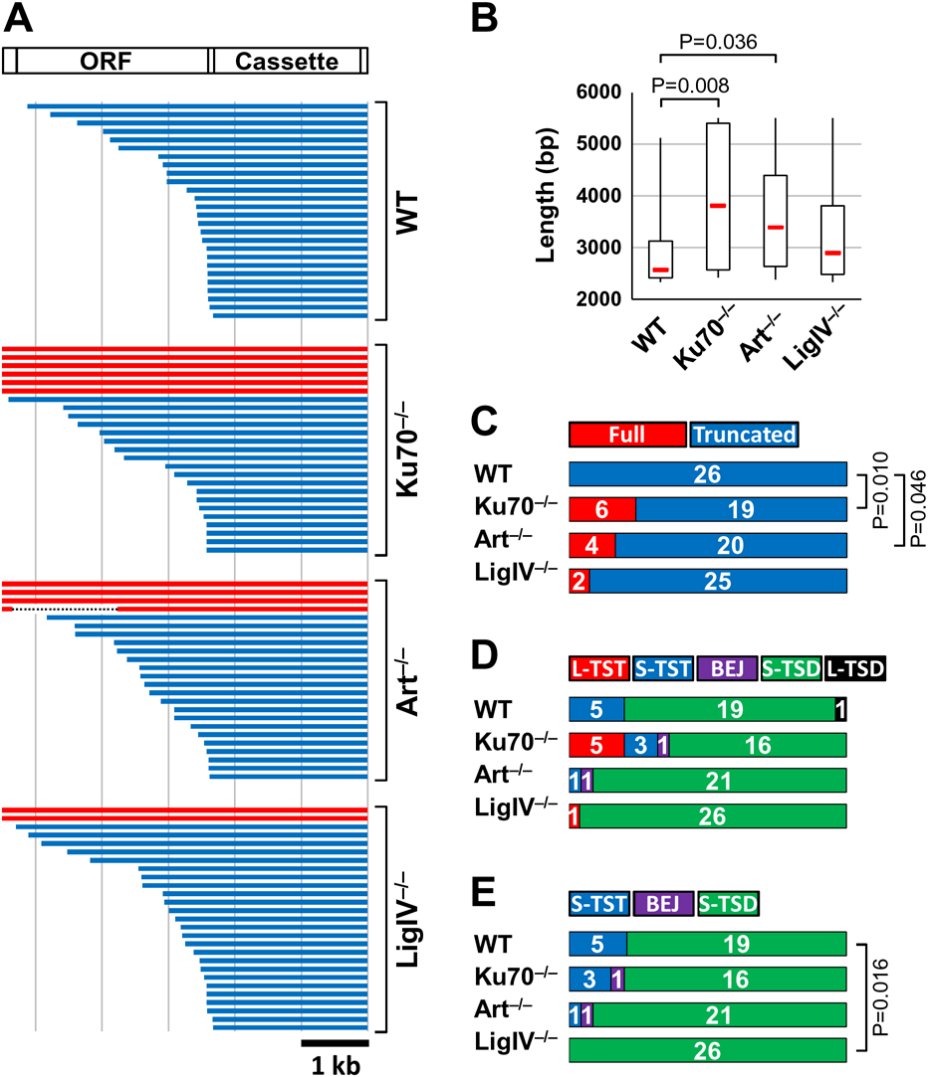
Characterization of ZfL2-2 insertions in DT40 cells. Abbreviations are as defined for Figure 1. (A) ZfL2-2 insertions isolated from DT40 cells. The top diagram shows the full-length ZfL2-2 element containing the *mneoI_400_/ColE1* cassette (Cassette) in the 3′ UTR. ORF, open reading frame. Each horizontal line represents one of the 26, 25, 24 or 27 ZfL2-2 insertions isolated from the various DT40 cell lines. Blue lines represent insertions with a 5′ truncation. Red lines represent full-length insertions. The dashed line in Art^−/−^ indicates a deletion. (B) A box-and-whisker plot shows the median (red line), the first and third quartiles, and the upper and lower limits of the length of insertions indicated in (A). *P* values less than 0.05 are indicated (Mann-Whitney U test). (C) Full-length vs. truncated elements. The ZfL2-2 insertions in (A) were categorized by the absence (Full) or presence (Truncated) of a 5′ truncation. The number of insertions identified is indicated inside each bar. *P* values less than 0.05 are indicated (two-sided Fisher's exact test). (D) Target site alterations. The ZfL2-2 insertions shown in (A) were categorized with regard to target site alterations. The number of insertions identified is indicated inside each bar. L-TST, long target site truncation (>20 bp). S-TST, short target site truncation (≤20 bp). BEJ, blunt end joining. S-TSD, short target site duplication (≤20 bp). L-TSD, long target site duplication (>20 bp). (E) Short target site alterations. The ZfL2-2 insertions with short target site alterations in (D) were compared. The number of insertions identified is indicated inside each bar. Abbreviations and definitions are as for panel D. *P* values less than 0.05 are indicated (Wilcoxon Rank Sum test).

To determine in which step of the retrotransposition reaction each NHEJ factor is involved, we determined and analyzed the 5′ and 3′ junction sequences of 102 ZfL2-2 inserts in chromosomal DNA of WT, Ku70^−/−^, Artemis^−/−^ and LigIV^−/−^ DT40 cells (26, 25, 24 and 27 insertions, respectively; Table S4). We previously showed that ∼40% of ZfL2-2 elements in the zebrafish genome had extra nucleotides at the 5′ junction, whereas ∼50% had microhomologies [29]. At the 3′ junction, on the other hand, ∼80% of these elements had microhomologies [29]. Similar tendencies were observed at both junctions of ZfL2-2 insertions in DT40 cells, and these tendencies were not altered by NHEJ defects (Table S5). Also, the length distribution of the 5′ and 3′ microhomologies did not differ between the WT and NHEJ-deficient DT40 cells (Figure S15). However, the ZfL2-2 insertions in Ku70^−/−^ and Artemis^−/−^ cells were significantly longer than those in WT cells (Figure 3A and 3B; P = 0.008 and 0.036, respectively). In particular, full-length elements were recovered only from NHEJ-deficient cells (Figure 3A, 3C). Indeed, the fraction of full-length insertions differed significantly between WT and Ku70^−/−^ cells and between WT and Artemis^−/−^ cells (P = 0.010 and 0.046, respectively). These results indicate that Ku70 and Artemis inhibit the generation of longer inserts in DT40 cells, suggesting that these NHEJ factors, at least in part, participate in LINE 5′ truncation.

We classified the target site alterations of the ZfL2-2 insertions in DT40 cells into five categories: long TST (L-TST, >20 bp), short TST (S-TST, ≤20 bp), BEJ, short TSD (S-TSD, ≤20 bp) and long TSD (L-TSD, >20 bp) (Figure 3D, Table S5). Consistent with our previous data regarding ZfL2-2 elements present in the zebrafish genome [29], a large fraction of ZfL2-2 insertions (20 of 25, 80%) in WT DT40 had a TSD, and the rest of them had a TST (Figure 3D). Most insertions (24 of 25) in the WT cells had short target site alterations (≤20 bp), and only one had a L-TSD (1228 bp), indicating that long target site alterations are relatively rare in WT cells. On the other hand, L-TST (343–50187 bp) insertions were frequently observed in Ku70^−/−^ cells (Figure 3D; 5 of 25), suggesting that Ku70 prevents the generation of L-TST.

We next focused on insertions with short target site alterations (Figure 3E). Insertions with S-TSD predominated in all cell lines. Still, 5 of 25 insertions (20%) in WT cells and 3 of 20 insertions (15%) in Ku70^−/−^ cells had an S-TST. In contrast, only one of 23 insertions (4%) in Artemis^−/−^ cells had an S-TST, and no S-TSTs were observed in LigIV^−/−^ cells. The difference in occurrence of S-TSTs between the WT and LigIV^−/−^ cells is statistically significant (Figure 3E; P = 0.016). These results indicate that LigIV (and possibly Artemis) plays an important role in generating S-TSTs.

### Inhibition of DNA-PKcs Kinase Activity Decreases the ZfL2-2 and L1 RFs in HeLa Cells

**Figure 4:**
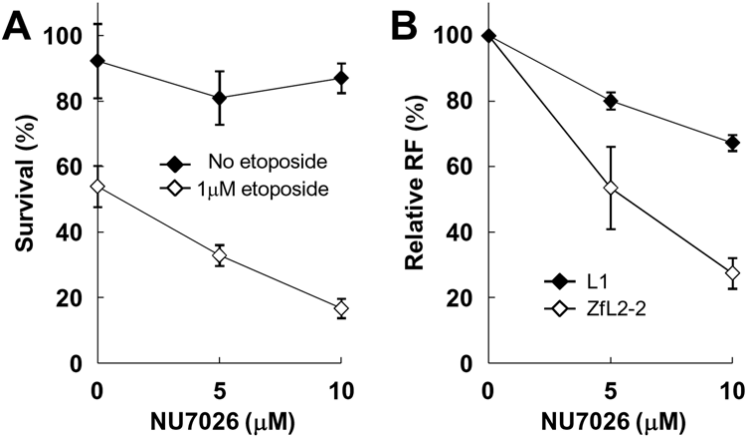
Retrotransposition assay in HeLa cells with NU7026. (A) Survival rate of HeLa cells treated with NU7026 in the presence or absence of etoposide. HeLa cells treated with these agents for 2 h were plated on a 100-mm plate. Three independent experiments were performed, and the means with standard deviations are shown. (B) The result of the retrotransposition assay in HeLa cells treated with NU7026. Retrotransposition frequency (RF) values are relative to those measured in the absence of NU7026. Two independent experiments were performed, and the means with standard deviations are shown.

To examine whether NHEJ is also involved in LINE retrotransposition in cells other than chicken DT40, we performed the retrotransposition assay in human HeLa cells (Figure 4). No knockout HeLa cell line is available, but DNA-PKcs kinase activity can be specifically inhibited by NU7026 [30]. We first confirmed that NU7026 kills HeLa cells in a dose-dependent manner only in the presence of a DSB inducer, etoposide [31]. HeLa cells treated with NU7026 became more sensitive to etoposide, indicating that the NHEJ repair capacity is suppressed by NU7026 (Figure 4A). The RFs of both ZfL2-2 and L1 decreased with increasing concentrations of NU7026, suggesting that the NHEJ pathway is also involved in LINE retrotransposition in HeLa cells (Figure 4B, Table S6, S7). Consistent with the results using DT40 cells, ZfL2-2 retrotransposition was more sensitive than L1 retrotransposition to NU7026.

## Discussion

Host repair systems are likely to be involved in the later stages of LINE retrotransposition [12],[13],[29],[32],[33]. For example, bioinformatic studies have suggested that the ‘alternative’ NHEJ pathway is involved in LINE retrotransposition [33] (see below). There is, however, no bioinformatic evidence for such involvement of the ‘classical’ NHEJ or experimental evidence for a role in LINE retrotransposition of any host repair system except for the ATM kinase [12]. Here, we studied the effects of defects in ‘classical’ NHEJ on ZfL2-2 retrotransposition and found that such defects considerably decrease the ZfL2-2 RF, suggesting that a large fraction of ZfL2-2 insertion events in DT40 cells utilizes these classical NHEJ factors. In addition, the characterization of ZfL2-2 insertions revealed that disruption of the genes encoding NHEJ components extended the length of inserted ZfL2-2 elements, allowing more full-length insertions (Ku70^−/−^ and Artemis^−/−^); frequently generated L-TSTs (Ku70^−/−^); and diminished the generation of S-TSTs (LigIV^−/−^). These results suggest that NHEJ proteins are involved in the 5′ joining of ZfL2-2 insertions during retrotransposition, as detailed below.

**Figure 5:**
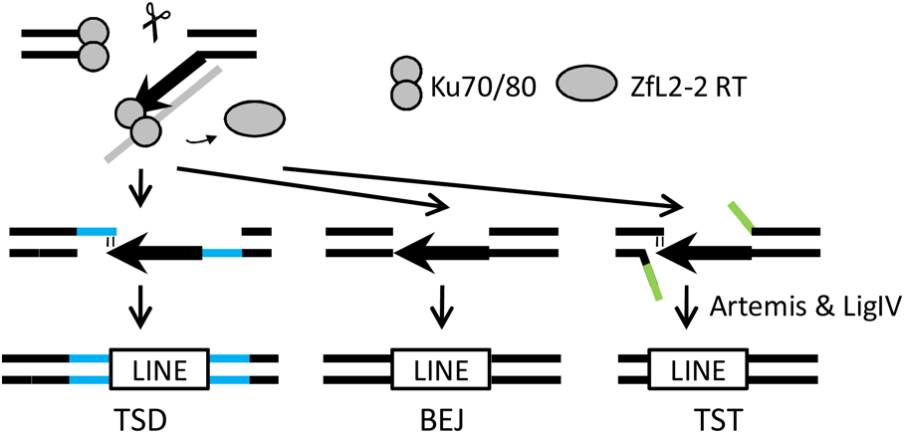
A model for ZfL2-2 integration. See Discussion for explanation of the model. TSD, target site duplication. BEJ, blunt end joining. TST, target site truncation. Blue lines denote chromosomal DNA that is duplicated in TSD. Green lines denote chromosomal DNA that is truncated in TST.

During retrotransposition (Figure S1A), the ZfL2-2 RNA-protein complex chooses a target site, at which the ZfL2-2 EN nicks the first strand of the host DNA. The ZfL2-2 RT then initiates reverse transcription of the ZfL2-2 RNA from the nick. Most ZfL2-2 elements in DT40 cells as well as those in the zebrafish genome have a certain length of truncation at the 5′ end (5′ truncation), which is a characteristic of a typical LINE element. The mechanism by which the 5′ truncation is generated is, however, unclear. Our data provide a possible mechanism for the 5′ truncation. The Ku70 defect produced longer insertions (Figure 3A, 3B), implying that the Ku70/80 complex can obstruct the progression of the ZfL2-2 RT. For instance, transient dissociation of the RT from the template RNA could allow Ku70/80 to associate with the end of the newly synthesized ZfL2-2 DNA (Figure 5) because Ku70/80 is able to interact with a single-to-double-strand transition of DNA [34]. The Ku70/80 association may interfere with further reverse transcription and initiate a joining reaction between the premature ZfL2-2 cDNA and upstream target DNA, resulting in a 5′ truncation. Because deficiencies of Artemis and LigIV—which act downstream of Ku70 in NHEJ—also caused longer insertions (Figure 3A, 3B), the progression of the NHEJ pathway might be related to the switching of reaction modes from reverse transcription to 5′ joining.

Ku70/80 protects DNA ends from exonucleolytic degradation [35]. Consistently, Ku70^−/−^ cells frequently produced ZfL2-2 insertions containing long chromosomal DNA deletions (Figure 3D; L-TST, 343–50187 bp). This suggests that Ku70/80 is associated with the end of the upstream target DNA as well as the end of the ZfL2-2 element during integration, and protects the chromosomal DNA from degradation (Figure 5). In the case of TST generation, genomic information is altered not only by inserting the ZfL2-2 sequence but also by deleting the pre-existing sequence. Thus, Ku70/80 may also serve as a barrier against the loss of genomic information caused by ZfL2-2 retrotransposition.

The variability of target site alteration has been accounted for by the difference in the position of the second strand cleavage [11] (Figure S1B); however, it remains unclear what other factor or factors are involved in this variation. We found that LigIV^−/−^ cells did not produce S-TSTs, whereas ∼20% of insertions in WT cells had an S-TST (P = 0.016), indicating that S-TST generation is dependent on LigIV activity. Moreover, the S-TST frequency was also decreased in Artemis^−/−^ cells. Therefore, the 5′ overhang (generated by the second-strand cleavage upstream of the first nick; see Figure S1B) at the chromosomal end may be processed predominantly by Artemis and then ligated to the ZfL2-2 5′ end by LigIV (Figure 5). Hence, our results suggest that these NHEJ factors contribute to variation among target site alterations.

Taken together, our data suggest the possibility that NHEJ proteins, originally recruited for the repair of chromosomal breaks generated by the ZfL2-2 EN, are necessarily utilized for ZfL2-2 integration. Deficiencies of NHEJ proteins remarkably decreased the ZfL2-2 RF, indicating that NHEJ proteins are required for efficient retrotransposition. On the other hand, these NHEJ factors restricted the generation of full-length ZfL2-2 copies by diverting initiated retrotransposition reactions toward the generation of truncated ZfL2-2 copies. The restriction of full-length copies that have the potential to undergo subsequent retrotransposition limits the amplification of ZfL2-2 copies in the next generation. Interestingly, Deininger's group showed that many more DSBs than retrotransposition events are generated by L1 EN expression in HeLa cells [12], suggesting that a considerable fraction of L1-induced DSBs are repaired without L1 insertions. Because DSBs are predominantly repaired by NHEJ in vertebrate cells, it is plausible that these L1-induced DSBs are fixed by NHEJ. Thus, the NHEJ pathway probably limits retrotransposition at two different phases: 1) inhibition of LINE cDNA integration itself by immediate repair of DSBs, resulting in direct limitation of retrotransposition and 2) production of truncated insertions, resulting in limitation of retrotransposition in the next generation. Because more active L1 elements produce longer insertions [36], the rapid or efficient progression of reverse transcription may counteract both of these NHEJ limitations. Thus, rapid cDNA synthesis prior to the operation of the NHEJ pathway may be vital for successful LINE amplification. Taken together, these observations indicate that there is opposition between DNA repair and LINE retrotransposition. Similarly, a retrotransposon conflict hypothesis has been proposed by Sawyer and Malik, in which NHEJ proteins are proposed to be hijacked for mobilization of Ty LTR retrotransposons or recruited to defend against them [37].

Our data show that disruption of the NHEJ pathway in DT40 cells did not completely suppress ZfL2-2 retrotransposition (Figure 1). Therefore, a pathway(s) other than classical NHEJ may exist to connect the ZfL2-2 integrants and the end of the target DNA at the 5′ junction. As proposed by Zingler *et al.* [33], one possibility is the ‘alternative’ NHEJ pathway, which joins two DNA ends via microhomology in a manner independent of NHEJ factors such as Ku70/80 and LigIV. However, the fraction of insertions having 5′ microhomology was not elevated in Ku70^−/−^ or LigIV^−/−^ cells (Table S5), and thus this putative mechanism cannot fully account for the observed residual Ku70- or LigIV-independent retrotransposition activity. Rather, the template-jump model [33], [38]–[43] seems more likely for the 5′ joining process, although the DNA ligase responsible remains unidentified. Indeed, a large proportion of extra nucleotides found at the 5′ junction of ZfL2-2 insertions in DT40 cells appears to be synthesized by the template jump reaction (unpublished data).

Cell death caused by LINE EN expression was not detected in chicken DT40 cells in our experimental system (Figure 2, S13 and S14), although it causes considerable cell death in human HeLa cells [12] (data not shown). Neither DT40 nor HeLa cells have detectable levels of p53, a tumor suppressor that induces apoptosis or cell cycle arrest against DNA damage [44],[45]. Thus, although a lack of p53 appears to confer tolerance to chromosomal instability in DT40 cells [44], it cannot explain the observed differential sensitivity to LINE EN between DT40 and HeLa cells. We thus speculate that this differential sensitivity may reflect the presence of an intrinsic LINE retrotransposition mechanism in each cell line. The fact that LINE retrotransposition in chicken DT40 and hamster CHO cells is differentially dependent on EN supports this idea. Hence, comparative analysis of the mechanism of LINE retrotransposition in different cell lines and organisms is indispensable for understanding the generality and specificity of the LINE amplification mechanism.

Deficiencies of NHEJ proteins in DT40 and HeLa cells also decreased the L1 RF, suggesting that NHEJ factors participate in retrotransposition of human L1 as well as zebrafish ZfL2-2 in these vertebrate cells. The degree of decrease in the L1 RF was, however, smaller than that for ZfL2-2 (Figure 1, 4), indicating that NHEJ is not much involved in retrotransposition of human L1. This implies that each LINE has its own dependency on NHEJ and probably other repair system(s); in other words, that the mechanism of LINE retrotransposition is considerably distinct between each LINE in the light of the participation of host repair systems. A major structural difference between these LINEs is the absence (ZfL2-2) or presence (L1) of ORF1p. Because L1 ORF1p has been suggested to be involved in the 5′ joining [46], ORF1p might make L1 more independent of the host NHEJ system. Our study indicates that the factors of classical NHEJ are involved in the repair of breaks generated by LINEs during retrotransposition. Our results also indicate that the NHEJ pathway is not the only mechanism by which such breaks can be repaired. Elucidation of the entire ensemble of host factors involved in LINE mobilization will help us understand the interaction between hosts and molecular parasites during evolution.

## Materials and Methods

### Expression Vectors

pBZ2-5 expresses the WT zebrafish LINE ZfL2-2 containing the neomycin resistance gene that is disrupted by an intron in the antisense orientation (*mneoI*) [25]. p131.11 expresses the *mneoI*-marked ZfL2-2 element containing a point mutation (E72A) in the EN sequence [22]. pAZ2-2, which expresses the WT ZfL2-2 element marked by *mneoI_400_/ColE1* [11], was constructed as follows. The *mneoI_400_/ColE1* cassette was amplified from pCEP4/L1.3*mneoI_400_/ColE1* [11] by PCR using primers Neo-NotF-1 (5′-TGTGTGTGGCGGCCGCGCACAAACGACCCAACACCC-3′) and Neo-BamR-1 (5′-CACACGGATCCGCTGCAGCATAGCCTCAGG-3′). The PCR fragment of *mneoI_400_/ColE1* was digested with NotI and BamHI. Using the *mneoI_400_/ColE1* fragment, the NotI and BamHI fragment of pBB4 [25], which contains *mneoI*, was replaced, resulting in pBB5-9. The 3′ tail of ZfL2-2 was amplified from pBZ2-5 by PCR using primers Z2-3′F1 (5′-ATATGGATCCTGAAACTTGCCTTTAGTACTTATTCATTGTTGC-3′) and Z2-3′R1 (5′-ATATGGATCCTTTACATTTACATTTACATTTAGTCATTTAGCAGACGC-3′). The PCR fragment of the 3′ tail was digested with BamHI and inserted in the BamHI site of pBB5-9, resulting in pAZ2-2. pJM102/L1.3 expresses the WT L1 (L1.3) containing the marker *mneoI* [27]. pJM102/L1.3 H230A expresses the *mneoI*-marked L1 (L1.3) containing a point mutation (H230A) in the EN sequence [27]. pEGFP-FLAG-1 expresses an enhanced green fluorescence protein (EGFP) [22]. Chicken Ku70/pAneo was constructed by cloning the chicken Ku70 gene into the expression vector pAneo [23]. The expression vectors were all purified using the QIAfilter Plasmid Midi or Mega kit (Qiagen).

### Cell Culture

WT DT40 and its SHIP1^−/−^ and IP3R^−/−^ derivatives were purchased from RIKEN Bioresource Center (cell numbers RCB1464, 1465, and 1467). Ku70^−/−^, DNA ligase IV^−/−^ and Rad18^−/−^ DT40 cell lines were established previously [23],[24],[47]. The Artemis^−/−^ DT40 cell line was kindly provided by Dr. Minoru Takata [48]. These DT40 cells were cultured in RPMI medium 1640 (Invitrogen) supplemented with 10% fetal bovine serum, 1% chicken serum, 20 U/ml penicillin, 20 µg/ml streptomycin, and 10 µM β-mercaptoethanol, in a humidified atmosphere with 5% CO_2_ at 37°C or at the temperatures indicated.

### Retrotransposition Assay in DT40 Cells

The retrotransposition assay procedure in DT40 cells has been described [22]. Briefly, DT40 cells were cotransfected with pEGFP-FLAG-1 (15 µg) and one of the LINE expression vectors (15 µg), pBZ2-2, p131.11, pJM102/L1.3, or pJM102/L1.3 H230A [22],[27]. Transfection was carried out by electroporation at 250 V and 960 µF for ZfL2-2 expression vectors, and at 200 V and 960 µF for L1 expression vectors using the GENE Pulser (Bio-Rad). After the transfected cells were incubated at 33°C for 3 days, the number of EGFP-positive and EGFP-negative cells were counted by flow cytometry to measure the transfection efficiency. To detect retrotransposition, the electroporated cells (∼1×10^6^ cells per dish) were plated in soft agarose medium containing G418 (1.6 mg/ml). In parallel, to determine the plating efficiency, the electroporated cells (200 cells per dish) were plated in soft agarose medium without G418. After an 11-day incubation at 37°C, visible colonies were counted. Plating efficiency was calculated as the number of visible colonies on the plate (without G418) as a percentage of the 200 cells plated. RF was calculated as: RF = G/(E×P/100), where G represents the number of G418-resistant colonies, E represents the number of EGFP-positive cells, and P represents the plating efficiency.

### Ku70 Complementation Assay

DT40 cells (WT, Ku70^−/−^ or LigIV^−/−^) were cotransfected with pEGFPFLAG-1 (10 µg), pBZ2-2 (10 µg), and one of two expression vectors (10 µg), chicken Ku70/pAneo or pAneo. Transfection was carried out by electroporation at 250 V and 960 µF using a GENE Pulser. After transfection, cells were processed by the same procedure described above, and the RF was calculated.

To detect transcription of Ku70 from the exogenous and/or endogenous gene, cells transfected with the three plasmid DNAs were harvested 3 days after transfection. Total RNA was extracted from the cells using the RNeasy Mini kit (Qiagen). The column-based preparation was repeated to avoid any DNA contamination. RT-PCR was performed using the total RNA (1 µg) as the template with primers cKu70R1 (5′-CAGAGACAGTGAGCTTGCCC-3′) and cKu70F2 (5′-CGCTGGATATGCTGGAACCA-3′). As a control, transcription of the chicken β-actin gene was detected by RT-PCR using primers cActinF1 (5′-GGTCAGGTCATCACCATTGG-3′) and cActinR1 (5′-TGCATCCTGTCAGCAATGCC-3′).

### Tracing of EGFP-Positive Cells

DT40 cells were cotransfected with pEGFP-FLAG-1 (15 µg) and one of the LINE expression vectors (15 µg), pBZ2-2, p131.11, pJM102/L1.3, or pJM102/L1.3 H230A [22],[27]. Transfection was carried out by electroporation at 200 V and 950 µF using the GENE Pulser Xcell (Bio-Rad). After electroporation, the cells were incubated at 33°C for 3 days. Then the cells were subcultured at 37°C. The percentage of EGFP-positive cells and their EGFP fluorescence intensity were monitored by flow cytometry at intervals of 12 h from 3 to 8 days after electroporation. Ten thousand cells were counted for each measurement by flow cytometry.

### Isolation of ZfL2-2 Insertions from DT40 Cells

Circular DNA containing ZfL2-2 insertions was isolated using the procedure developed by Gilbert *et al.* [11]. Briefly, DT40 cell clones derived from each G418-resistant colony produced by pAZ2-2 were cultured separately until the total number of cells reached ∼1×10^7^ per clone. Genomic DNA was isolated from each clone using the GenElute mammalian genomic DNA miniprep kit (Sigma). Genomic DNA (∼20 µg per clone) was digested with 75 U of HindIII for 6 h at 37°C. The digested DNA (∼20 µg) was then self-ligated overnight by T4 DNA ligase (350 U) in 500 µl solution at 16°C. Ninety percent of the circular DNA was incorporated in *E. coli* DH10BT1^R^ (Invitrogen) by electroporation with the GENE Pulser Xcell (Bio-Rad) under conditions of 2,500 V, 25 µF and 100 Ω, and the electroporated cells were plated on kanamycin-containing (70 µg/ml) plates. Circular DNA containing a *mneoI_400_/ColE1*-marked ZfL2-2 insertion (with its flanking chicken genomic DNA) was isolated from the kanamycin-resistant cells. The 5′ and 3′ junctions of each isolated ZfL2-2 insertion were sequenced using the appropriate primers. Sequences flanking each ZfL2-2 insertion were used as probes in BLAT searches to identify the preintegration site in the chicken genome database (http://genome.ucsc.edu; the May 2006 chicken (*Gallus gallus*) v2.1 draft assembly).

### Survival of HeLa Cells Treated with NU7026 and Etoposide

Exponentially growing HeLa-RC cells [25] were exposed to increasing concentrations of NU7026 with or without etoposide (1 µM) for 2 h. After treatment, the cells were trypsinized and reseeded into new 100-mm dishes at densities of 350 or 3,500 cells/dish and grown in fresh medium containing no drug. After 10 days, colonies were fixed with 100% ethanol and stained with 2% Giemsa solution. The survival rate was calculated as the number of colonies as a percentage of the reseeded cells.

### Retrotransposition Assay in HeLa Cells with NU7026

HeLa-RC cells (2×10^5^ cells/well) were seeded in 6-well dishes [25]. NU7026 of the indicated concentration was added to the medium 1 day after seeding. One hour after the addition of NU7026, the cells were transfected with 1 µg plasmid DNA (pBZ2-5 or pJM102/L1.3). The cells containing the plasmid were selected with hygromycin (200 µg/ml) for 6 days. NU7026 treatment was continued during the hygromycin selection. The hygromycin-resistant (Hyg^R^) cells were trypsinized and reseeded into new 100-mm dishes (at the density of 100,000 cells/dish for pBZ2-5 and 5,000 cells/dish for pJM102/L1.3) and grown in medium with 400 µg/ml G418. In parallel, 10,000 Hyg^R^ cells for pBZ2-5 or 2,000 Hyg^R^ cells for pJM102/L1.3 were also reseeded in a 100-mm dish and grown in medium without G418 to measure the plating efficiency. After a 12-day incubation, cell colonies were fixed by 100% ethanol and stained with 2% Giemsa solution. The plating efficiency was calculated as the number of visible colonies on the plate (without G418) as a percentage of the number of cells plated. RF was calculated as the number of G418-resistant colonies per Hyg^R^ cell, compensating for the plating efficiency.

## Supporting Information

Figure S1 A model for LINE retrotransposition. (A) An overview of the model. LINEs are transcribed into RNA from which the LINE-encoded protein is translated. The LINE RNA and protein form a RNA-protein complex (RNP). The LINE endonuclease in the RNP nicks the bottom strand of the target site DNA, and the LINE reverse transcriptase in the RNP reverse transcribes the LINE RNA using the 3′ hydroxyl group generated by the nick as a primer. This reaction is called target-primed reverse transcription (TPRT). The LINE DNA/RNA heteroduplex must then be converted to a DNA/DNA duplex and integrated into the target site. However, the molecular mechanism by which LINE retrotransposition is completed remains unclear. (B) A model for target site alterations. Variation in target site alterations is considered to arise from differences in the position of the second strand cleavage (Gilbert et al, *Cell* 110: 315–325, 2002). Second-strand cleavage downstream of the first-strand nick generates a target site duplication (TSD). Second-strand cleavage at the same site as the first-strand nick generates blunt end joining (BEJ). Second-strand cleavage upstream of the first-strand nick generates a target site truncation (TST). Blue lines denote the duplicated region in TSD. Green lines denote the truncated region in TST.
(0.03 MB PDF)

Figure S2 Retrotransposition assay in chicken DT40 cells. (A) Procedure for detection of LINE retrotransposition in DT40 cells. The retrotransposition detection cassette, *mneoI*, is inserted in the 3′ UTR of a LINE element. *mneoI* encodes the neomycin resistance gene (Neo), which is disrupted by an intron in the antisense orientation. The functional neomycin resistance protein is expressed only after the *mneoI*-marked LINE has been transcribed, spliced, and reverse transcribed into cDNA, which is then integrated into the chromosomal DNA of DT40 cells. pCMV, cytomegalovirus promoter. Pro, promoter. SVpA, SV40 polyA signal. pA, polyA signal. (B) Overview of LINE retrotransposition assay in DT40 cells. The LINE/*mneoI* expression vector and the EGFP expression vector are co-transfected into DT40 cells by electroporation. Three days after electroporation, the proportion of EGFP-expressing cells is measured as the transfection efficiency. At the same time, cells are plated in two kinds of soft agarose medium, one containing the antibiotic G418 and the other containing no antibiotic. Eleven days after plating, the number of colonies in the medium with no antibiotic is counted, and the plating efficiency is calculated from the colony number. The number of colonies in the G418-containing medium is also counted. The retrotransposition frequency is calculated as the number of G418 resistance (G418^R^) colonies per viable plated cell expressing EGFP (Materials and Methods). Small gray circles indicate EGFP-expressing cells. Small black circles indicate EGFP-expressing cells with G418^R^ (that is, containing a LINE integrant(s) in the genomic DNA).
(0.15 MB PDF)

Figure S3 A possible effect of the LINE EN expression on the retrotransposition frequency (RF) of Ku70^−/−^ cells calculated in the retrotransposition assay. Representative data from the ZfL2-2 retrotransposition assay in WT and Ku70^−/−^ cells are shown in each panel. (A) The case in which EN expression did not influence the viability of Ku70^−/−^ cells. (B) The case in which EN expression influenced the viability of Ku70^−/−^ cells. Although the values shown in (A) and (B) are identical, the RF of Ku70^−/−^ cells cannot be calculated properly (see below). Transfection efficiency - measured as the percentage of EGFP-positive cells 3 days after electroporation - was approximately 10% in Ku70^−/−^ cells (as well as WT cells), indicating that EN expression causes a maximum of only 10% decrease in the plating efficiency if EN causes severe death of Ku70^−/−^ cells. Thus, the plating efficiency of Ku70^−/−^ cells was scarcely altered by EN expression. On the other hand, the number of G418-resistant colonies was markedly decreased by the severe cell death caused by EN, indicating that the proper RF value in Ku70^−/−^ cells cannot be measured in the case of (B). However, the trace of EGFP-positive cells shown in Figure 2, S13 and S14 indicates that EN expression does not affect the cell viability of Ku70^−/−^ cells or Artemis^−/−^, LigIV^−/−^ and WT cells. We have not determined why the plating efficiency is different in each cell line, but the difference does not appear to be caused by LINE expression. Actually, the plating efficiency of untreated Ku70^−/−^ cells (no treatment with electroporation, G418, etc.) was ∼2-fold lower than that of untreated WT cells, suggesting that the colony-forming capability of these two cell types is fundamentally different in soft agarose medium (data not shown). In addition, manipulations of the retrotransposition assay, such as the G418 selection, may differentially affect the plating efficiency of each cell line.
(0.03 MB PDF)

Figure S4 Flow cytometric analysis of DT40 cells electroporated with fluorescence protein expression vectors. The red fluorescence protein (DsRed-Express) and green fluorescence protein (EGFP) expression vectors were co-electroporated into DT40 cells. Flow cytometric analysis was conducted 3 days after electroporation. Ten thousand cells were counted in one measurement. Fluorescence intensities of EGFP (FL1-H) and DsRed-Express (FL2-H) are shown. A dot shows a cell expressing no fluorescence protein (black), DsRed-Express only (red), EGFP only (green) or both of the fluorescence proteins (orange). R1 is defined as the region in which cells are expressing no fluorescence protein. R2 is defined as the region in which cells are expressing DsRed-Express only. R3 is defined as the region in which cells are expressing EGFP only. R4 is defined as the region in which cells are expressing both DsRed-Express and EGFP. (A) DT40 cells electroporated with no vector DNA. (B) DT40 cells electroporated with the DsRed-Express expression vector. (C) DT40 cells electroporated with the EGFP expression vector. (D) DT40 cells electroporated with both the DsRed-Express and EGFP expression vectors. (E) The percentage of cells presented in each region (R1-4) is shown. When the two kinds of plasmid DNAs are co-electroporated into DT40 cells, most transfected (fluorescence-positive) cells (>80%) express both of the two fluorescent proteins (Figure S4E). This indicates that both plasmids are usually introduced in each DT40 cell by electroporation. In addition, the fluorescence intensities of DsRed-Express and EGFP in a doubly transfected cell are roughly proportional to each other (Figure S4D), suggesting that the amounts of each plasmid introduced into a cell are positively related. Thus, when the EGFP and LINE expression plasmids are co-transfected into DT40 cells by electroporation, the fluorescence intensity of EGFP should be roughly proportional to the expression level of the LINE protein.
(0.12 MB PDF)

Figure S5 Flow cytometric analysis of WT DT40 cells co-electroporated with the EGFP and ZfL2-2 expression vectors. Expression of EGFP was measured from 3 to 8 days after electroporation. The histogram of the EGFP intensity (FL1-Height) is shown. The longitudinal axis shows the number of cells (Counts). The horizontal line in the histogram indicates the region defined as EGFP positive. GP, the percentage of EGFP-positive cells at each time point. Red arrowheads show the position of the geometric mean of the EGFP intensity (each value is indicated at the right of the arrowhead). Two independent experiments were conducted (A, B and C, D). (A, C) The flow cytometric data of WT DT40 cells without electroporation. (B, D) The flow cytometric data of WT DT40 cells electroporated with the EGFP expression vector and the ZfL2-2 wild-type (WT) or ZfL2-2 EN mutant (ENm) expression vector.
(2.71 MB PDF)

Figure S6 Flow cytometric analysis of WT and Ku70^−/−^ DT40 cells co-electroporated with the EGFP and ZfL2-2 expression vectors. Expression of EGFP was measured from 3 to 8 days after electroporation. The histogram of the EGFP intensity (FL1-Height) is shown. The longitudinal axis shows the number of cells (Counts). The horizontal line in the histogram indicates the region defined as EGFP positive. GP, the percentage of EGFP-positive cells at each time point. Red arrowheads show the position of the geometric mean of the EGFP intensity (each value is indicated at the right of the arrowhead). Two independent experiments were conducted (A, B and C, D). (A, C) Flow cytometric data of WT and Ku70^−/−^ DT40 cells without electroporation. (B, D) The flow cytometric data of WT and Ku70^−/−^ DT40 cells electroporated with the EGFP expression vector and the ZfL2-2 wild-type (WT) or ZfL2-2 EN mutant (ENm) expression vector.
(2.71 MB PDF)

Figure S7 Flow cytometric analysis of WT and Artemis^−/−^ DT40 cells co-electroporated with the EGFP and ZfL2-2 expression vectors. Expression of EGFP was measured from 3 to 8 days after electroporation. The histogram of the EGFP intensity (FL1-Height) is shown. The longitudinal axis shows the number of cells (Counts). The horizontal line in the histogram indicates the region defined as EGFP positive. GP, the percentage of EGFP-positive cells at each time point. Red arrowheads show the position of the geometric mean of the EGFP intensity (each value is indicated at the right of the arrowhead). Two independent experiments were conducted (A, B and C, D). (A, C) The flow cytometric data of WT and Artemis^−/−^ DT40 cells without electroporation. (B, D) The flow cytometric data of WT and Artemis^−/−^ DT40 cells electroporated with the EGFP expression vector and the ZfL2-2 wild-type (WT) or ZfL2-2 EN mutant (ENm) expression vector.
(2.71 MB PDF)

Figure S8 Flow cytometric analysis of WT and LigaseIV^−/−^ DT40 cells co-electroporated with the EGFP and ZfL2-2 expression vectors. Expression of EGFP was measured from 3 to 8 days after electroporation. The histogram of the EGFP intensity (FL1-Height) is shown. The longitudinal axis shows the number of cells (Counts). The horizontal line in the histogram indicates the region defined as EGFP positive. GP, the percentage of EGFP-positive cells at each time point. Red arrowheads show the position of the geometric mean of the EGFP intensity (each value is indicated at the right of the arrowhead). Two independent experiments were conducted (A, B and C, D). (A, C) The flow cytometric data of WT and LigaseIV^−/−^ DT40 cells without electroporation. (B, D) The flow cytometric data of WT and LigaseIV^−/−^ DT40 cells electroporated with the EGFP expression vector and the ZfL2-2 wild-type (WT) or ZfL2-2 EN mutant (ENm) expression vector.
(2.71 MB PDF)

Figure S9 Flow cytometric analysis of WT DT40 cells co-electroporated with the EGFP and L1 expression vectors. Expression of EGFP was measured from 3 to 8 days after electroporation. The histogram of the EGFP intensity (FL1-Height) is shown. The longitudinal axis shows the number of cells (Counts). The horizontal line in the histogram indicates the region defined as EGFP positive. GP, the percentage of EGFP-positive cells at each time point. Red arrowheads show the position of the geometric mean of the EGFP intensity (each value is indicated at the right of the arrowhead). Two independent experiments were conducted (A, B and C, D). (A, C) The flow cytometric data of WT DT40 cells without electroporation. (B, D) The flow cytometric data of WT DT40 cells electroporated with the EGFP expression vector and the L1 wild-type (WT) or L1 EN mutant (ENm) expression vector.
(2.71 MB PDF)

Figure S10 Flow cytometric analysis of WT and Ku70^−/−^ DT40 cells co-electroporated with the EGFP and L1 expression vectors. Expression of EGFP was measured from 3 to 8 days after electroporation. The histogram of the EGFP intensity (FL1-Height) is shown. The longitudinal axis shows the number of cells (Counts). The horizontal line in the histogram indicates the region defined as EGFP positive. GP, the percentage of EGFP-positive cells at each time point. Red arrowheads show the position of the geometric mean of the EGFP intensity (each value is indicated at the right of the arrowhead). Two independent experiments were conducted (A, B and C, D). (A, C) The flow cytometric data of WT and Ku70^−/−^ DT40 cells without electroporation. (B, D) The flow cytometric data of WT and Ku70^−/−^ DT40 cells electroporated with the EGFP expression vector and the L1 wild-type (WT) or L1 EN mutant (ENm) expression vector.
(2.72 MB PDF)

Figure S11 Flow cytometric analysis of WT and Artemis^−/−^ DT40 cells co-electroporated with the EGFP and L1 expression vectors. Expression of EGFP was measured from 3 to 8 days after electroporation. The histogram of the EGFP intensity (FL1-Height) is shown. The longitudinal axis shows the number of cells (Counts). The horizontal line in the histogram indicates the region defined as EGFP positive. GP, the percentage of EGFP-positive cells at each time point. Red arrowheads show the position of the geometric mean of the EGFP intensity (each value is indicated at the right of the arrowhead). Two independent experiments were conducted (A, B and C, D). (A, C) The flow cytometric data of WT and Artemis^−/−^ DT40 cells without electroporation. (B, D) The flow cytometric data of WT and Artemis^−/−^ DT40 cells electroporated with the EGFP expression vector and the L1 wild-type (WT) or L1 EN mutant (ENm) expression vector.
(2.72 MB PDF)

Figure S12 Flow cytometric analysis of WT and LigaseIV^−/−^ DT40 cells co-electroporated with the EGFP and L1 expression vectors. Expression of EGFP was measured from 3 to 8 days after electroporation. The histogram of the EGFP intensity (FL1-Height) is shown. The longitudinal axis shows the number of cells (Counts). The horizontal line in the histogram indicates the region defined as EGFP positive. GP, the percentage of EGFP-positive cells at each time point. Red arrowheads show the position of the geometric mean of the EGFP intensity (each value is indicated at the right of the arrowhead). Two independent experiments were conducted (A, B and C, D). (A, C) The flow cytometric data of WT and LigaseIV^−/−^ DT40 cells without electroporation. (B, D) The flow cytometric data of WT and LigaseIV^−/−^ DT40 cells electroporated with the EGFP expression vector and the L1 wild-type (WT) or L1 EN mutant (ENm) expression vector.
(2.72 MB PDF)

Figure S13 Effect of LINE expression on DT40 cell viability. DT40 cells were co-transfected with pEGFPFLAG-1 and one of the LINE expression vectors (pBZ2-5, p131.11, pJM102/L1.3, or pJM102/L1.3 H230A) by electroporation (see *Tracing of EGFP-positive cells* in the Materials and Methods section). After transfection, the cells were monitored for 8 days. (A) ZfL2-2 expression in DT40 cells. The relative proportion of EGFP-expressing cells (left), the geometric mean of the EGFP fluorescence intensity (FI) (middle) and the median of the EGFP FI (right) calculated using the values 3 days after electroporation as the standard are indicated (the raw data are shown in Figures S5 and S7). DT40 WT, wild-type DT40 cell line. DT40 Art^−/−^ Artemis-deficient DT40 cell line. ZfL2-2 WT, wild-type ZfL2-2 element. ZfL2-2 ENm, endonuclease-mutated ZfL2-2 elements. Two independent experiments were performed (upper and lower panels). (B) L1 expression in DT40 cells. The relative proportion of EGFP-expressing cells (left), the geometric mean of the EGFP FI (middle) and the median of the EGFP FI (right) calculated using the values 3 days after electroporation as the standard are indicated (the raw data are shown in Figures S9 and S11). DT40 WT, wild-type DT40 cell line. DT40 Art^−/−^, Artemis-deficient DT40 cell line. L1 WT, wild-type L1 element. L1 ENm, endonuclease-mutated L1 elements. Two independent experiments were performed (upper and lower panels).
(0.07 MB PDF)

Figure S14 Effect of LINE expression on DT40 cell viability. DT40 cells were co-transfected with pEGFPFLAG-1 and one of the LINE expression vectors (pBZ2-5, p131.11, pJM102/L1.3, or pJM102/L1.3 H230A) by electroporation (see *Tracing of EGFP-positive cells* in the Materials and Methods section). After transfection, the cells were monitored for 8 days. (A) ZfL2-2 expression in DT40 cells. The relative proportion of EGFP-expressing cells (left), the geometric mean of the EGFP fluorescence intensity (FI) (middle) and the median of the EGFP FI (right) calculated using the values 3 days after electroporation as the standard are indicated (the raw data are shown in Figures S5 and S8). DT40 WT, wild-type DT40 cell line. DT40 LigIV^−/−^, LigaseIV-deficient DT40 cell line. ZfL2-2 WT, wild-type ZfL2-2 element. ZfL2-2 ENm, endonuclease-mutated ZfL2-2 elements. Two independent experiments were performed (upper and lower panels). (B) L1 expression in DT40 cells. The relative proportion of EGFP-expressing cells (left), the geometric mean of the EGFP FI (middle) and the median of the EGFP FI (right) calculated using the values 3 days after electroporation as the standard are indicated (the raw data are shown in Figures S9 and S12). DT40 WT, wild-type DT40 cell line. DT40 LigIV^−/−^, LigaseIV-deficient DT40 cell line. L1 WT, wild-type L1 element. L1 ENm, endonuclease-mutated L1 elements. Two independent experiments were performed (upper and lower panels).
(0.07 MB PDF)

Figure S15 Length distributions of the 5′ and 3′ microhomologies of ZfL2-2 insertions in DT40 cells. All junctions except those with extra nucleotides are shown. (A) The length distribution of the 5′ microhomology. (B) The length distribution of the 3′ microhomology.
(0.01 MB PDF)

Table S1 ZfL2-2 retrotransposition in DT40 cells.
(0.05 MB DOC)

Table S2 L1 retrotransposition in DT40 cells.
(0.05 MB DOC)

Table S3 Ku70 complementation assay with ZfL2-2 in DT40 cells.
(0.04 MB DOC)

Table S4 The 102 ZfL2-2 insertions in chicken DT40 cells.
(0.18 MB DOC)

Table S5 Summary of ZfL2-2 insertions in chicken DT40 cells.
(0.05 MB DOC)

Table S6 ZfL2-2 retrotransposition assay in HeLa cells with NU7026.
(0.04 MB DOC)

Table S7 L1 retrotransposition assay in HeLa cells with NU7026.
(0.04 MB DOC)

## References

[1] Arkhipova I, Meselson M (2000). Transposable elements in sexual and ancient asexual taxa.. Proc Natl Acad Sci USA.

[2] Ohshima K, Okada N (2005). SINEs and LINEs: symbionts of eukaryotic genomes with a common tail.. Cytogenet Genome Res.

[3] Ostertag EM, Kazazian HH (2001). Biology of mammalian L1 retrotransposons.. Annu Rev Genet.

[4] Kazazian HH (2004). Mobile elements: drivers of genome evolution.. Science.

[5] Feng Q, Moran JV, Kazazian HH, Boeke JD (1996). Human L1 retrotransposon encodes a conserved endonuclease required for retrotransposition.. Cell.

[6] Moran JV, Holmes SE, Naas TP, DeBerardinis RJ, Boeke JD (1996). High frequency retrotransposition in cultured mammalian cells.. Cell.

[7] Kulpa DA, Moran JV (2006). Cis-preferential LINE-1 reverse transcriptase activity in ribonucleoprotein particles.. Nat Struct Mol Biol.

[8] Matsumoto T, Hamada M, Osanai M, Fujiwara H (2006). Essential domains for ribonucleoprotein complex formation required for retrotransposition of telomere-specific non-long terminal repeat retrotransposon SART1.. Mol Cell Biol.

[9] Luan DD, Korman MH, Jakubczak JL, Eickbush TH (1993). Reverse transcription of R2Bm RNA is primed by a nick at the chromosomal target site: a mechanism for non-LTR retrotransposition.. Cell.

[10] Cost GJ, Feng Q, Jacquier A, Boeke JD (2002). Human L1 element target-primed reverse transcription in vitro.. EMBO J.

[11] Gilbert N, Lutz-Prigge S, Moran JV (2002). Genomic deletions created upon LINE-1 retrotransposition.. Cell.

[12] Gasior SL, Wakeman TP, Xu B, Deininger PL (2006). The human LINE-1 retrotransposon creates DNA double-strand breaks.. J Mol Biol.

[13] Beauregard A, Curcio MJ, Belfort M (2008). The take and give between retrotransposable elements and their hosts.. Annu Rev Genet.

[14] Gasior SL, Roy-Engel AM, Deininger PL (2008). ERCC1/XPF limits L1 retrotransposition.. DNA repair.

[15] Burma S, Chen BP, Chen DJ (2006). Role of non-homologous end joining (NHEJ) in maintaining genomic integrity.. DNA Repair (Amst).

[16] Spagnolo L, Rivera-Calzada A, Pearl LH, Llorca O (2006). Three-dimensional structure of the human DNA-PKcs/Ku70/Ku80 complex assembled on DNA and its implications for DNA DSB repair.. Mol Cell.

[17] Douglas P, Gupta S, Morrice N, Meek K, Lees-Miller SP (2005). DNA-PK-dependent phosphorylation of Ku70/80 is not required for non-homologous end joining.. DNA Repair (Amst).

[18] Grawunder U, Wilm M, Wu X, Kulesza P, Wilson TE (1997). Activity of DNA ligase IV stimulated by complex formation with XRCC4 protein in mammalian cells.. Nature.

[19] Grawunder U, Zimmer D, Fugmann S, Schwarz K, Lieber MR (1998). DNA ligase IV is essential for V(D)J recombination and DNA double-strand break repair in human precursor lymphocytes.. Mol Cell.

[20] Roth DB (2003). Restraining the V(D)J recombinase.. Nat Rev Immunol.

[21] Yan CT, Boboila C, Souza EK, Franco S, Hickernell TR (2007). IgH class switching and translocations use a robust non-classical end-joining pathway.. Nature.

[22] Honda H, Ichiyanagi K, Suzuki J, Ono T, Koyama H (2007). A new system for analyzing LINE retrotransposition in the chicken DT40 cell line widely used for reverse genetics.. Gene.

[23] Takata M, Sasaki MS, Sonoda E, Morrison C, Hashimoto M (1998). Homologous recombination and non-homologous end-joining pathways of DNA double-strand break repair have overlapping roles in the maintenance of chromosomal integrity in vertebrate cells.. EMBO J.

[24] Adachi N, Ishino T, Ishii Y, Takeda S, Koyama H (2001). DNA ligase IV-deficient cells are more resistant to ionizing radiation in the absence of Ku70: Implications for DNA double-strand break repair.. Proc Natl Acad Sci USA.

[25] Sugano T, Kajikawa M, Okada N (2006). Isolation and characterization of retrotransposition-competent LINEs from zebrafish.. Gene.

[26] Kajikawa M, Okada N (2002). LINEs mobilize SINEs in the eel through a shared 3′ sequence.. Cell.

[27] Morrish TA, Gilbert N, Myers JS, Vincent BJ, Stamato TD (2002). DNA repair mediated by endonuclease-independent LINE-1 retrotransposition.. Nat Genet.

[28] Morrish TA, Garcia-Perez JL, Stamato TD, Taccioli GE, Sekiguchi J (2007). Endonuclease-independent LINE-1 retrotransposition at mammalian telomeres.. Nature.

[29] Ichiyanagi K, Nakajima R, Kajikawa M, Okada N (2007). Novel retrotransposon analysis reveals multiple mobility pathways dictated by hosts.. Genome Res.

[30] Veuger SJ, Curtin NJ, Richardson CJ, Smith GC, Durkacz BW (2003). Radiosensitization and DNA repair inhibition by the combined use of novel inhibitors of DNA-dependent protein kinase and poly(ADP-ribose) polymerase-1.. Cancer Res.

[31] Liu LF (1989). DNA topoisomerase poisons as antitumor drugs.. Annu Rev Biochem.

[32] Gilbert N, Lutz S, Morrish TA, Moran JV (2005). Multiple fates of L1 retrotransposition intermediates in cultured human cells.. Mol Cell Biol.

[33] Zingler N, Willhoeft U, Brose HP, Schoder V, Jahns T (2005). Analysis of 5′ junctions of human LINE-1 and Alu retrotransposons suggests an alternative model for 5′-end attachment requiring microhomology-mediated end-joining.. Genome Res.

[34] Dynan WS, Yoo S (1998). Interaction of Ku protein and DNA-dependent protein kinase catalytic subunit with nucleic acids.. Nucleic Acids Res.

[35] Guirouilh-Barbat J, Huck S, Bertrand P, Pirzio L, Desmaze C (2004). Impact of the KU80 pathway on NHEJ-induced genome rearrangements in mammalian cells.. Mol Cell.

[36] Farley AH, Luning Prak ET, Kazazian HH (2004). More active human L1 retrotransposons produce longer insertions.. Nucleic Acids Res.

[37] Sawyer SL, Malik HS (2006). Positive selection of yeast nonhomologous end-joining genes and a retrotransposon conflict hypothesis.. Proc Natl Acad Sci USA.

[38] George JA, Burke WD, Eickbush TH (1996). Analysis of the 5′ junctions of R2 insertions with the 28S gene: Implications for non-LTR retrotransposition.. Genetics.

[39] Burke WD, Malik HS, Jones JP, Eickbush TH (1999). The domain structure and retrotransposition mechanism of R2 elements are conserved throughout arthropods.. Mol Biol Evol.

[40] Bibillo A, Eickbush TH (2002). High processivity of the reverse transcriptase from a non-long terminal repeat retrotransposon.. J Biol Chem.

[41] Bibillo A, Eickbush TH (2002). The reverse transcriptase of the R2 non-LTR retrotransposon: continuous synthesis of cDNA on non-continuous RNA templates.. J Mol Biol.

[42] Bibillo A, Eickbush TH (2004). End-to-end template jumping by the reverse transcriptase encoded by the R2 retrotransposon.. J Bio Chem.

[43] Babushok DV, Ostertag EM, Courtney CE, Choi JM, Kazazian HH (2006). L1 integration in a transgenic mouse model.. Genome Res.

[44] Yamazoe M, Sonoda E, Hochegger H, Takeda S (2004). Reverse genetic studies of the DNA damage response in the chicken B lymphocyte line DT40.. DNA Repair.

[45] Böhnke A, Westphal F, Schmidt A, El-Awady RA, Dahm-Daphi J (2004). Role of p53 mutations, protein function and DNA damage for the radiosensitivity of human tumour cells.. Int J Radiat Biol.

[46] Martin SL, Bushman FD (2001). Nucleic acid chaperone activity of the ORF1 protein from the mouse LINE-1 retrotransposon.. Mol Cell Biol.

[47] Yamashita YM, Okada T, Matsusaka T, Sonoda E, Zhao GY (2002). RAD18 and RAD54 cooperatively contribute to maintenance of genomic stability in vertebrate cells.. EMBO J.

[48] Ishiai M, Kimura M, Namikoshi K, Yamazoe M, Yamamoto K (2004). DNA cross-link repair protein SNM1A interacts with PIAS1 in nuclear focus formation.. Mol Cell Biol.

