# Supplementary figures and images for: Genetic Evidence That the Non-Homologous End-Joining Repair Pathway Is Involved in LINE Retrotransposition
Source: PLoS Genet. 2009 Apr 24;5(4):e1000461. doi: 10.1371/journal.pgen.1000461 (PMC2666801; doi:10.1371/journal.pgen.1000461)

A

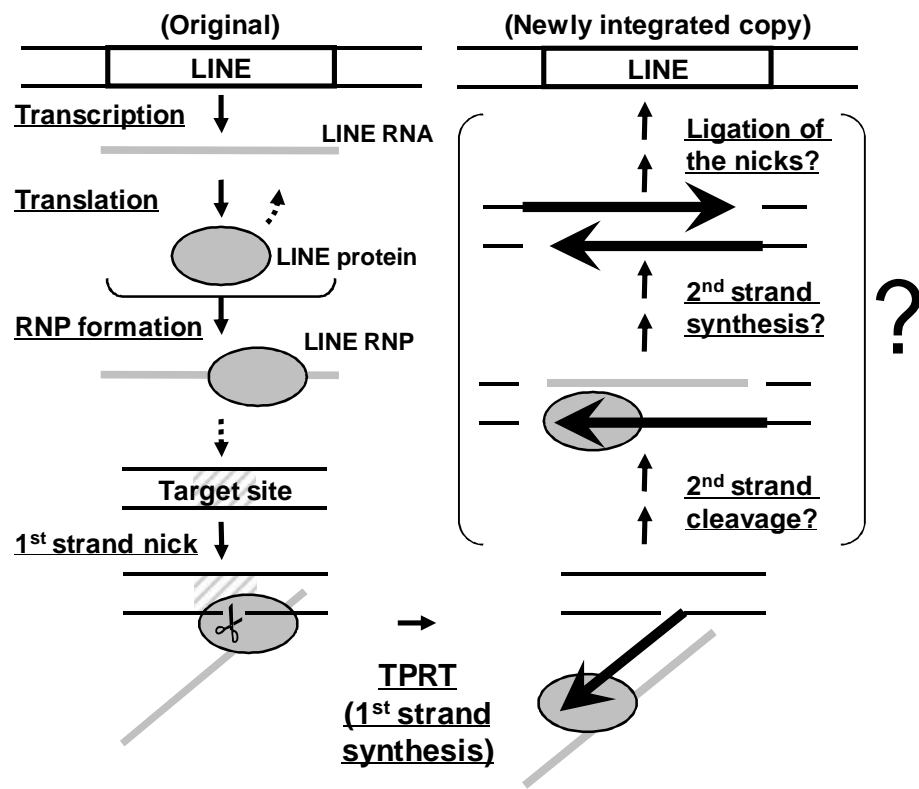

B

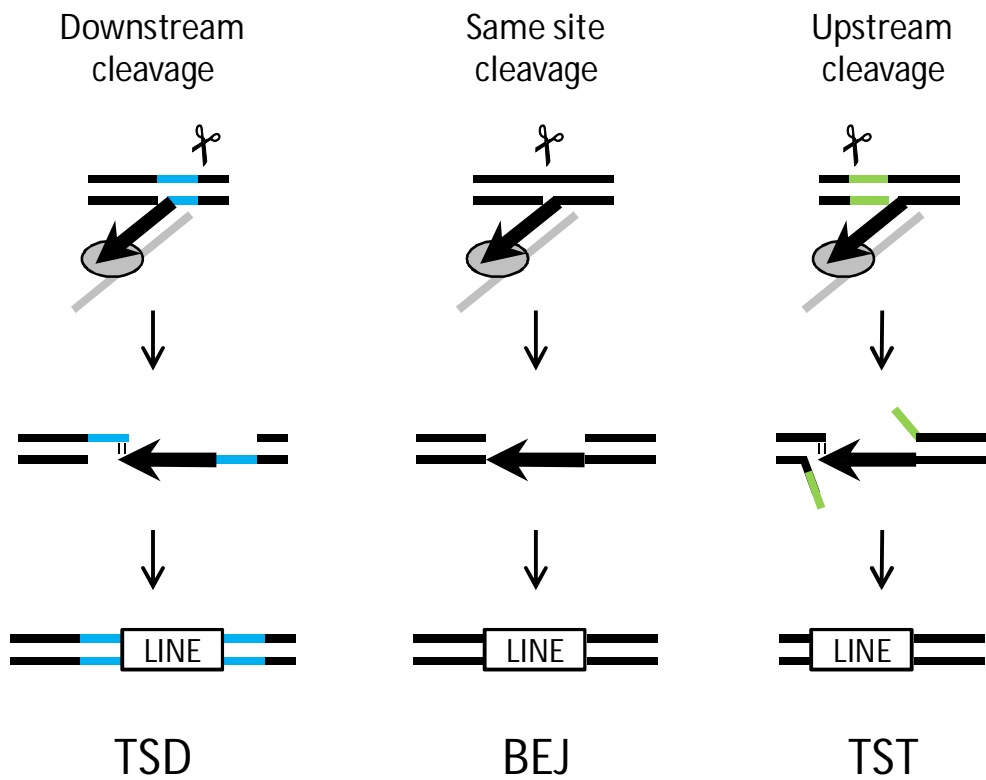

Supplement: Figure S1 — A model for LINE retrotransposition. (A) An overview of the model. LINEs are transcribed into RNA from which the LINE-encoded protein is translated. The LINE RNA and protein form a RNA-protein complex (RNP). The LINE endonuclease in the RNP nicks the bottom strand of the target site DNA, and the LINE reverse transcriptase in the RNP reverse transcribes the LINE RNA using the 3′ hydroxyl group generated by the nick as a primer. This reaction is called target-primed reverse transcription (TPRT). The LINE DNA/RNA heteroduplex must then be converted to a DNA/DNA duplex and integrated into the target site. However, the molecular mechanism by which LINE retrotransposition is completed remains unclear. (B) A model for target site alterations. Variation in target site alterations is considered to arise from differences in the position of the second strand cleavage (Gilbert et al, Cell 110: 315–325, 2002). Second-strand cleavage downstream of the first-strand nick generates a target site duplication (TSD). Second-strand cleavage at the same site as the first-strand nick generates blunt end joining (BEJ). Second-strand cleavage upstream of the first-strand nick generates a target site truncation (TST). Blue lines denote the duplicated region in TSD. Green lines denote the truncated region in TST. (0.03 MB PDF) [file pgen.1000461.s001.pdf]

A

DT40 WT / no plasmid

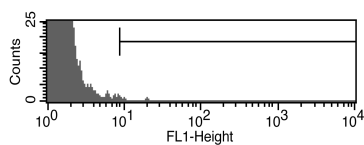

B

DT40 WT / Zfl2-2 WT

DT40 WT / Zfl2-2 ENm

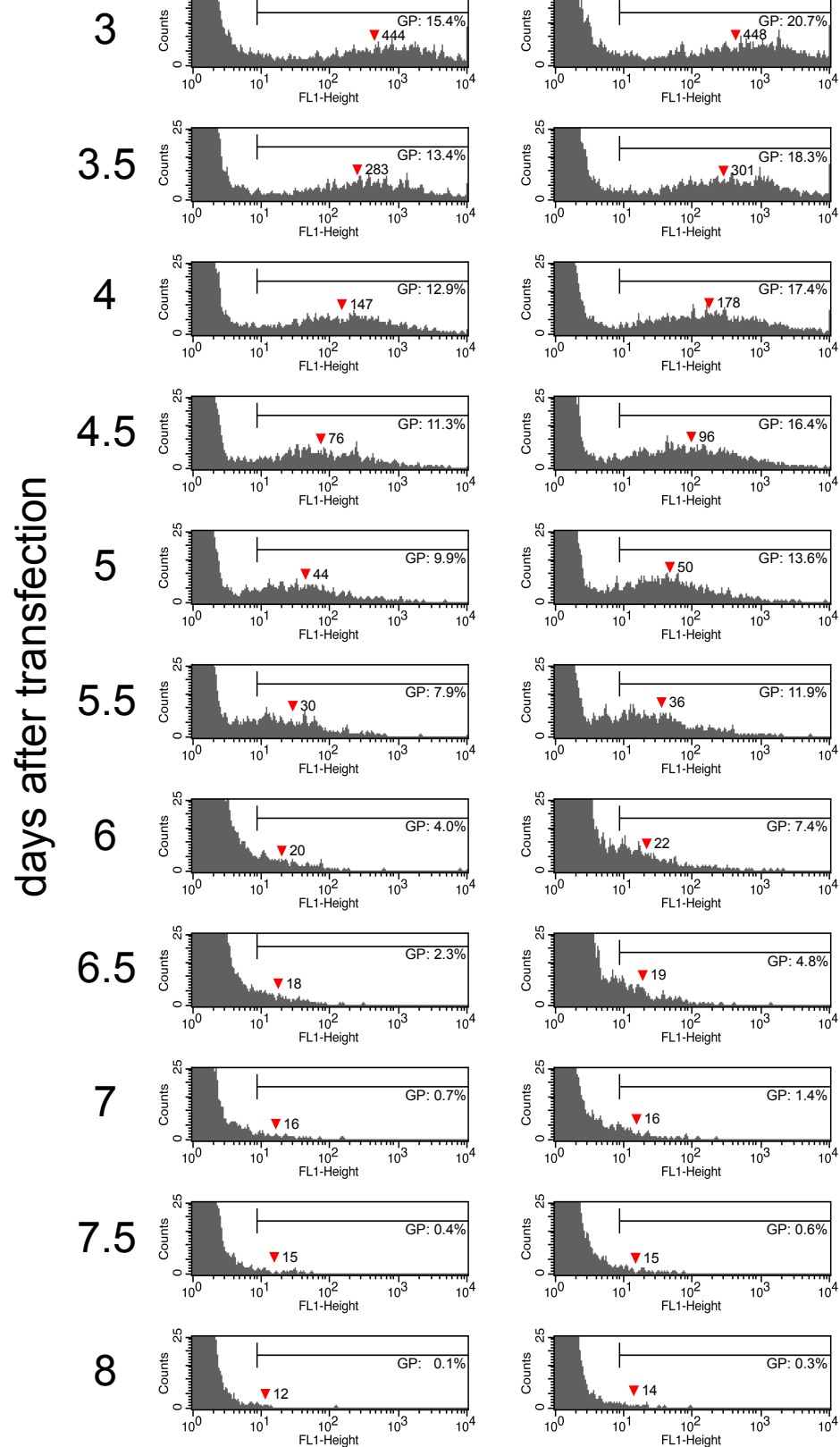

C

DT40 WT / no plasmid

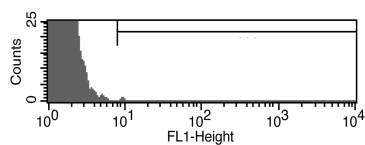

D

DT40 WT / Zfl2-2 WT

DT40 WT / Zfl2-2 ENm

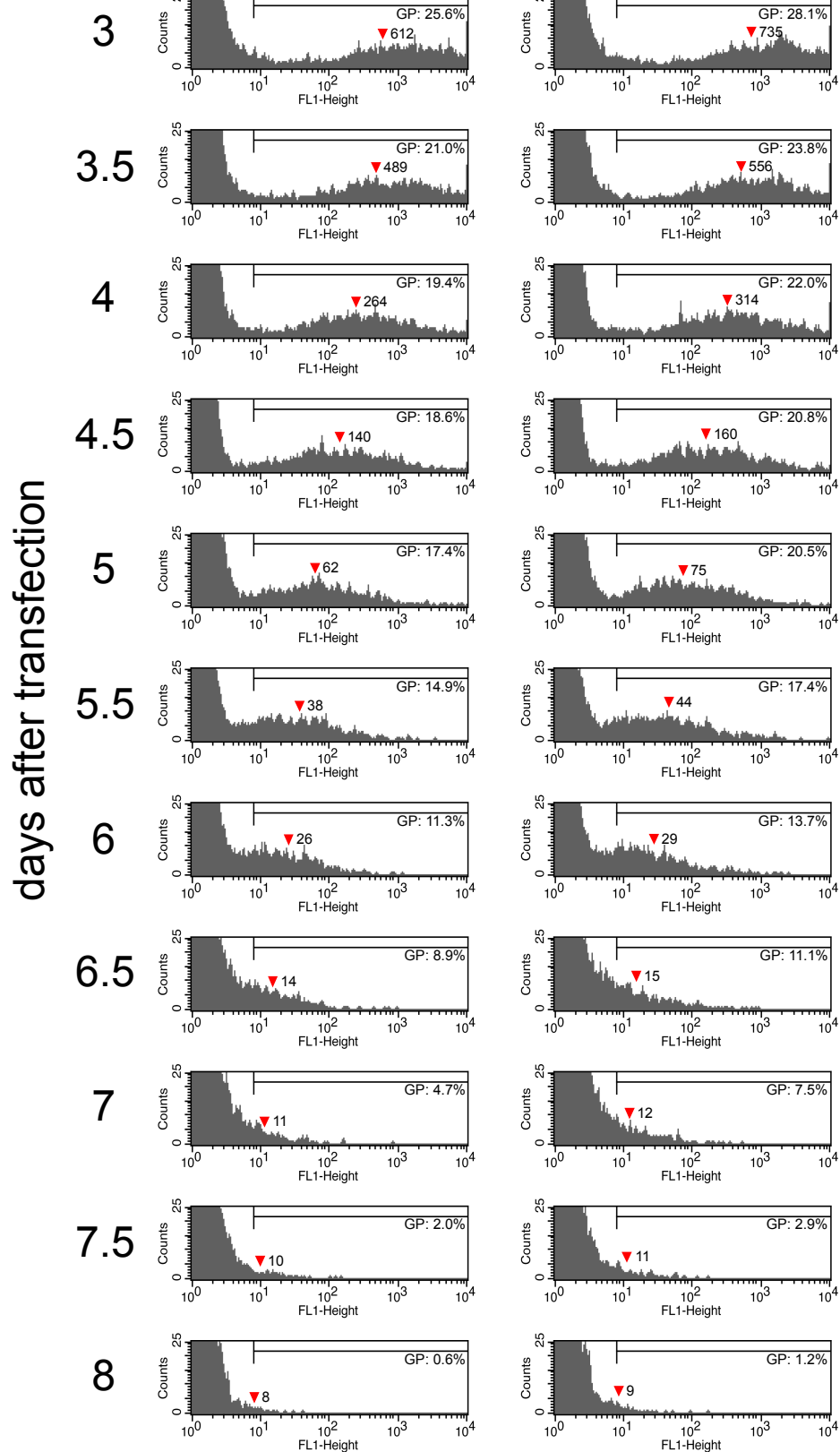

Supplement: Figure S5 — Flow cytometric analysis of WT DT40 cells co-electroporated with the EGFP and ZfL2-2 expression vectors. Expression of EGFP was measured from 3 to 8 days after electroporation. The histogram of the EGFP intensity (FL1-Height) is shown. The longitudinal axis shows the number of cells (Counts). The horizontal line in the histogram indicates the region defined as EGFP positive. GP, the percentage of EGFP-positive cells at each time point. Red arrowheads show the position of the geometric mean of the EGFP intensity (each value is indicated at the right of the arrowhead). Two independent experiments were conducted (A, B and C, D). (A, C) The flow cytometric data of WT DT40 cells without electroporation. (B, D) The flow cytometric data of WT DT40 cells electroporated with the EGFP expression vector and the ZfL2-2 wild-type (WT) or ZfL2-2 EN mutant (ENm) expression vector. (2.71 MB PDF) [file pgen.1000461.s005.pdf]

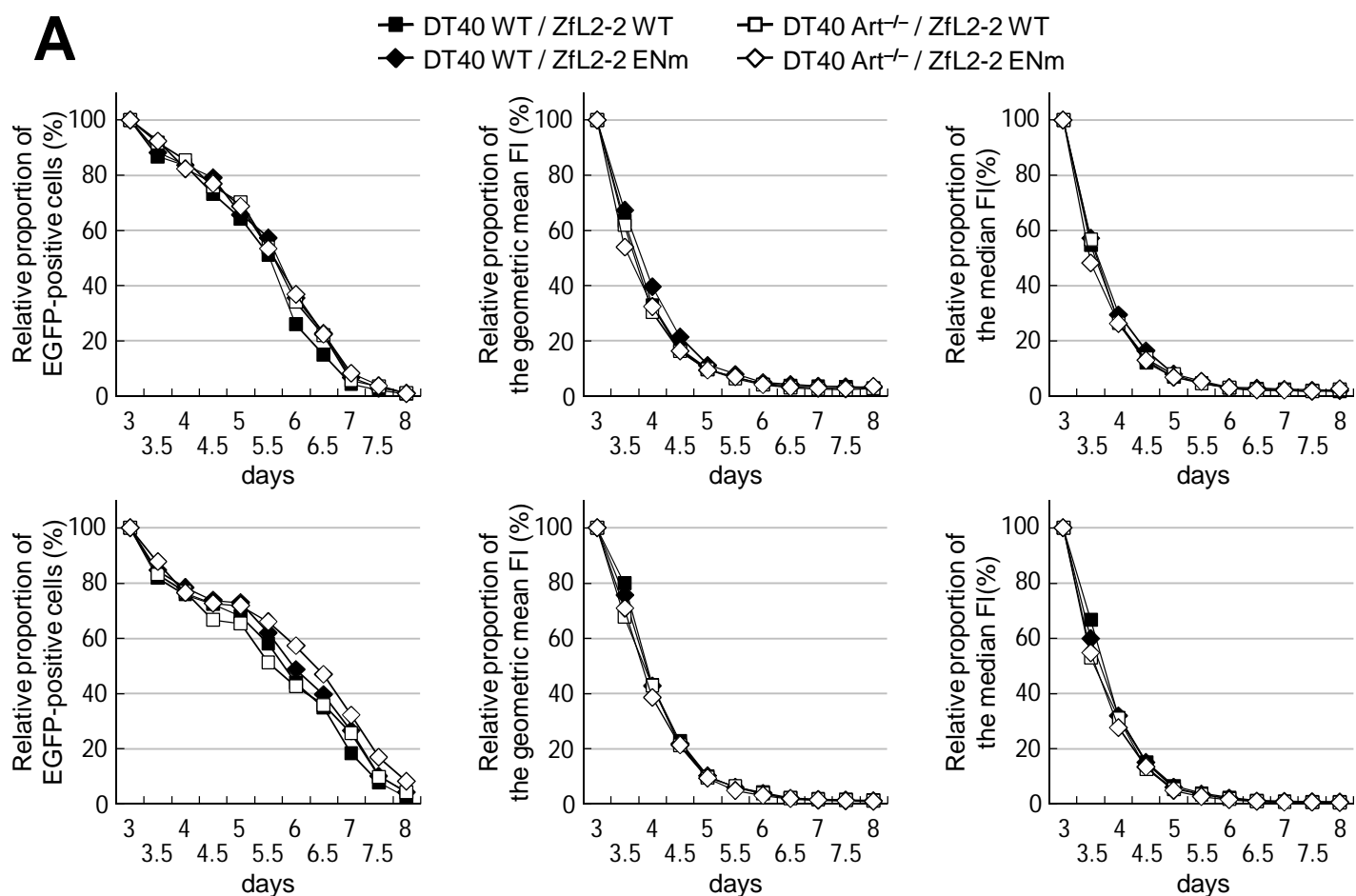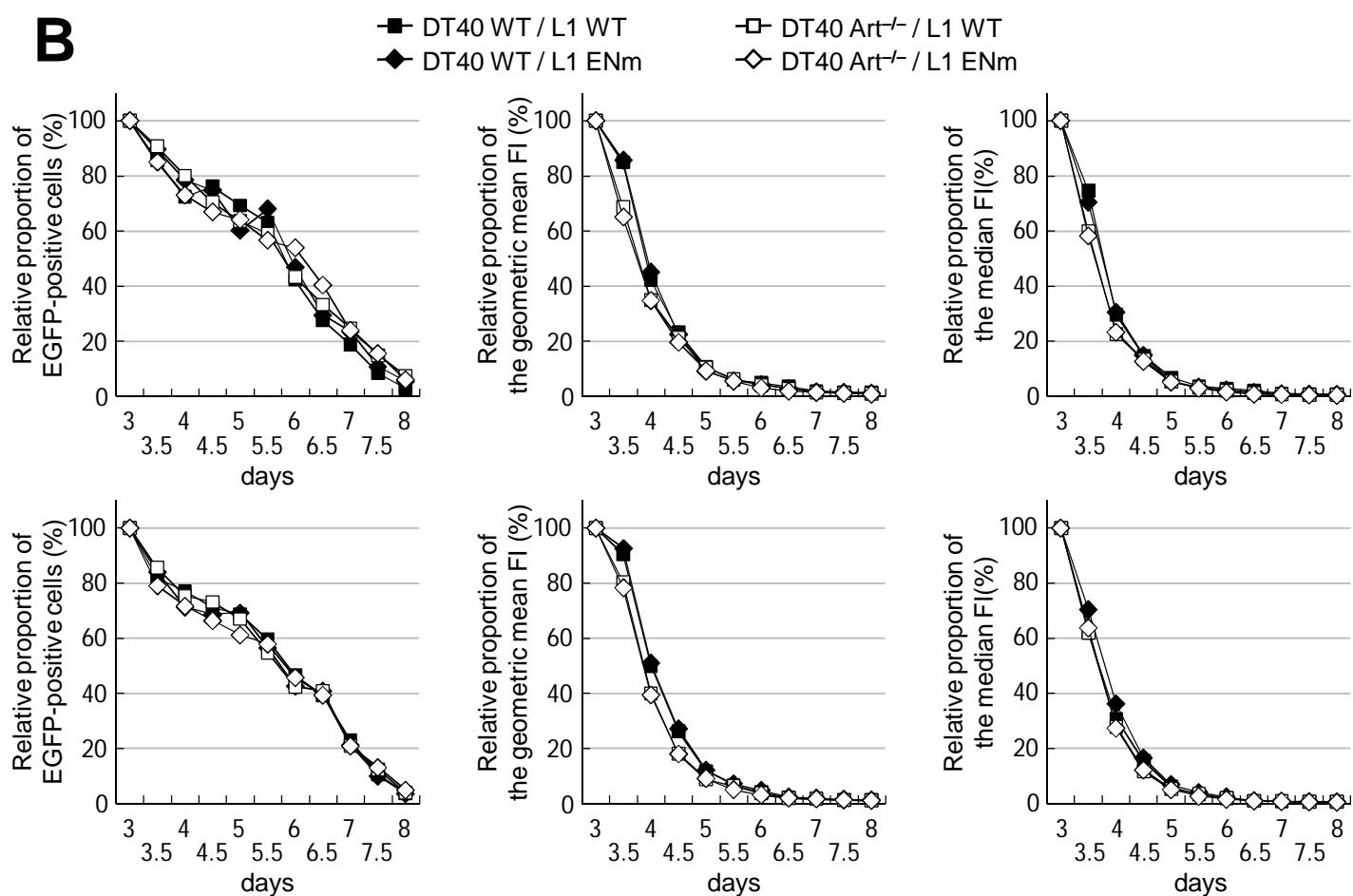

Supplement: Figure S13 — Effect of LINE expression on DT40 cell viability. DT40 cells were co-transfected with pEGFPFLAG-1 and one of the LINE expression vectors (pBZ2-5, p131.11, pJM102/L1.3, or pJM102/L1.3 H230A) by electroporation (see Tracing of EGFP-positive cells in the Materials and Methods section). After transfection, the cells were monitored for 8 days. (A) ZfL2-2 expression in DT40 cells. The relative proportion of EGFP-expressing cells (left), the geometric mean of the EGFP fluorescence intensity (FI) (middle) and the median of the EGFP FI (right) calculated using the values 3 days after electroporation as the standard are indicated (the raw data are shown in Figures S5 and S7). DT40 WT, wild-type DT40 cell line. DT40 Art−/− Artemis-deficient DT40 cell line. ZfL2-2 WT, wild-type ZfL2-2 element. ZfL2-2 ENm, endonuclease-mutated ZfL2-2 elements. Two independent experiments were performed (upper and lower panels). (B) L1 expression in DT40 cells. The relative proportion of EGFP-expressing cells (left), the geometric mean of the EGFP FI (middle) and the median of the EGFP FI (right) calculated using the values 3 days after electroporation as the standard are indicated (the raw data are shown in Figures S9 and S11). DT40 WT, wild-type DT40 cell line. DT40 Art−/−, Artemis-deficient DT40 cell line. L1 WT, wild-type L1 element. L1 ENm, endonuclease-mutated L1 elements. Two independent experiments were performed (upper and lower panels). (0.07 MB PDF) [file pgen.1000461.s013.pdf]

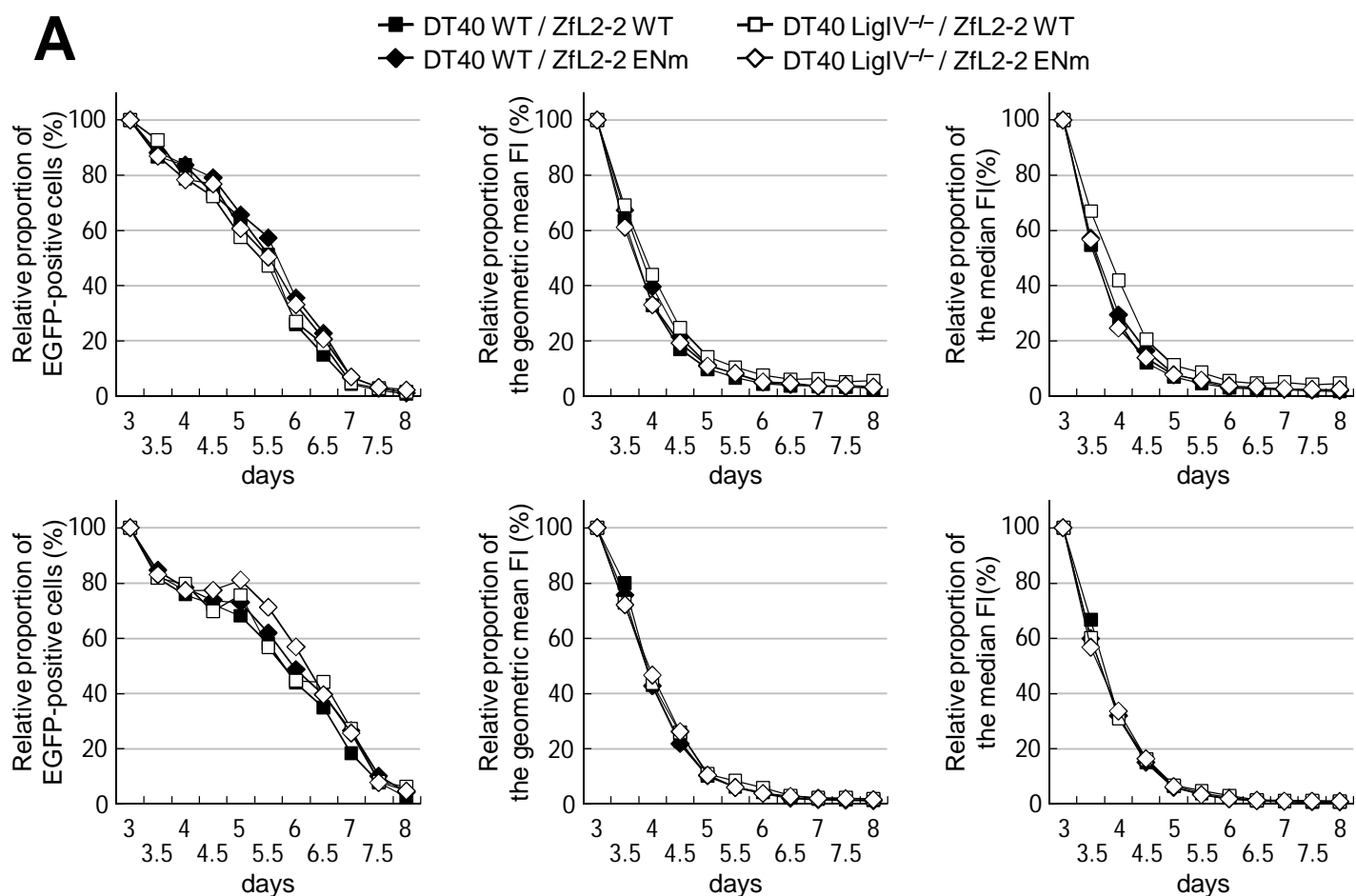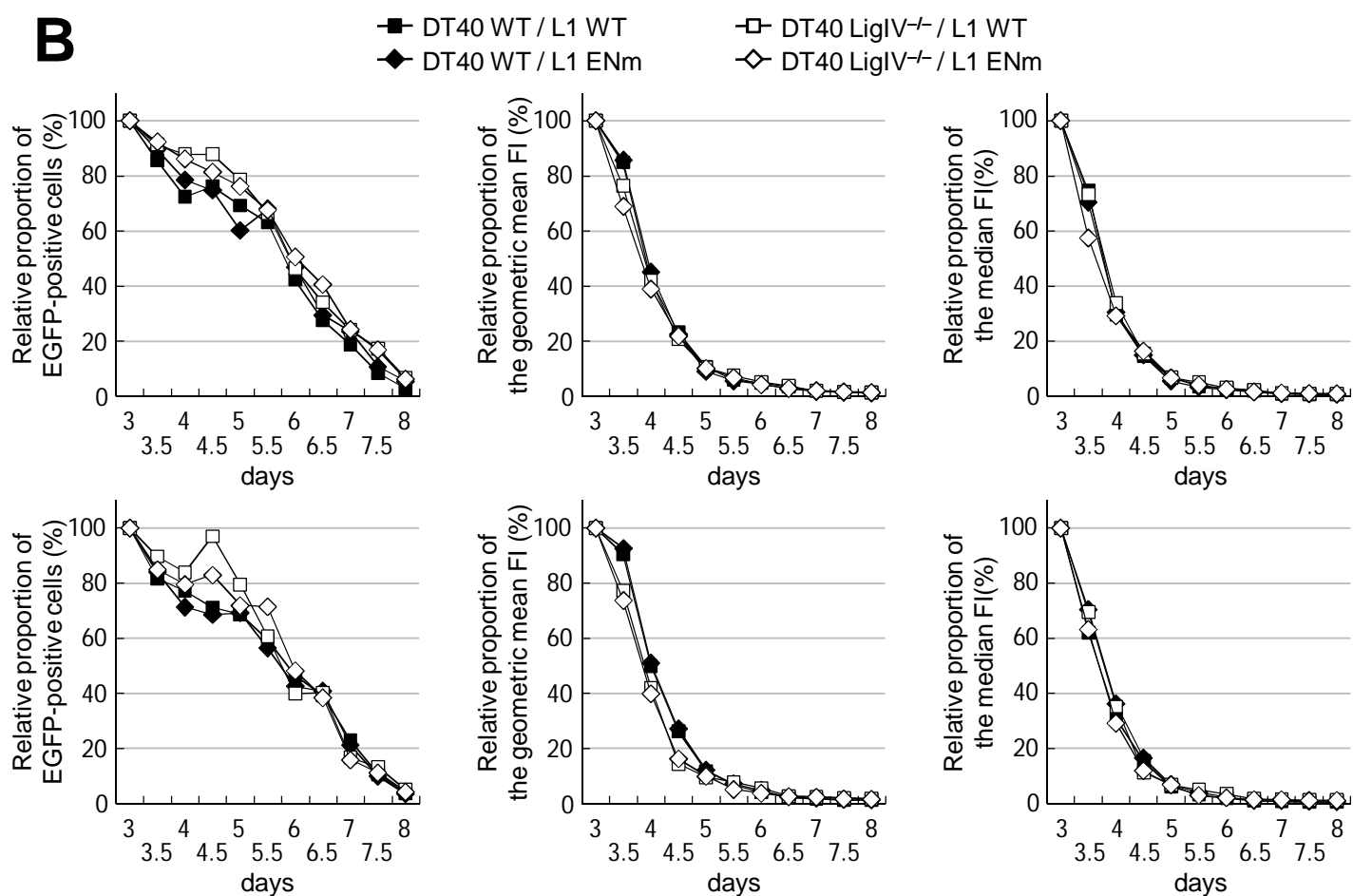

Supplement: Figure S14 — Effect of LINE expression on DT40 cell viability. DT40 cells were co-transfected with pEGFPFLAG-1 and one of the LINE expression vectors (pBZ2-5, p131.11, pJM102/L1.3, or pJM102/L1.3 H230A) by electroporation (see Tracing of EGFP-positive cells in the Materials and Methods section). After transfection, the cells were monitored for 8 days. (A) ZfL2-2 expression in DT40 cells. The relative proportion of EGFP-expressing cells (left), the geometric mean of the EGFP fluorescence intensity (FI) (middle) and the median of the EGFP FI (right) calculated using the values 3 days after electroporation as the standard are indicated (the raw data are shown in Figures S5 and S8). DT40 WT, wild-type DT40 cell line. DT40 LigIV−/−, LigaseIV-deficient DT40 cell line. ZfL2-2 WT, wild-type ZfL2-2 element. ZfL2-2 ENm, endonuclease-mutated ZfL2-2 elements. Two independent experiments were performed (upper and lower panels). (B) L1 expression in DT40 cells. The relative proportion of EGFP-expressing cells (left), the geometric mean of the EGFP FI (middle) and the median of the EGFP FI (right) calculated using the values 3 days after electroporation as the standard are indicated (the raw data are shown in Figures S9 and S12). DT40 WT, wild-type DT40 cell line. DT40 LigIV−/−, LigaseIV-deficient DT40 cell line. L1 WT, wild-type L1 element. L1 ENm, endonuclease-mutated L1 elements. Two independent experiments were performed (upper and lower panels). (0.07 MB PDF) [file pgen.1000461.s014.pdf]

A

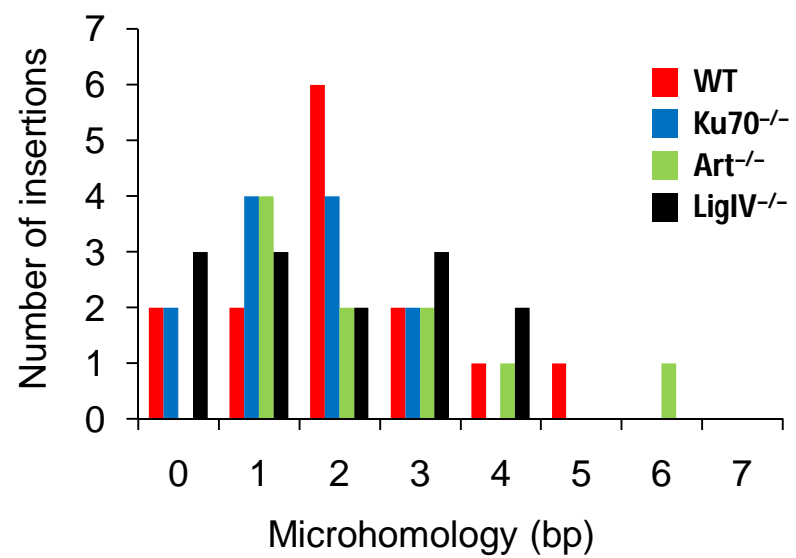

B

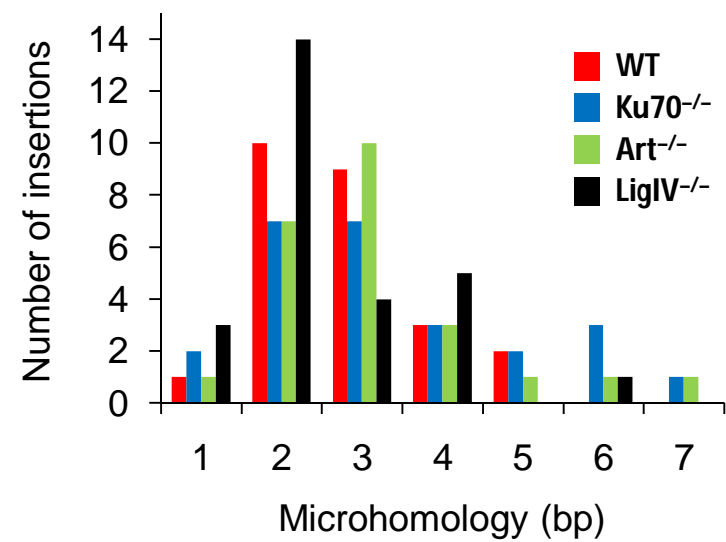

Supplement: Figure S15 — Length distributions of the 5′ and 3′ microhomologies of ZfL2-2 insertions in DT40 cells. All junctions except those with extra nucleotides are shown. (A) The length distribution of the 5′ microhomology. (B) The length distribution of the 3′ microhomology. (0.01 MB PDF) [file pgen.1000461.s015.pdf]
