# Supplementary material for: Genetic Evidence That the Non-Homologous End-Joining Repair Pathway Is Involved in LINE Retrotransposition
Source: PLoS Genet. 2009 Apr 24;5(4):e1000461. doi: 10.1371/journal.pgen.1000461 (PMC2666801; doi:10.1371/journal.pgen.1000461)
Supplement: Figure S2 — Retrotransposition assay in chicken DT40 cells. (A) Procedure for detection of LINE retrotransposition in DT40 cells. The retrotransposition detection cassette, mneoI, is inserted in the 3′ UTR of a LINE element. mneoI encodes the neomycin resistance gene (Neo), which is disrupted by an intron in the antisense orientation. The functional neomycin resistance protein is expressed only after the mneoI-marked LINE has been transcribed, spliced, and reverse transcribed into cDNA, which is then integrated into the chromosomal DNA of DT40 cells. pCMV, cytomegalovirus promoter. Pro, promoter. SVpA, SV40 polyA signal. pA, polyA signal. (B) Overview of LINE retrotransposition assay in DT40 cells. The LINE/mneoI expression vector and the EGFP expression vector are co-transfected into DT40 cells by electroporation. Three days after electroporation, the proportion of EGFP-expressing cells is measured as the transfection efficiency. At the same time, cells are plated in two kinds of soft agarose medium, one containing the antibiotic G418 and the other containing no antibiotic. Eleven days after plating, the number of colonies in the medium with no antibiotic is counted, and the plating efficiency is calculated from the colony number. The number of colonies in the G418-containing medium is also counted. The retrotransposition frequency is calculated as the number of G418 resistance (G418R) colonies per viable plated cell expressing EGFP (Materials and Methods). Small gray circles indicate EGFP-expressing cells. Small black circles indicate EGFP-expressing cells with G418R (that is, containing a LINE integrant(s) in the genomic DNA). (0.15 MB PDF) [file pgen.1000461.s002.pdf]

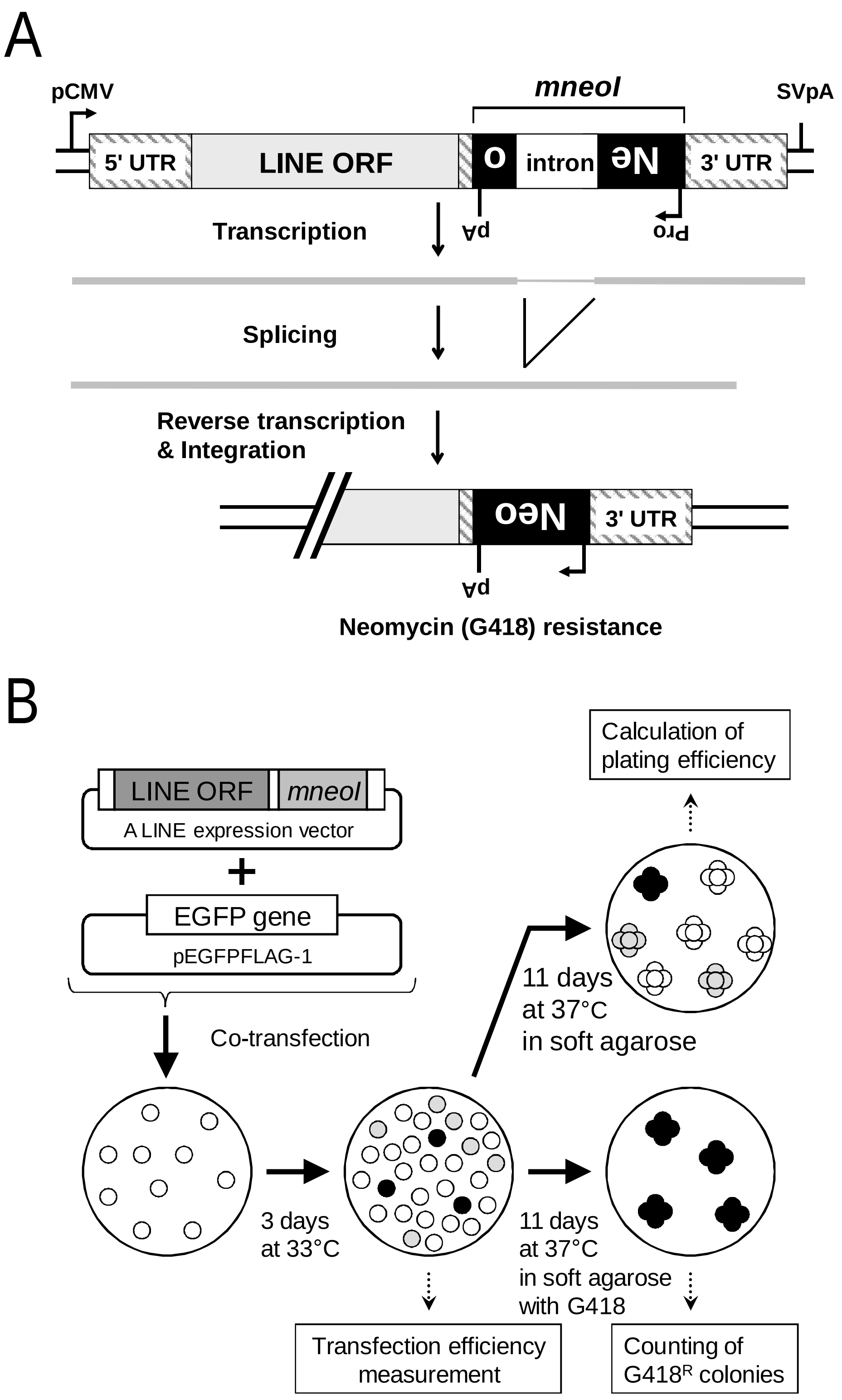
A
pCMV
mneol
SVpA
5' UTR
LINE ORF
intron
3' UTR
Transcription
Splicing
Reverse transcription
& Integration
3' UTR
Neomycin (G418) resistance
B
LINE ORF
mneol
A LINE expression vector
+
EGFP gene
pEGFPFLAG-1
Co-transfection
3 days
at 33°C
11 days
at 37°C
in soft agarose
11 days
at 37°C
in soft agarose
with G418
Calculation of
plating efficiency
Transfection efficiency
measurement
Counting of
G418R colonies
