# Supplementary material for: Genetic Evidence That the Non-Homologous End-Joining Repair Pathway Is Involved in LINE Retrotransposition
Source: PLoS Genet. 2009 Apr 24;5(4):e1000461. doi: 10.1371/journal.pgen.1000461 (PMC2666801; doi:10.1371/journal.pgen.1000461)
Supplement: Figure S3 — A possible effect of the LINE EN expression on the retrotransposition frequency (RF) of Ku70−/− cells calculated in the retrotransposition assay. Representative data from the ZfL2-2 retrotransposition assay in WT and Ku70−/− cells are shown in each panel. (A) The case in which EN expression did not influence the viability of Ku70−/− cells. (B) The case in which EN expression influenced the viability of Ku70−/− cells. Although the values shown in (A) and (B) are identical, the RF of Ku70−/− cells cannot be calculated properly (see below). Transfection efficiency - measured as the percentage of EGFP-positive cells 3 days after electroporation - was approximately 10% in Ku70−/− cells (as well as WT cells), indicating that EN expression causes a maximum of only 10% decrease in the plating efficiency if EN causes severe death of Ku70−/− cells. Thus, the plating efficiency of Ku70−/− cells was scarcely altered by EN expression. On the other hand, the number of G418-resistant colonies was markedly decreased by the severe cell death caused by EN, indicating that the proper RF value in Ku70−/− cells cannot be measured in the case of (B). However, the trace of EGFP-positive cells shown in Figure 2, S13 and S14 indicates that EN expression does not affect the cell viability of Ku70−/− cells or Artemis−/−, LigIV−/− and WT cells. We have not determined why the plating efficiency is different in each cell line, but the difference does not appear to be caused by LINE expression. Actually, the plating efficiency of untreated Ku70−/− cells (no treatment with electroporation, G418, etc.) was ∼2-fold lower than that of untreated WT cells, suggesting that the colony-forming capability of these two cell types is fundamentally different in soft agarose medium (data not shown). In addition, manipulations of the retrotransposition assay, such as the G418 selection, may differentially affect the plating efficiency of each cell line. (0.03 MB PDF) [file pgen.1000461.s003.pdf]

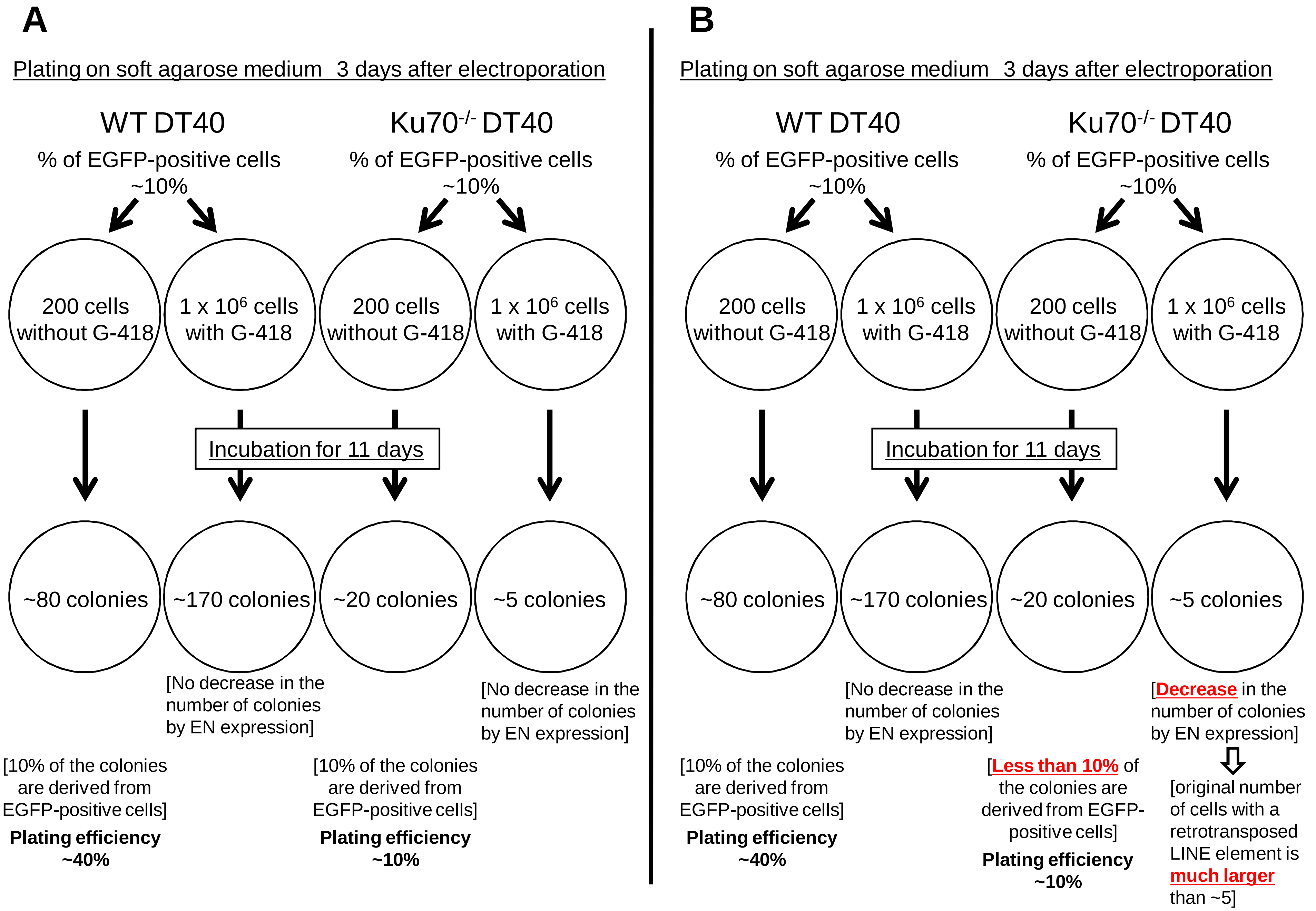
A
Plating on soft agarose medium 3 days after electroporation
WT DT40
% of EGFP-positive cells ~10%
200 cells without G-418
1 x 10^6 cells with G-418
~80 colonies
~170 colonies
[No decrease in the number of colonies by EN expression]
[10% of the colonies are derived from EGFP-positive cells]
Plating efficiency ~40%
Ku70-/- DT40
% of EGFP-positive cells ~10%
200 cells without G-418
1 x 10^6 cells with G-418
Incubation for 11 days
~20 colonies
~5 colonies
[No decrease in the number of colonies by EN expression]
[10% of the colonies are derived from EGFP-positive cells]
Plating efficiency ~10%
B
Plating on soft agarose medium 3 days after electroporation
WT DT40
% of EGFP-positive cells ~10%
200 cells without G-418
1 x 10^6 cells with G-418
~80 colonies
~170 colonies
[No decrease in the number of colonies by EN expression]
[10% of the colonies are derived from EGFP-positive cells]
Plating efficiency ~40%
Ku70-/- DT40
% of EGFP-positive cells ~10%
200 cells without G-418
1 x 10^6 cells with G-418
Incubation for 11 days
~20 colonies
~5 colonies
[Decrease in the number of colonies by EN expression]
[Less than 10% of the colonies are derived from EGFP-positive cells]
Plating efficiency ~10%
[original number of cells with a retrotransposed LINE element is much larger than ~5]
