# Supplementary material for: Genetic Evidence That the Non-Homologous End-Joining Repair Pathway Is Involved in LINE Retrotransposition
Source: PLoS Genet. 2009 Apr 24;5(4):e1000461. doi: 10.1371/journal.pgen.1000461 (PMC2666801; doi:10.1371/journal.pgen.1000461)
Supplement: Figure S4 — Flow cytometric analysis of DT40 cells electroporated with fluorescence protein expression vectors. The red fluorescence protein (DsRed-Express) and green fluorescence protein (EGFP) expression vectors were co-electroporated into DT40 cells. Flow cytometric analysis was conducted 3 days after electroporation. Ten thousand cells were counted in one measurement. Fluorescence intensities of EGFP (FL1-H) and DsRed-Express (FL2-H) are shown. A dot shows a cell expressing no fluorescence protein (black), DsRed-Express only (red), EGFP only (green) or both of the fluorescence proteins (orange). R1 is defined as the region in which cells are expressing no fluorescence protein. R2 is defined as the region in which cells are expressing DsRed-Express only. R3 is defined as the region in which cells are expressing EGFP only. R4 is defined as the region in which cells are expressing both DsRed-Express and EGFP. (A) DT40 cells electroporated with no vector DNA. (B) DT40 cells electroporated with the DsRed-Express expression vector. (C) DT40 cells electroporated with the EGFP expression vector. (D) DT40 cells electroporated with both the DsRed-Express and EGFP expression vectors. (E) The percentage of cells presented in each region (R1-4) is shown. When the two kinds of plasmid DNAs are co-electroporated into DT40 cells, most transfected (fluorescence-positive) cells (>80%) express both of the two fluorescent proteins (Figure S4E). This indicates that both plasmids are usually introduced in each DT40 cell by electroporation. In addition, the fluorescence intensities of DsRed-Express and EGFP in a doubly transfected cell are roughly proportional to each other (Figure S4D), suggesting that the amounts of each plasmid introduced into a cell are positively related. Thus, when the EGFP and LINE expression plasmids are co-transfected into DT40 cells by electroporation, the fluorescence intensity of EGFP should be roughly proportional to the expression level of the LINE protein. (0.12 MB [file pgen.1000461.s004.pdf]

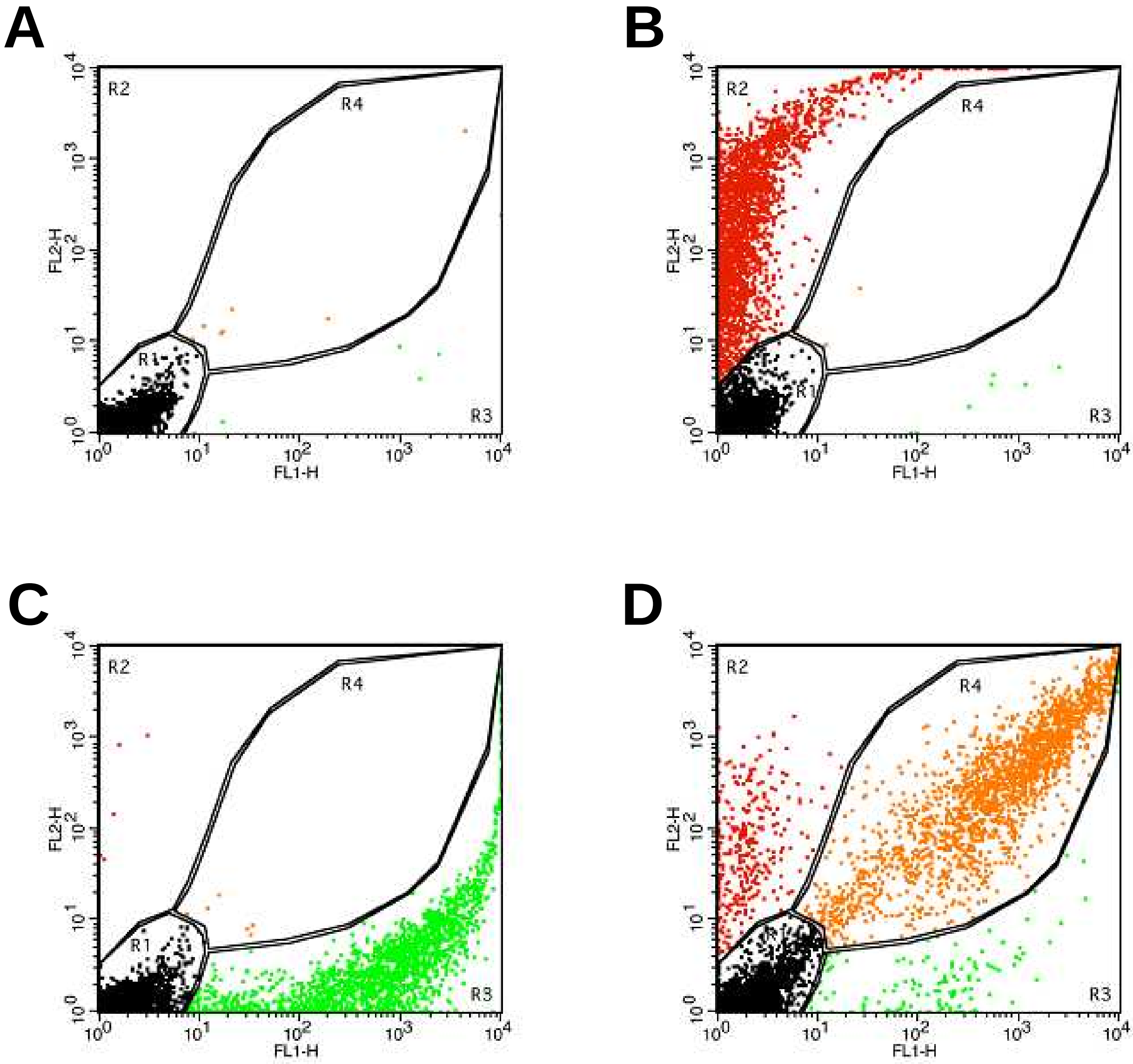

E

Region	Electroporated DNA			
	No DNA	DsRed	EGFP	DsRed & EGFP
R1 (No fluorescence)	99.85%	68.44%	75.22%	70.21%
R2 (Red)	0.00%	31.39%	0.06%	3.73%
R3 (Green)	0.07%	0.08%	24.48%	1.78%
R4 (Red & Green)	0.08%	0.03%	0.10%	23.94%
