# Supplementary material for: Genetic Evidence That the Non-Homologous End-Joining Repair Pathway Is Involved in LINE Retrotransposition
Source: PLoS Genet. 2009 Apr 24;5(4):e1000461. doi: 10.1371/journal.pgen.1000461 (PMC2666801; doi:10.1371/journal.pgen.1000461)
Supplement: Figure S6 — Flow cytometric analysis of WT and Ku70−/− DT40 cells co-electroporated with the EGFP and ZfL2-2 expression vectors. Expression of EGFP was measured from 3 to 8 days after electroporation. The histogram of the EGFP intensity (FL1-Height) is shown. The longitudinal axis shows the number of cells (Counts). The horizontal line in the histogram indicates the region defined as EGFP positive. GP, the percentage of EGFP-positive cells at each time point. Red arrowheads show the position of the geometric mean of the EGFP intensity (each value is indicated at the right of the arrowhead). Two independent experiments were conducted (A, B and C, D). (A, C) Flow cytometric data of WT and Ku70−/− DT40 cells without electroporation. (B, D) The flow cytometric data of WT and Ku70−/− DT40 cells electroporated with the EGFP expression vector and the ZfL2-2 wild-type (WT) or ZfL2-2 EN mutant (ENm) expression vector. (2.71 MB PDF) [file pgen.1000461.s006.pdf]

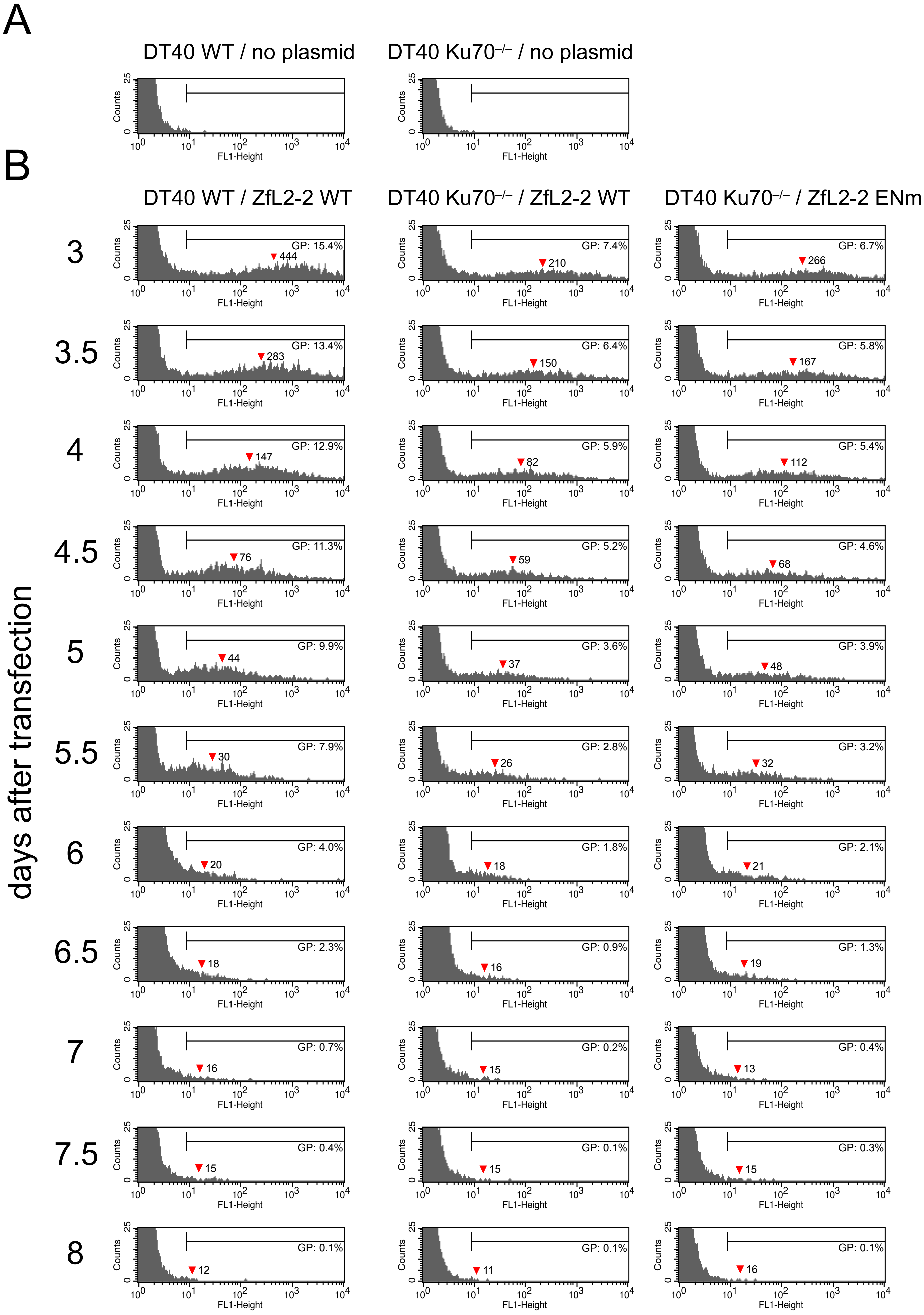
A
DT40 WT / no plasmid
DT40 Ku70−/− / no plasmid
B
DT40 WT / ZfL2-2 WT
DT40 Ku70−/− / ZfL2-2 WT
DT40 Ku70−/− / ZfL2-2 ENm
days after transfection
Counts
FL1-Height
3
GP: 15.4% 444
GP: 7.4% 210
GP: 6.7% 266
3.5
GP: 13.4% 283
GP: 6.4% 150
GP: 5.8% 167
4
GP: 12.9% 147
GP: 5.9% 82
GP: 5.4% 112
4.5
GP: 11.3% 76
GP: 5.2% 59
GP: 4.6% 68
5
GP: 9.9% 44
GP: 3.6% 37
GP: 3.9% 48
5.5
GP: 7.9% 30
GP: 2.8% 26
GP: 3.2% 32
6
GP: 4.0% 20
GP: 1.8% 18
GP: 2.1% 21
6.5
GP: 2.3% 18
GP: 0.9% 16
GP: 1.3% 19
7
GP: 0.7% 16
GP: 0.2% 15
GP: 0.4% 13
7.5
GP: 0.4% 15
GP: 0.1% 15
GP: 0.3% 15
8
GP: 0.1% 12
GP: 0.1% 11
GP: 0.1% 16

C

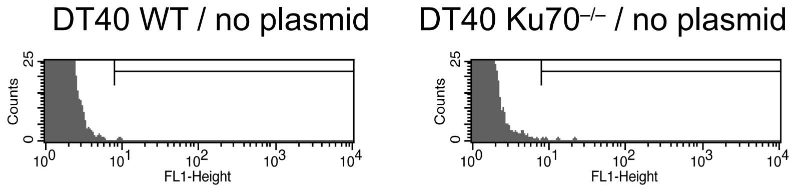
DT40 WT / no plasmid
DT40 Ku70–/– / no plasmid
Counts
0
25
FL1-Height
10^0
10^1
10^2
10^3
10^4

D

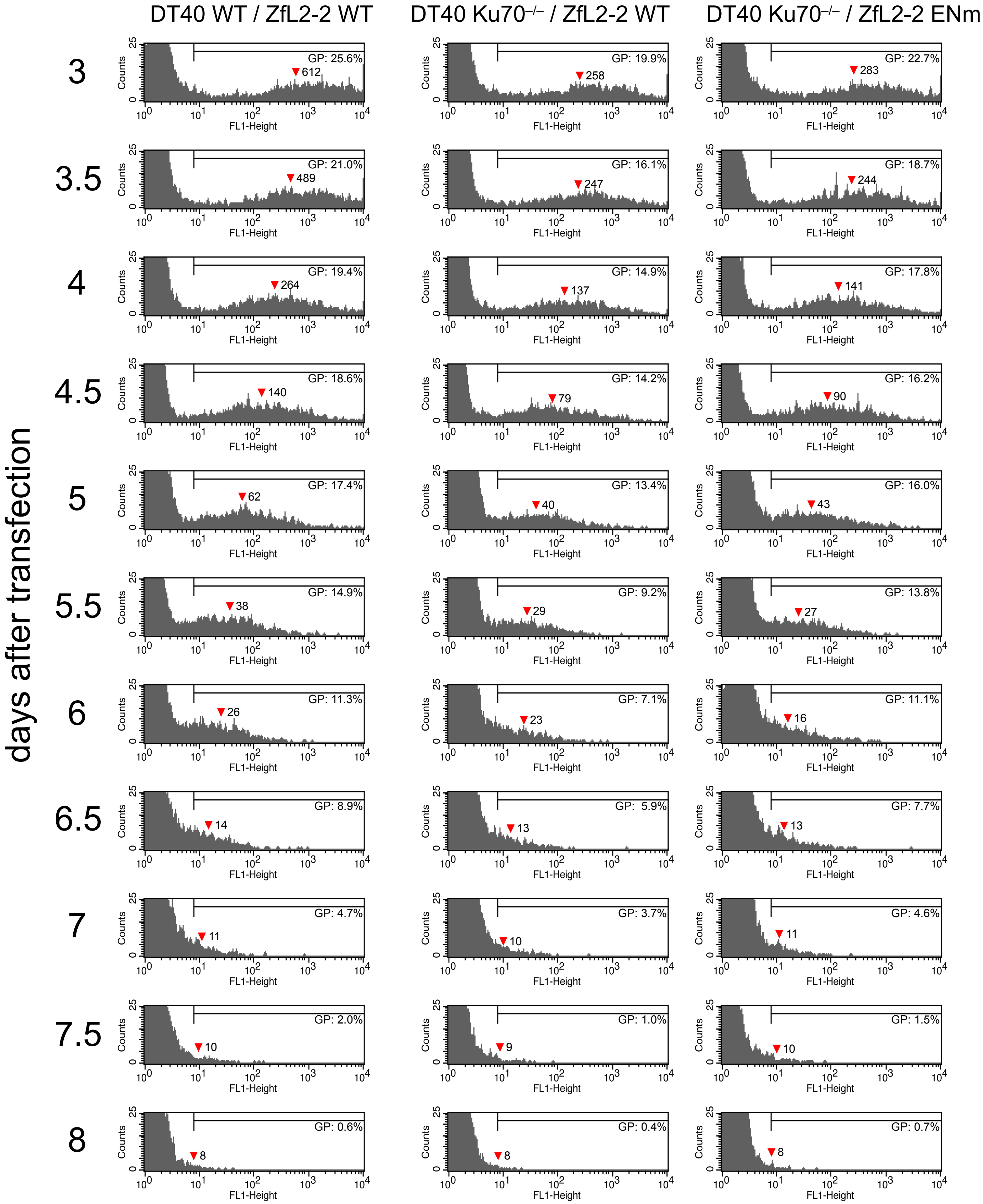
DT40 WT / ZfL2-2 WT
DT40 Ku70–/– / ZfL2-2 WT
DT40 Ku70–/– / ZfL2-2 ENm
days after transfection
Counts
FL1-Height
3
GP: 25.6%
612
GP: 19.9%
258
GP: 22.7%
283
3.5
GP: 21.0%
489
GP: 16.1%
247
GP: 18.7%
244
4
GP: 19.4%
264
GP: 14.9%
137
GP: 17.8%
141
4.5
GP: 18.6%
140
GP: 14.2%
79
GP: 16.2%
90
5
GP: 17.4%
62
GP: 13.4%
40
GP: 16.0%
43
5.5
GP: 14.9%
38
GP: 9.2%
29
GP: 13.8%
27
6
GP: 11.3%
26
GP: 7.1%
23
GP: 11.1%
16
6.5
GP: 8.9%
14
GP: 5.9%
13
GP: 7.7%
13
7
GP: 4.7%
11
GP: 3.7%
10
GP: 4.6%
11
7.5
GP: 2.0%
10
GP: 1.0%
9
GP: 1.5%
10
8
GP: 0.6%
8
GP: 0.4%
8
GP: 0.7%
8
