# Supplementary material for: Genetic Evidence That the Non-Homologous End-Joining Repair Pathway Is Involved in LINE Retrotransposition
Source: PLoS Genet. 2009 Apr 24;5(4):e1000461. doi: 10.1371/journal.pgen.1000461 (PMC2666801; doi:10.1371/journal.pgen.1000461)
Supplement: Figure S7 — Flow cytometric analysis of WT and Artemis−/− DT40 cells co-electroporated with the EGFP and ZfL2-2 expression vectors. Expression of EGFP was measured from 3 to 8 days after electroporation. The histogram of the EGFP intensity (FL1-Height) is shown. The longitudinal axis shows the number of cells (Counts). The horizontal line in the histogram indicates the region defined as EGFP positive. GP, the percentage of EGFP-positive cells at each time point. Red arrowheads show the position of the geometric mean of the EGFP intensity (each value is indicated at the right of the arrowhead). Two independent experiments were conducted (A, B and C, D). (A, C) The flow cytometric data of WT and Artemis−/− DT40 cells without electroporation. (B, D) The flow cytometric data of WT and Artemis−/− DT40 cells electroporated with the EGFP expression vector and the ZfL2-2 wild-type (WT) or ZfL2-2 EN mutant (ENm) expression vector. (2.71 MB PDF) [file pgen.1000461.s007.pdf]

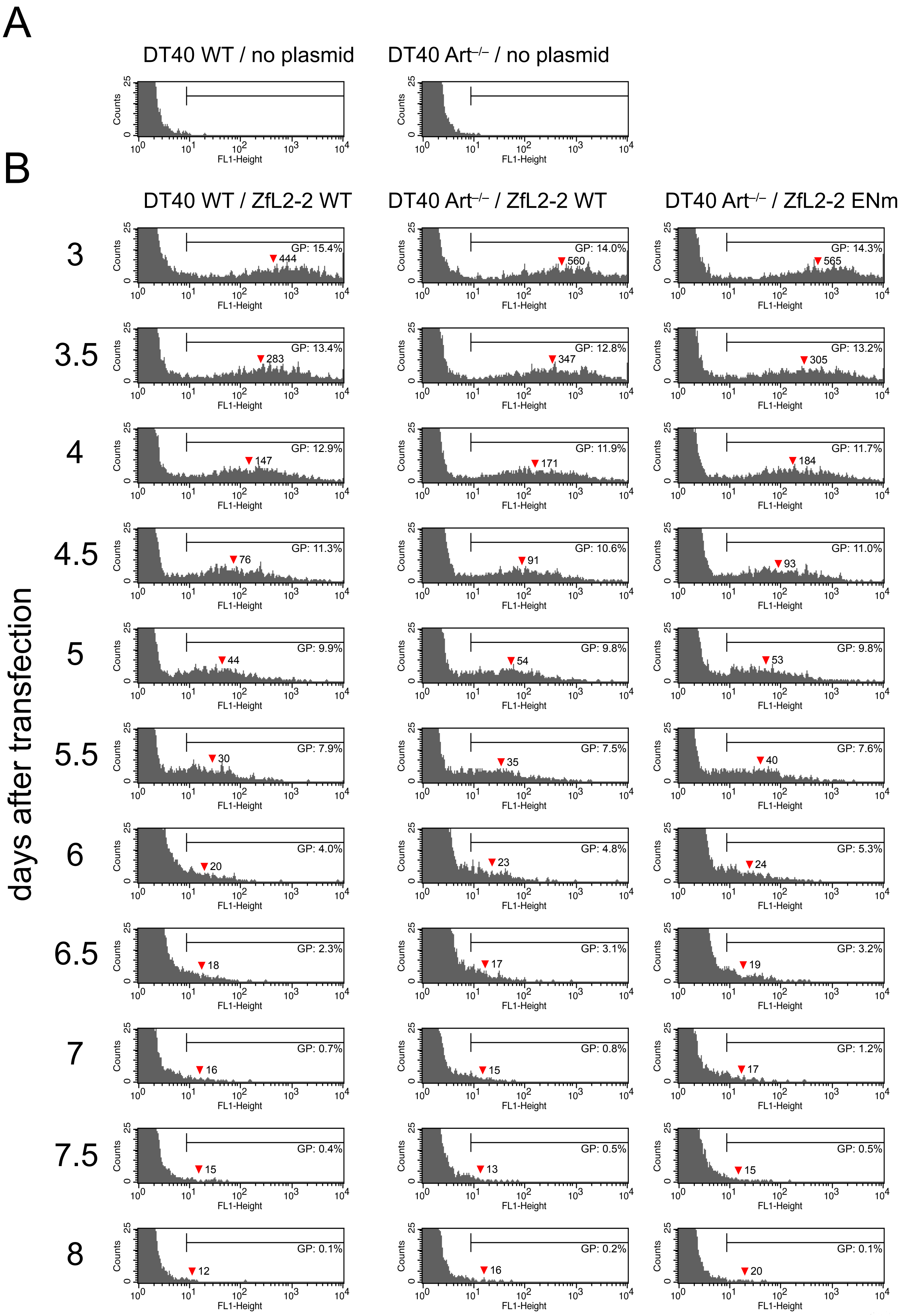
A
DT40 WT / no plasmid
DT40 Art–/– / no plasmid
B
DT40 WT / ZfL2-2 WT
DT40 Art–/– / ZfL2-2 WT
DT40 Art–/– / ZfL2-2 ENm
days after transfection
3
GP: 15.4%
444
GP: 14.0%
560
GP: 14.3%
565
3.5
GP: 13.4%
283
GP: 12.8%
347
GP: 13.2%
305
4
GP: 12.9%
147
GP: 11.9%
171
GP: 11.7%
184
4.5
GP: 11.3%
76
GP: 10.6%
91
GP: 11.0%
93
5
GP: 9.9%
44
GP: 9.8%
54
GP: 9.8%
53
5.5
GP: 7.9%
30
GP: 7.5%
35
GP: 7.6%
40
6
GP: 4.0%
20
GP: 4.8%
23
GP: 5.3%
24
6.5
GP: 2.3%
18
GP: 3.1%
17
GP: 3.2%
19
7
GP: 0.7%
16
GP: 0.8%
15
GP: 1.2%
17
7.5
GP: 0.4%
15
GP: 0.5%
13
GP: 0.5%
15
8
GP: 0.1%
12
GP: 0.2%
16
GP: 0.1%
20
Counts
FL1-Height
25
0
10^0
10^1
10^2
10^3
10^4

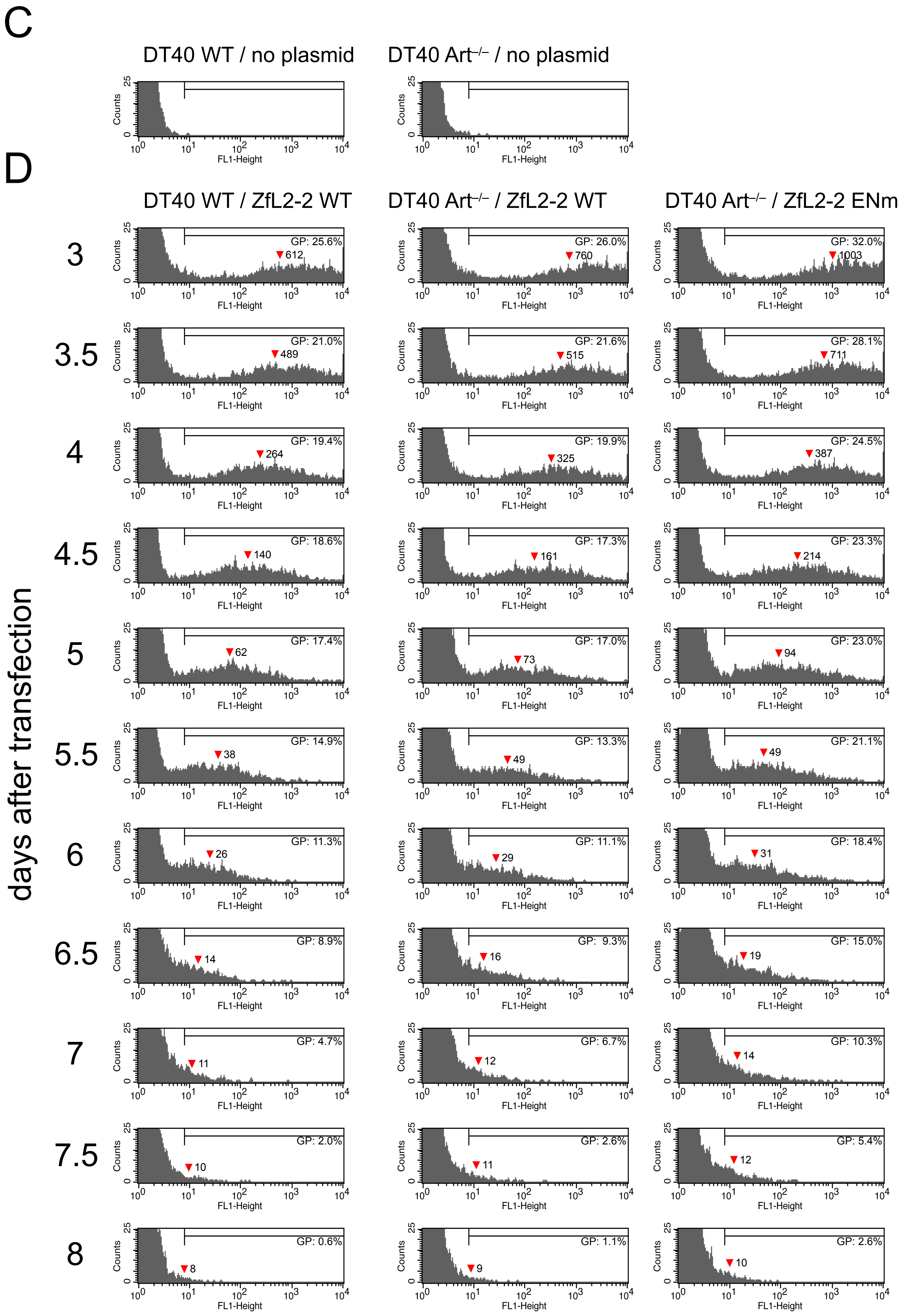

C
DT40 WT / no plasmid
DT40 Art–/– / no plasmid
D
DT40 WT / ZfL2-2 WT
DT40 Art–/– / ZfL2-2 WT
DT40 Art–/– / ZfL2-2 ENm
days after transfection
Counts
FL1-Height
3
GP: 25.6%
612
GP: 26.0%
760
GP: 32.0%
1003
3.5
GP: 21.0%
489
GP: 21.6%
515
GP: 28.1%
711
4
GP: 19.4%
264
GP: 19.9%
325
GP: 24.5%
387
4.5
GP: 18.6%
140
GP: 17.3%
161
GP: 23.3%
214
5
GP: 17.4%
62
GP: 17.0%
73
GP: 23.0%
94
5.5
GP: 14.9%
38
GP: 13.3%
49
GP: 21.1%
49
6
GP: 11.3%
26
GP: 11.1%
29
GP: 18.4%
31
6.5
GP: 8.9%
14
GP: 9.3%
16
GP: 15.0%
19
7
GP: 4.7%
11
GP: 6.7%
12
GP: 10.3%
14
7.5
GP: 2.0%
10
GP: 2.6%
11
GP: 5.4%
12
8
GP: 0.6%
8
GP: 1.1%
9
GP: 2.6%
10
