# Supplementary material for: Genetic Evidence That the Non-Homologous End-Joining Repair Pathway Is Involved in LINE Retrotransposition
Source: PLoS Genet. 2009 Apr 24;5(4):e1000461. doi: 10.1371/journal.pgen.1000461 (PMC2666801; doi:10.1371/journal.pgen.1000461)
Supplement: Figure S9 — Flow cytometric analysis of WT DT40 cells co-electroporated with the EGFP and L1 expression vectors. Expression of EGFP was measured from 3 to 8 days after electroporation. The histogram of the EGFP intensity (FL1-Height) is shown. The longitudinal axis shows the number of cells (Counts). The horizontal line in the histogram indicates the region defined as EGFP positive. GP, the percentage of EGFP-positive cells at each time point. Red arrowheads show the position of the geometric mean of the EGFP intensity (each value is indicated at the right of the arrowhead). Two independent experiments were conducted (A, B and C, D). (A, C) The flow cytometric data of WT DT40 cells without electroporation. (B, D) The flow cytometric data of WT DT40 cells electroporated with the EGFP expression vector and the L1 wild-type (WT) or L1 EN mutant (ENm) expression vector. (2.71 MB PDF) [file pgen.1000461.s009.pdf]

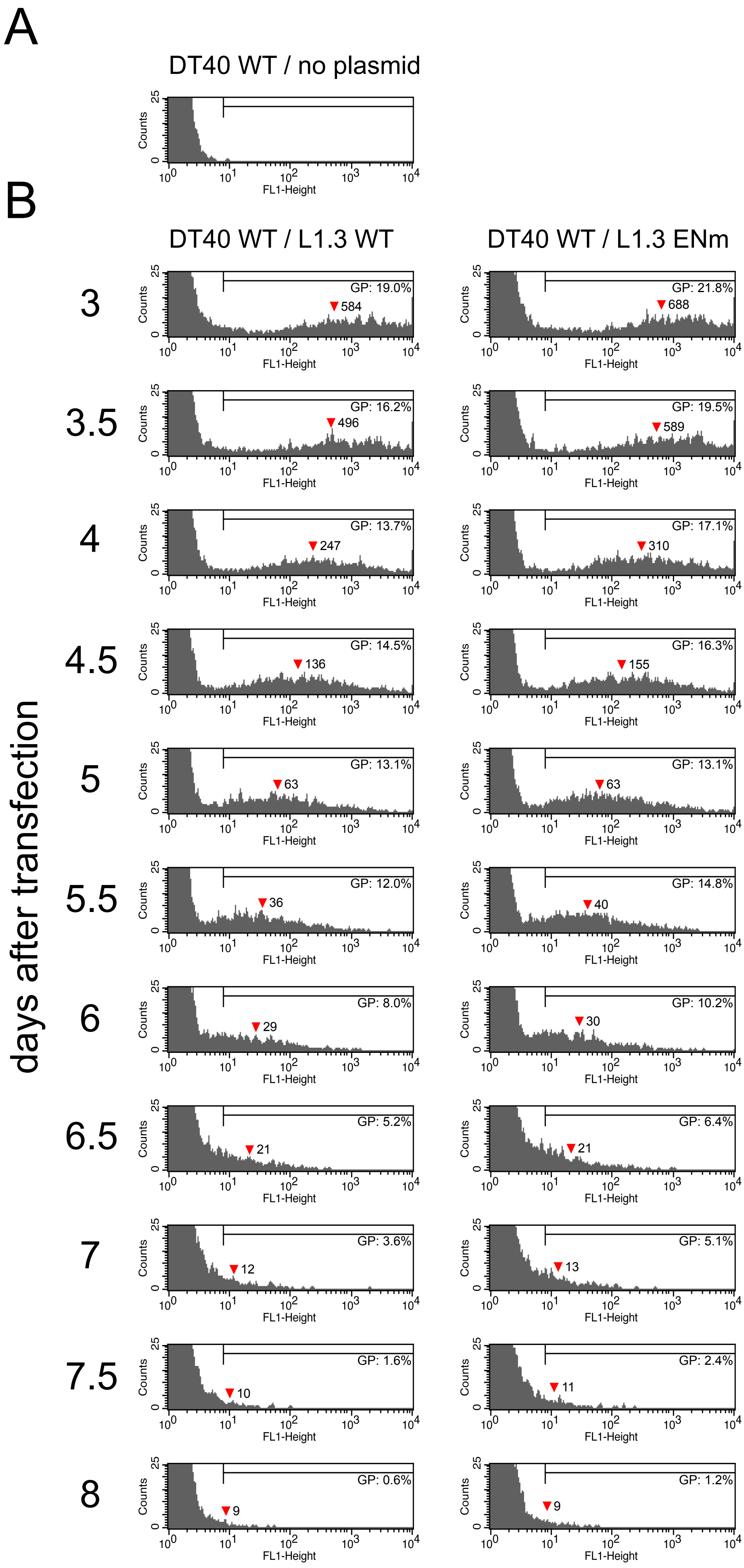

A
DT40 WT / no plasmid
B
DT40 WT / L1.3 WT
DT40 WT / L1.3 ENm
days after transfection
3
3.5
4
4.5
5
5.5
6
6.5
7
7.5
8
Counts
FL1-Height
GP: 19.0%
584
GP: 21.8%
688
GP: 16.2%
496
GP: 19.5%
589
GP: 13.7%
247
GP: 17.1%
310
GP: 14.5%
136
GP: 16.3%
155
GP: 13.1%
63
GP: 13.1%
63
GP: 12.0%
36
GP: 14.8%
40
GP: 8.0%
29
GP: 10.2%
30
GP: 5.2%
21
GP: 6.4%
21
GP: 3.6%
12
GP: 5.1%
13
GP: 1.6%
10
GP: 2.4%
11
GP: 0.6%
9
GP: 1.2%
9

C

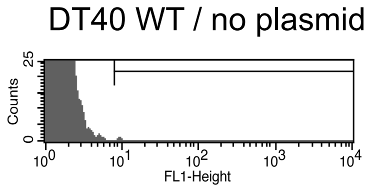

DT40 WT / no plasmid
Counts
FL1-Height

D

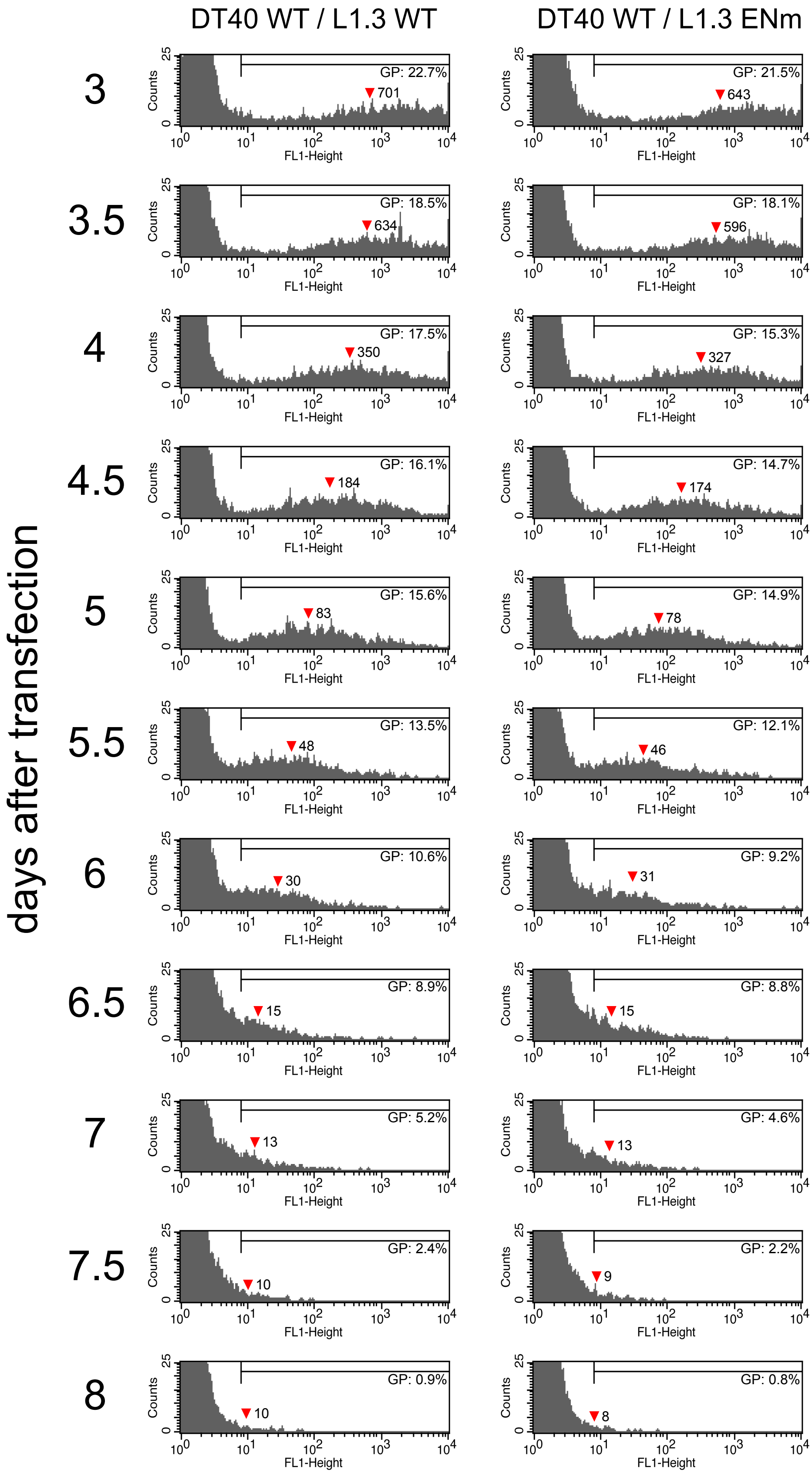

DT40 WT / L1.3 WT
DT40 WT / L1.3 ENm
days after transfection
Counts
FL1-Height
3
GP: 22.7%
701
GP: 21.5%
643
3.5
GP: 18.5%
634
GP: 18.1%
596
4
GP: 17.5%
350
GP: 15.3%
327
4.5
GP: 16.1%
184
GP: 14.7%
174
5
GP: 15.6%
83
GP: 14.9%
78
5.5
GP: 13.5%
48
GP: 12.1%
46
6
GP: 10.6%
30
GP: 9.2%
31
6.5
GP: 8.9%
15
GP: 8.8%
15
7
GP: 5.2%
13
GP: 4.6%
13
7.5
GP: 2.4%
10
GP: 2.2%
9
8
GP: 0.9%
10
GP: 0.8%
8
