# Supplementary material for: Genetic Evidence That the Non-Homologous End-Joining Repair Pathway Is Involved in LINE Retrotransposition
Source: PLoS Genet. 2009 Apr 24;5(4):e1000461. doi: 10.1371/journal.pgen.1000461 (PMC2666801; doi:10.1371/journal.pgen.1000461)
Supplement: Figure S10 — Flow cytometric analysis of WT and Ku70−/− DT40 cells co-electroporated with the EGFP and L1 expression vectors. Expression of EGFP was measured from 3 to 8 days after electroporation. The histogram of the EGFP intensity (FL1-Height) is shown. The longitudinal axis shows the number of cells (Counts). The horizontal line in the histogram indicates the region defined as EGFP positive. GP, the percentage of EGFP-positive cells at each time point. Red arrowheads show the position of the geometric mean of the EGFP intensity (each value is indicated at the right of the arrowhead). Two independent experiments were conducted (A, B and C, D). (A, C) The flow cytometric data of WT and Ku70−/− DT40 cells without electroporation. (B, D) The flow cytometric data of WT and Ku70−/− DT40 cells electroporated with the EGFP expression vector and the L1 wild-type (WT) or L1 EN mutant (ENm) expression vector. (2.72 MB PDF) [file pgen.1000461.s010.pdf]

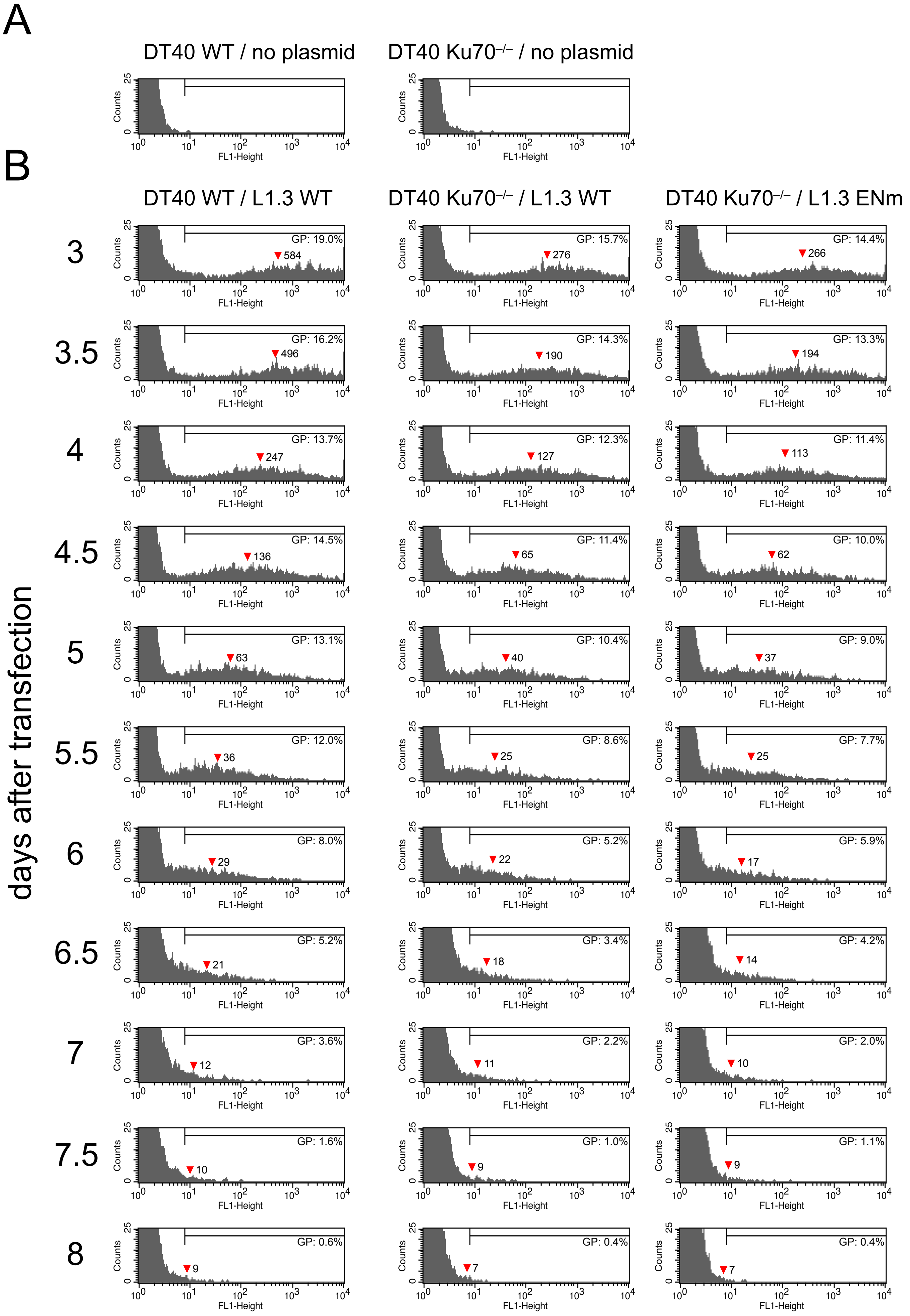
A
DT40 WT / no plasmid
DT40 Ku70−/− / no plasmid
B
DT40 WT / L1.3 WT
DT40 Ku70−/− / L1.3 WT
DT40 Ku70−/− / L1.3 ENm
days after transfection
3
GP: 19.0% 584
GP: 15.7% 276
GP: 14.4% 266
3.5
GP: 16.2% 496
GP: 14.3% 190
GP: 13.3% 194
4
GP: 13.7% 247
GP: 12.3% 127
GP: 11.4% 113
4.5
GP: 14.5% 136
GP: 11.4% 65
GP: 10.0% 62
5
GP: 13.1% 63
GP: 10.4% 40
GP: 9.0% 37
5.5
GP: 12.0% 36
GP: 8.6% 25
GP: 7.7% 25
6
GP: 8.0% 29
GP: 5.2% 22
GP: 5.9% 17
6.5
GP: 5.2% 21
GP: 3.4% 18
GP: 4.2% 14
7
GP: 3.6% 12
GP: 2.2% 11
GP: 2.0% 10
7.5
GP: 1.6% 10
GP: 1.0% 9
GP: 1.1% 9
8
GP: 0.6% 9
GP: 0.4% 7
GP: 0.4% 7
Counts
FL1-Height

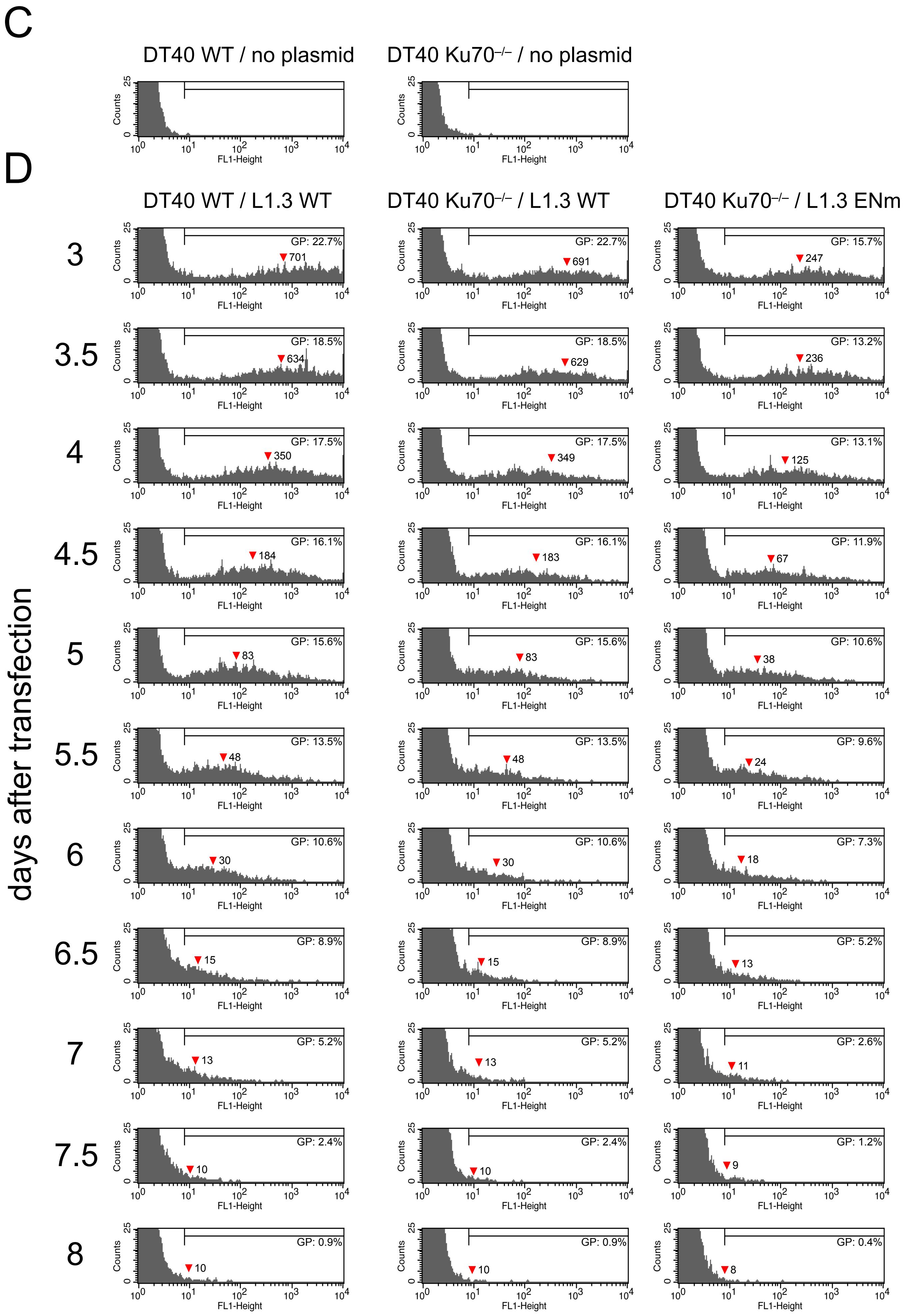
C
DT40 WT / no plasmid
DT40 Ku70−/− / no plasmid
D
DT40 WT / L1.3 WT
DT40 Ku70−/− / L1.3 WT
DT40 Ku70−/− / L1.3 ENm
days after transfection
3
3.5
4
4.5
5
5.5
6
6.5
7
7.5
8
Counts
FL1-Height
GP: 22.7% 701
GP: 22.7% 691
GP: 15.7% 247
GP: 18.5% 634
GP: 18.5% 629
GP: 13.2% 236
GP: 17.5% 350
GP: 17.5% 349
GP: 13.1% 125
GP: 16.1% 184
GP: 16.1% 183
GP: 11.9% 67
GP: 15.6% 83
GP: 15.6% 83
GP: 10.6% 38
GP: 13.5% 48
GP: 13.5% 48
GP: 9.6% 24
GP: 10.6% 30
GP: 10.6% 30
GP: 7.3% 18
GP: 8.9% 15
GP: 8.9% 15
GP: 5.2% 13
GP: 5.2% 13
GP: 5.2% 13
GP: 2.6% 11
GP: 2.4% 10
GP: 2.4% 10
GP: 1.2% 9
GP: 0.9% 10
GP: 0.9% 10
GP: 0.4% 8
