# Supplementary material for: Genetic Evidence That the Non-Homologous End-Joining Repair Pathway Is Involved in LINE Retrotransposition
Source: PLoS Genet. 2009 Apr 24;5(4):e1000461. doi: 10.1371/journal.pgen.1000461 (PMC2666801; doi:10.1371/journal.pgen.1000461)
Supplement: Figure S12 — Flow cytometric analysis of WT and LigaseIV−/− DT40 cells co-electroporated with the EGFP and L1 expression vectors. Expression of EGFP was measured from 3 to 8 days after electroporation. The histogram of the EGFP intensity (FL1-Height) is shown. The longitudinal axis shows the number of cells (Counts). The horizontal line in the histogram indicates the region defined as EGFP positive. GP, the percentage of EGFP-positive cells at each time point. Red arrowheads show the position of the geometric mean of the EGFP intensity (each value is indicated at the right of the arrowhead). Two independent experiments were conducted (A, B and C, D). (A, C) The flow cytometric data of WT and LigaseIV−/− DT40 cells without electroporation. (B, D) The flow cytometric data of WT and LigaseIV−/− DT40 cells electroporated with the EGFP expression vector and the L1 wild-type (WT) or L1 EN mutant (ENm) expression vector. (2.72 MB PDF) [file pgen.1000461.s012.pdf]

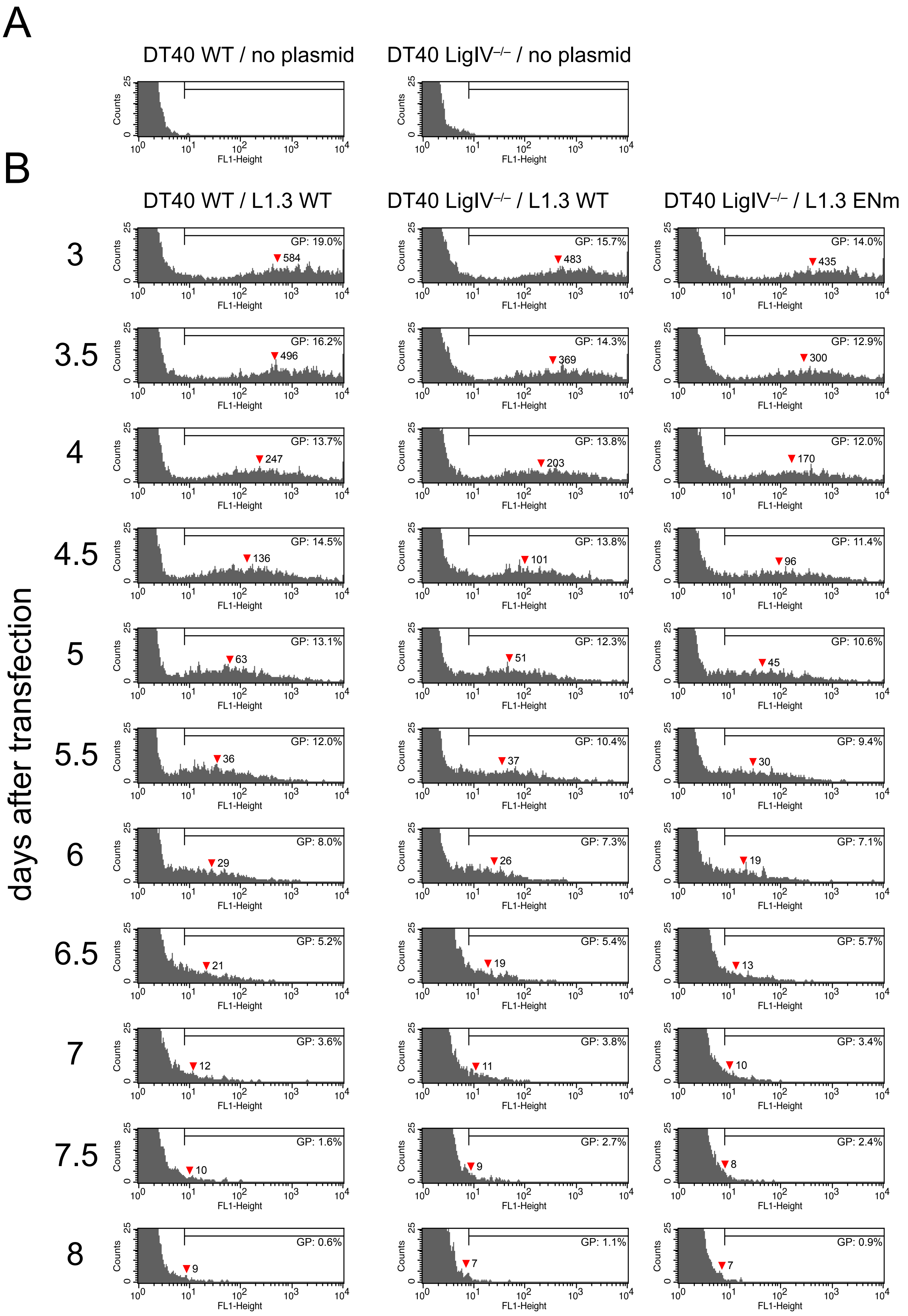
A
DT40 WT / no plasmid
DT40 LigIV–/– / no plasmid
B
DT40 WT / L1.3 WT
DT40 LigIV–/– / L1.3 WT
DT40 LigIV–/– / L1.3 ENm
days after transfection
3
3.5
4
4.5
5
5.5
6
6.5
7
7.5
8
Counts
FL1-Height
GP: 19.0%
584
GP: 15.7%
483
GP: 14.0%
435
GP: 16.2%
496
GP: 14.3%
369
GP: 12.9%
300
GP: 13.7%
247
GP: 13.8%
203
GP: 12.0%
170
GP: 14.5%
136
GP: 13.8%
101
GP: 11.4%
96
GP: 13.1%
63
GP: 12.3%
51
GP: 10.6%
45
GP: 12.0%
36
GP: 10.4%
37
GP: 9.4%
30
GP: 8.0%
29
GP: 7.3%
26
GP: 7.1%
19
GP: 5.2%
21
GP: 5.4%
19
GP: 5.7%
13
GP: 3.6%
12
GP: 3.8%
11
GP: 3.4%
10
GP: 1.6%
10
GP: 2.7%
9
GP: 2.4%
8
GP: 0.6%
9
GP: 1.1%
7
GP: 0.9%
7

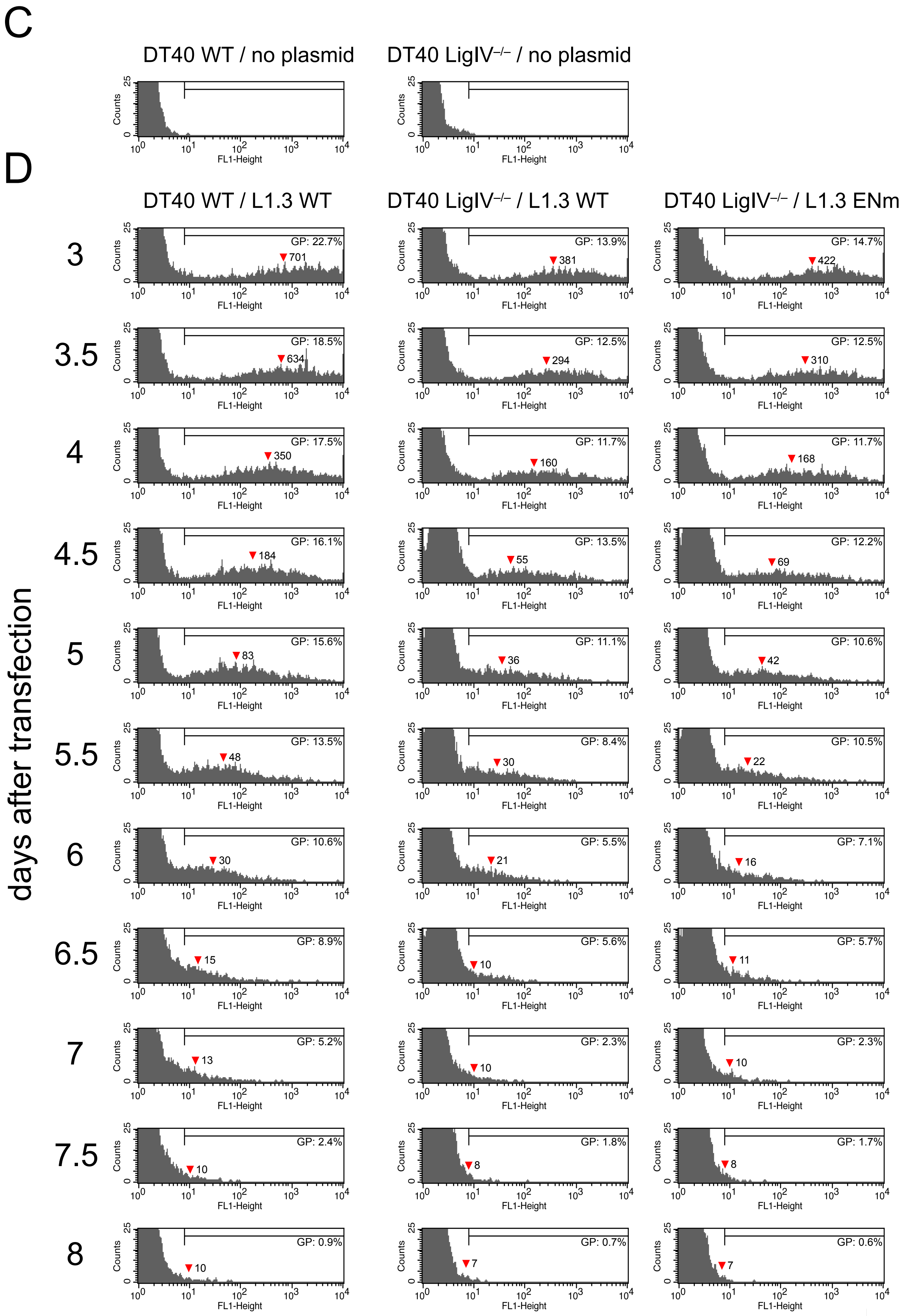

C
DT40 WT / no plasmid
DT40 LigIV–/– / no plasmid
D
DT40 WT / L1.3 WT
DT40 LigIV–/– / L1.3 WT
DT40 LigIV–/– / L1.3 ENm
days after transfection
Counts
FL1-Height
3
GP: 22.7%
701
GP: 13.9%
381
GP: 14.7%
422
3.5
GP: 18.5%
634
GP: 12.5%
294
GP: 12.5%
310
4
GP: 17.5%
350
GP: 11.7%
160
GP: 11.7%
168
4.5
GP: 16.1%
184
GP: 13.5%
55
GP: 12.2%
69
5
GP: 15.6%
83
GP: 11.1%
36
GP: 10.6%
42
5.5
GP: 13.5%
48
GP: 8.4%
30
GP: 10.5%
22
6
GP: 10.6%
30
GP: 5.5%
21
GP: 7.1%
16
6.5
GP: 8.9%
15
GP: 5.6%
10
GP: 5.7%
11
7
GP: 5.2%
13
GP: 2.3%
10
GP: 2.3%
10
7.5
GP: 2.4%
10
GP: 1.8%
8
GP: 1.7%
8
8
GP: 0.9%
10
GP: 0.7%
7
GP: 0.6%
7
