# Supplementary material for: Genetic Evidence That the Non-Homologous End-Joining Repair Pathway Is Involved in LINE Retrotransposition
Source: PLoS Genet. 2009 Apr 24;5(4):e1000461. doi: 10.1371/journal.pgen.1000461 (PMC2666801; doi:10.1371/journal.pgen.1000461)
Supplement: Table S1 — ZfL2-2 retrotransposition in DT40 cells. (0.05 MB DOC) [file pgen.1000461.s016.doc]

Table S1: ZfL2-2 retrotransposition in DT40 cells

| DT40 cell line | ZfL2-2 status | n^a^ | Transfection efficiency^b^  (%) | Geometric mean of EGFP FI^c^ | Median of EGFP FI^d^ | Number of G418^R^ colonies per dish^e^ | Plating efficiency^f^ (%) | Retrotransposition frequency (× 10^–3^)  (Mean ± SD) | Percent of WT^g^ |
| --- | --- | --- | --- | --- | --- | --- | --- | --- | --- |
| Wild type | WT | 8 | 11 ± 4 | 751 ± 103 | 1071 ± 152 | 173 ± 40 | 44 ± 14 | 4.9 ± 0.8 | 100% |
|  | EN^–^ | 2 | 6 ± 3 | 351 ± 171 | 387 ± 178 | 0 | 40 ± 15 | < 0.1 | - |
| Ku70^–/–^ | WT | 7 | 12 ± 3 | 447 ± 78 | 532 ± 121 | 4 ± 2 | 8 ± 3 | 0.6 ± 0.4 | 12% |
|  | EN^–^ | 2 | 6 ± 2 | 246 ± 27 | 246 ± 43 | 0 | 5 ± 0.3 | < 0.5 | - |
| Artemis^–/–^ | WT | 7 | 18 ± 3 | 1298 ± 149 | 1676 ± 175 | 41 ± 22 | 16 ± 8 | 2.0 ± 1.3 | 41% |
|  | EN^–^ | 2 | 9 ± 7 | 433 ± 135 | 444 ± 165 | 0 | 27 ± 13 | < 0.2 | - |
| LigIV^–/–^ | WT | 7 | 10 ± 4 | 657 ± 72 | 833 ± 86 | 16 ± 11 | 19 ± 15 | 1.2 ± 0.8 | 24% |
|  | EN^–^ | 2 | 10 ± 4 | 300 ± 43 | 303 ± 37 | 0 | 21 ± 6 | < 0.1 | - |
| Rad18^–/–^ | WT | 5 | 12 ± 5 | 825 ± 257 | 1095 ± 382 | 438 ± 312 | 65 ± 20 | 6.2 ± 0.9 | 128% |
|  | EN^–^ | 2 | 9 ± 6 | 471 ± 109 | 491 ± 129 | 0 | 29 ± 9 | < 0.1 | - |
| SHIP1^–/–^ | WT | 6 | 11 ± 4 | 618 ± 76 | 865 ± 99 | 139 ± 83 | 28 ± 13 | 5.1 ± 1.1 | 106% |
|  | EN^–^ | 2 | 13 ± 2 | 367 ± 80 | 389 ± 102 | 0 | 46 ± 30 | < 0.1 | - |

^a^n indicates the number of independent experiments. ^b^The transfection efficiency was calculated as the percentage of the EGFP-positive cells 3 days after electroporation. ^c^Mean ± standard deviation (SD) of the geometric mean of the EGFP fluorescence intensity (FI) 3 days after electroporation. ^d^Mean ± SD of the median of the EGFP FI 3 days after electroporation. ^e^Mean ± SD of G418-resistant colonies per dish in which ~1 × 10^6^ electroporated DT40 cells were plated (see Materials and Methods). ^f^Plating efficiency was calculated as the percentage of the number of colonies formed in soft-agarose medium containing no antibiotic relative to the 200 DT40 cells plated (see Materials and Methods). Mean ± SD of the plating efficiency is shown. ^g^The percentage of ZfL2-2 retrotransposition was determined with respect to the retrotransposition frequency of wild-type ZfL2-2 in wild-type DT40. WT, wild type. EN^–^, endonuclease mutant.
