# Supplementary material for: Genetic Evidence That the Non-Homologous End-Joining Repair Pathway Is Involved in LINE Retrotransposition
Source: PLoS Genet. 2009 Apr 24;5(4):e1000461. doi: 10.1371/journal.pgen.1000461 (PMC2666801; doi:10.1371/journal.pgen.1000461)
Supplement: Table S2 — L1 retrotransposition in DT40 cells. (0.05 MB DOC) [file pgen.1000461.s017.doc]

Table S2: L1 retrotransposition in DT40 cells

| DT40 cell line | L1 status | n^a^ | Transfection efficiency^b^ (%) | Geometric mean of EGFP FI^c^ | Median of EGFP FI^d^ | Number of G418^R^ colonies per dish^e^ | Plating efficiency^f^ (%) | Retrotransposition frequency (× 10^-3^)  (Mean ± SD) | Percent of WT^g^ |
| --- | --- | --- | --- | --- | --- | --- | --- | --- | --- |
| Wild type | WT | 14 | 7 ± 5 | 602 ± 217 | 819 ± 434 | 258 ± 145 | 46 ± 12 | 12.0 ± 3.3 | 100% |
|  | EN^–^ | 2 | 3 ± 1 | 425 ± 5 | 434 ± 36 | 0 | 49 ± 21 | < 0.2 | - |
| Ku70^–/–^ | WT | 8 | 4 ± 2 | 328 ± 90 | 348 ± 126 | 16 ± 12 | 15 ± 12 | 5.3 ± 2.4 | 44% |
|  | EN^–^ | 2 | 3 ± 2 | 321 ± 33 | 365 ± 76 | 0 | 8 ± 3 | < 1.1 | - |
| Artemis^–/–^ | WT | 11 | 7 ± 4 | 840 ± 295 | 1022 ± 452 | 63 ± 55 | 19 ± 10 | 5.9 ± 2.6 | 49% |
|  | EN^–^ | 2 | 5 ± 2 | 498 ± 63 | 482 ± 75 | 0 | 14 ± 3 | < 0.3 | - |
| LigIV^–/–^ | WT | 11 | 2 ± 1 | 387 ± 103 | 440 ± 159 | 28 ± 12 | 31 ± 19 | 8.0 ± 2.9 | 67% |
|  | EN^–^ | 2 | 2 ± 1 | 344 ± 28 | 322 ± 30 | 0 | 26 ± 5 | < 0.6 | - |
| Rad18^–/–^ | WT | 6 | 10 ± 5 | 672 ± 161 | 948 ± 289 | 358 ± 134 | 42 ± 20 | 16.6 ± 5.1 | 139% |
|  | EN^–^ | 2 | 6 ± 1 | 415 ± 125 | 423 ± 134 | 0 | 28 ± 10 | < 0.2 | - |
| SHIP1^–/–^ | WT | 7 | 6 ± 3 | 573 ± 209 | 818 ±354 | 91 ± 36 | 21 ± 5 | 12.1 ± 5.2 | 101% |
|  | EN^–^ | 2 | 3 ± 1 | 394 ± 5 | 482 ± 18 | 0 | 45 ± 7 | < 0.3 | - |

^a^n indicates the number of independent experiments. ^b^The transfection efficiency was calculated as the percentage of the EGFP-positive cells 3 days after electroporation. ^c^Mean ± standard deviation (SD) of the geometric mean of the EGFP fluorescence intensity (FI) 3 days after electroporation. ^d^Mean ± SD of the median of the EGFP FI 3 days after electroporation. ^e^Mean ± SD of G418-resistant colonies per dish in which ~1 × 10^6^ electroporated DT40 cells were plated (see Materials and Methods). ^f^Plating efficiency was calculated as the percentage of the number of colonies formed in soft agarose medium containing no antibiotic relative to the 200 DT40 cells plated (see Materials and Methods). Mean ± SD of the plating efficiency is shown. ^g^The percentage of L1 retrotransposition was determined with respect to the retrotransposition frequency of wild-type L1 in wild-type DT40. WT, wild type. EN^–^, endonuclease mutant.
