# Supplementary material for: Genetic Evidence That the Non-Homologous End-Joining Repair Pathway Is Involved in LINE Retrotransposition
Source: PLoS Genet. 2009 Apr 24;5(4):e1000461. doi: 10.1371/journal.pgen.1000461 (PMC2666801; doi:10.1371/journal.pgen.1000461)
Supplement: Table S3 — Ku70 complementation assay with ZfL2-2 in DT40 cells. (0.04 MB DOC) [file pgen.1000461.s018.doc]

Table S3: Ku70 complementation assay with ZfL2-2 in DT40 cells

| DT40 cell line | ZfL2-2 status | n^a^ | Transfection efficiency^b^ (%) | Geometric mean of EGFP FI^c^ | Median of EGFP FI^d^ | Number of G418^R^ colonies per dish^e^ | Plating efficiency^f^ (%) | Retrotransposition frequency (× 10^-3^)  (Mean ± SD) | Percent of WT^g^ |
| --- | --- | --- | --- | --- | --- | --- | --- | --- | --- |
| Wild type | pAneo | 6 | 6 ± 1 | 335 ± 80 | 351 ± 101 | 78 ± 28 | 64 ± 23 | 2.9 ± 0.4 | 100% |
|  | pKu70 | 6 | 8 ± 2 | 341 ± 62 | 343 ± 65 | 131 ± 44 | 62 ± 13 | 3.2 ± 0.6 | 110% |
| Ku70^–/–^ | pAneo | 6 | 5 ± 2 | 259 ± 29 | 269 ±42 | 3 ± 2 | 14 ± 8 | 0.5 ± 0.2^f^ | 18%^h^ |
|  | pKu70 | 6 | 5 ± 2 | 286 ± 35 | 291 ±38 | 18 ± 9 | 16 ± 7 | 3.2 ± 0.9 | 111% |
| LigIV^–/–^ | pAneo | 6 | 5 ± 1 | 262 ± 19 | 259 ± 14 | 13 ± 4 | 32 ± 14 | 1.1 ± 0.3 | 38% |
|  | pKu70 | 6 | 5 ± 2 | 252 ± 37 | 256 ±54 | 12 ± 9 | 30 ± 15 | 1.0 ± 0.3 | 34% |

^a^n indicates the number of independent experiments. ^b^The transfection efficiency was calculated as the percentage of the EGFP-positive cells 3 days after electroporation. ^c^Mean ± standard deviation (SD) of the geometric mean of the EGFP fluorescence intensity (FI) 3 days after electroporation. ^d^Mean ± SD of the median of the EGFP FI 3 days after electroporation. ^e^Mean ± SD of G418-resistant colonies per dish in which ~1 × 10^6^ electroporated DT40 cells were plated (see Materials and Methods). ^f^Plating efficiency was calculated as the percentage of the number of colonies formed in soft agarose medium containing no antibiotic relative to the 200 DT40 cells plated (see Materials and Methods). Mean ± SD of the plating efficiency is shown. ^g^The percentage of ZfL2-2 retrotransposition was determined with respect to the retrotransposition frequency of wild-type ZfL2-2 in wild-type DT40 in which control expression vector was transfected. pAneo; control expression vector. pKu70; chicken Ku70 expression vector. ^h^This value is overestimated because two of six independent experiments, in which no G418 colonies were produced, were assumed to generate one G418 colony per plate to calculate the retrotransposition frequency.
