# Supplementary material for: Genetic Evidence That the Non-Homologous End-Joining Repair Pathway Is Involved in LINE Retrotransposition
Source: PLoS Genet. 2009 Apr 24;5(4):e1000461. doi: 10.1371/journal.pgen.1000461 (PMC2666801; doi:10.1371/journal.pgen.1000461)
Supplement: Table S4 — The 102 ZfL2-2 insertions in chicken DT40 cells. (0.18 MB DOC) [file pgen.1000461.s019.doc]

Table S4: The 102 ZfL2-2 insertions in chicken DT40 cells

| ID | Cells | Clones | LR  (bp) | LI  (bp) | 5MH  (bp) | 5Ext  (bp) | 3MH  (bp) | 3Ext  (bp) | TSA  (bp) | Chr. | Direction | Position |
| --- | --- | --- | --- | --- | --- | --- | --- | --- | --- | --- | --- | --- |
| WT01 | WT | pWAF111.1 | 28 | 2562 | 0 | 0 | 4 | 0 | ND | 13 | + | ND |
| WT02 | WT | pWAF14.1 | 20 | 3082 | 0 | 13 | 3 | 0 | 9 | 1 | + | 111589432 |
| WT03 | WT | pWAF15.1 | 23 | 4776 | 3 | 0 | 3 | 0 | 6 | 20 | + | 12563139 |
| WT04 | WT | pWAF213.1 | 28 | 3873 | 3 | 0 | 4 | 0 | –1 | 2 | – | 147110473 |
| WT05 | WT | pWAF321.2 | 23 | 2509 | 0 | 0 | 2 | 0 | 6 | 1 | + | 156612318 |
| WT06 | WT | pWAZ432.1 | 22 | 2722 | 0 | 2 | 3 | 0 | 5 | 2 | – | 46298026 |
| WT07 | WT | pWA1.12a | 23 | 2489 | 1 | 0 | 5 | 0 | –4 | 3 | + | 82707451 |
| WT08 | WT | pWA1.17a | 24 | 3745 | 0 | 8 | 2 | 0 | 6 | 20 | + | 9981986 |
| WT09 | WT | pWA1.2a | 24 | 2402 | 5 | 0 | 2 | 0 | –6 | 1 | – | 54353549 |
| WT10 | WT | pWA1.4a | 26 | 2417 | 1 | 0 | 3 | 0 | 6 | 10 | + | 5514506 |
| WT11 | WT | pWA2.12a | 31 | 2326 | 2 | 0 | 2 | 0 | 1228 | 12 | – | 20026383 |
| WT12 | WT | pWA2.4a | 21 | 2406 | 2 | 0 | 3 | 0 | –7 | 10 | – | 9417576 |
| WT13 | WT | pWA3.6a | 22 | 2605 | 0 | 6 | 1 | 0 | 7 | 12 | – | 4387816 |
| WT14 | WT | pWA1.1a | 17 | 2403 | 2 | 0 | 0 | 1 | 7 | 12 | – | 19485653 |
| WT15 | WT | pWA9.4b | 22 | 2424 | 0 | 4 | 2 | 0 | 6 | 20 | – | 840019 |
| WT16 | WT | pWA11.5a | 22 | 3981 | 0 | 8 | 3 | 0 | 5 | 1 | + | 25514868 |
| WT17 | WT | pWA8.6a | 25 | 2580 | 0 | 52 | 3 | 0 | 8 | 1 | + | 158985681 |
| WT18 | WT | pWA9.1a | 26 | 3021 | 2 | 0 | 2 | 0 | 8 | 7 | – | 35531276 |
| WT19 | WT | pWA27.4a | 24 | 2548 | 0 | 43 | 2 | 0 | 5 | 3 | – | 88784596 |
| WT20 | WT | pWA27.6a | 22 | 5123 | 2 | 0 | 2 | 0 | –17 | 1 | – | 111882782 |
| WT21 | WT | pWA31.1a | 27 | 3022 | 0 | 26 | 3 | 0 | 5 | 3 | + | 52120160 |
| WT22 | WT | pWA33.2a | 21 | 2407 | 0 | 12 | 2 | 0 | 5 | 8 | + | 8872671 |
| WT23 | WT | pWA35.2a | 23 | 4368 | 2 | 0 | 3 | 0 | 4 | 9 | – | 7300048 |
| WT24 | WT | pWA37.3a | 23 | 2375 | 4 | 0 | 5 | 0 | 6 | 2 | + | 137136891 |
| WT25 | WT | pWA33.4a | 26 | 2413 | 0 | 76 | 4 | 0 | 7 | 1 | + | 98763222 |
| WT26 | WT | pWA36.2a | 22 | 3145 | 0 | 2 | 2 | 0 | 5 | 20 | – | 7515711 |
| KU01 | Ku70^–/–^ | p70A2.1a | 27 | 2423 | 3 | 0 | 5 | 0 | 8 | 10 | + | 5458429 |
| KU02 | Ku70^–/–^ | p70A3.1a | 24 | 4365 | 0 | 3 | 6 | 0 | 3 | 20 | – | 4415310 |
| KU03 | Ku70^–/–^ | p70A4.2a | 26 | 2911 | 0 | 5 | 7 | 0 | –451 | 2 | – | 116118899 |
| KU04 | Ku70^–/–^ | p70A6.1a | 27 | 3809 | 2 | 0 | 6 | 0 | –1564 | 1 | – | 17709247 |
| KU05 | Ku70^–/–^ | p70A6.3a | 25 | 5505^a^ | 2 | 0 | 3 | 0 | –3 | 12 | – | 2246920 |
| KU06 | Ku70^–/–^ | p70A7.2a | 26 | 5504^a^ | 0 | 2 | 3 | 0 | 6 | 2 | – | 30802576 |
| KU07 | Ku70^–/–^ | p70A9.3a | 23 | 4033 | 2 | 0 | 4 | 0 | 6 | 2 | – | 74217320 |
| KU08 | Ku70^–/–^ | p70A9.2b | 24 | 4577 | 0 | 15 | 1 | 0 | 3 | 3 | – | 40848568 |
| KU09 | Ku70^–/–^ | p70A11.1a | 25 | 2571 | 0 | 8 | 3 | 0 | 7 | 2 | – | 63142567 |
| KU10 | Ku70^–/–^ | p70A12.2a | 27 | 2419 | 0 | 0 | 3 | 0 | 0 | 1 | + | 67585231 |
| KU11 | Ku70^–/–^ | p70A14.1a | 24 | 5503^a^ | 1 | 0 | 2 | 0 | –7 | 3 | – | 112317305 |
| KU12 | Ku70^–/–^ | p70A18.1a | 16 | 2423 | 1 | 0 | 4 | 0 | 1 | 2 | – | 78441858 |
| KU13 | Ku70^–/–^ | p70A14.2a | 27 | 3968 | 0 | 24 | 3 | 0 | –343 | ND | ND | ND |
| KU14 | Ku70^–/–^ | p70A14.3a | 21 | 5504^a^ | 3 | 0 | 2 | 0 | 1 | ND | ND | ND |
| KU15 | Ku70^–/–^ | p70A15.1a | 23 | 2716 | 0 | 9 | 3 | 0 | 6 | 24 | + | 1383822 |
| KU16 | Ku70^–/–^ | p70A27.2a | 21 | 5503^a^ | 2 | 0 | 2 | 0 | –4 | 3 | – | 109987863 |
| KU17 | Ku70^–/–^ | p70A29.1a | 18 | 2425 | 1 | 0 | 1 | 0 | 4 | 4 | – | 90598724 |
| KU18 | Ku70^–/–^ | p70A29.2a | 21 | 2468 | 0 | 9 | 3 | 0 | –1019 | 1 | + | 33998635 |
| KU19 | Ku70^–/–^ | p70A30.1a | 26 | 2571 | 0 | 1 | 2 | 0 | 6 | 3 | – | 67015324 |
| KU20 | Ku70^–/–^ | p70A32.1a | 25 | 3041 | 1 | 0 | 2 | 0 | 3 | 3 | + | 33721749 |
| KU21 | Ku70^–/–^ | p70A32.2a | 23 | 3663 | 0 | 10 | 2 | 0 | 5 | 1 | – | 74886379 |
| KU22 | Ku70^–/–^ | p70A34.1a | 22 | 4503 | 0 | 26 | 4 | 0 | 6 | 2 | – | 36582092 |
| KU23 | Ku70^–/–^ | p70A36.1a | 27 | 2537 | 0 | 1 | 6 | 0 | –50187 | 2 | – | 34123694 |
| KU24 | Ku70^–/–^ | p70A35.1a | 19 | 5504^a^ | 0 | 8 | 2 | 0 | 10 | 27 | – | 4252949 |
| KU25 | Ku70^–/–^ | p70A37.3a | 26 | 5406 | 0 | 0 | 5 | 0 | 5 | 3 | + | 81429801 |
| AR01 | Artemis^–/–^ | pAAZ_A33.1 | 28 | 3436 | 2 | 0 | 7 | 0 | 8 | 1 | – | 154877403 |
| AR02 | Artemis^–/–^ | pAAZ_B13.1 | 21 | 3623 | 4 | 0 | 2 | 0 | ND | 5 | – | ND |
| AR03 | Artemis^–/–^ | pAAZ_B22.1 | 25 | 2660 | 0 | 1 | 3 | 0 | 2 | 2 | – | 15688452 |
| AR04 | Artemis^–/–^ | pAAZ_B31.1 | 25 | 3114 | 1 | 0 | 3 | 0 | 6 | 21 | + | 3230108 |
| AR05 | Artemis^–/–^ | pAA1.2a | 26 | 3818 | 0 | 2 | 6 | 0 | 8 | 3 | + | 57370503 |
| AR06 | Artemis^–/–^ | pAA6.1a | 22 | 2551 | 0 | 5 | 2 | 0 | 2 | 15 | + | 2256600 |
| AR07 | Artemis^–/–^ | pAA2.2a | 20 | 5504^a^ | 0 | 4 | 2 | 0 | 8 | 24 | + | 2537625 |
| AR08 | Artemis^–/–^ | pAA1.4a | 21 | 2390 | 0 | 8 | 3 | 0 | 8 | 8 | + | 18410470 |
| AR09 | Artemis^–/–^ | pAA5.1a | 24 | 2423 | 1 | 0 | 3 | 0 | 9 | 3 | + | 72442214 |
| AR10 | Artemis^–/–^ | pAA3.1a | 23 | 5504^a^ | 3 | 0 | 3 | 0 | 1 | 3 | – | 21226673 |
| AR11 | Artemis^–/–^ | pAA3.5a | 26 | 2906 | 0 | 6 | 4 | 0 | 7 | 2 | + | 5507894 |
| AR12 | Artemis^–/–^ | pAA11.3a | 23 | 2908 | 6 | 0 | 1 | 0 | 5 | 4 | – | 65439256 |
| AR13 | Artemis^–/–^ | pAA11.4a | 29 | 5503^a^ | 0 | 57 | 5 | 0 | 6 | 1 | + | 29276649 |
| AR14 | Artemis^–/–^ | pAA3.2a | 23 | 4830 | 2 | 0 | 4 | 0 | 3 | 8 | – | 13319814 |
| AR15 | Artemis^–/–^ | pAA9.3a | 24 | 2390 | 0 | 9 | 2 | 0 | 8 | 4 | – | 85319118 |
| AR16 | Artemis^–/–^ | pAA19.1a | 23 | 4397 | 1 | 0 | 4 | 0 | –9 | 2 | – | 147161354 |
| AR17 | Artemis^–/–^ | pAA17.4a | 28 | 4409 | 1 | 0 | 3 | 0 | 4 | ND | ND | ND |
| AR18 | Artemis^–/–^ | pAA16.1a | 20 | 5502^a^ | 0 | 5 | 2 | 0 | 5 | 24 | – | 6333918 |
| AR19 | Artemis^–/–^ | pAA16.3a | 26 | 3284 | 0 | 30 | 3 | 0 | 3 | Z | + | 42068986 |
| AR20 | Artemis^–/–^ | pAA17.2a | 27 | 3769 | 3 | 0 | 3 | 0 | 0 | 4 | – | 8836467 |
| AR21 | Artemis^–/–^ | pAA17.3a | 23 | 3420 | 0 | 15 | 2 | 0 | 8 | 5 | + | 48538776 |
| AR22 | Artemis^–/–^ | pAA18.3a | 20 | 3362 | 0 | 6 | 2 | 0 | 1 | 4 | – | 7011000 |
| AR23 | Artemis^–/–^ | pAA20.2a | 23 | 2457 | 0 | 3 | 3 | 0 | 5 | 4 | – | 91454571 |
| AR24 | Artemis^–/–^ | pAA19.4a | 25 | 2376 | 0 | 31 | 3 | 0 | 8 | 15 | – | 12371520 |
| LI01 | LigIV^–/–^ | p4AZ11.2 | 24 | 2515 | 3 | 0 | 2 | 0 | 2 | 20 | – | 796773 |
| LI02 | LigIV^–/–^ | p4AZ22.2 | 24 | 3078 | 0 | 16 | 1 | 0 | 5 | 2 | + | 55625437 |
| LI03 | LigIV^–/–^ | p4AZ31.2 | 24 | 2327 | 4 | 0 | 2 | 0 | 7 | 5 | – | 26704741 |
| LI04 | LigIV^–/–^ | p4AZA21.2 | 23 | 4517 | 0 | 1 | 2 | 0 | 6 | 2 | – | 110939185 |
| LI05 | LigIV^–/–^ | p4AZB21.1 | 19 | 2897 | 0 | 0 | 3 | 0 | 8 | 26 | – | 1055916 |
| LI06 | LigIV^–/–^ | p4AZD21.2 | 23 | 2990 | 0 | 6 | 2 | 0 | 5 | 3 | + | 13966507 |
| LI07 | LigIV^–/–^ | p4A4.3a | 23 | 5103 | 1 | 0 | 1 | 0 | 4 | 8 | – | 139372 |
| LI08 | LigIV^–/–^ | p4A1.8a | 20 | 4910 | 0 | 2 | 2 | 0 | 3 | 3 | – | 51837712 |
| LI09 | LigIV^–/–^ | p4A3.1a | 20 | 2527 | 0 | 5 | 1 | 0 | 3 | Z | + | 12846299 |
| LI10 | LigIV^–/–^ | p4A4.6a | 23 | 5295 | 2 | 0 | 2 | 0 | 3 | 3 | + | 89413413 |
| LI11 | LigIV^–/–^ | p4A1.1a | 23 | 3385 | 0 | 25 | 4 | 0 | 5 | 1 | + | 149757640 |
| LI12 | LigIV^–/–^ | p4A1.2a | 24 | 2809 | 0 | 5 | 6 | 0 | 5 | 1 | + | 147924185 |
| LI13 | LigIV^–/–^ | p4A1.4a | 24 | 5504^a^ | 0 | 15 | 2 | 0 | 7 | 12 | – | 15741236 |
| LI14 | LigIV^–/–^ | p4A1.5a | 16 | 3452 | 0 | 24 | 4 | 0 | 6 | 9 | – | 24275297 |
| LI15 | LigIV^–/–^ | p4A2.3a | 24 | 3400 | 0 | 10 | 2 | 0 | 7 | 7 | + | 35056502 |
| LI16 | LigIV^–/–^ | p4A3.3a | 23 | 2747 | 0 | 0 | 4 | 0 | 5 | Z | + | 45648449 |
| LI17 | LigIV^–/–^ | p4A2.6a | 23 | 2332 | 3 | 0 | 3 | 0 | 5 | 1 | + | 36888674 |
| LI18 | LigIV^–/–^ | p4A3.2a | 24 | 2424 | 0 | 3 | 2 | 0 | 3 | 3 | + | 30099962 |
| LI19 | LigIV^–/–^ | p4A13.3a | 17 | 2425 | 1 | 0 | 2 | 0 | 7 | 1 | + | 17302439 |
| LI20 | LigIV^–/–^ | p4A16.1a | 25 | 2425 | 4 | 0 | 2 | 0 | 3 | 1 | – | 135521653 |
| LI21 | LigIV^–/–^ | p4A16.2a | 26 | 2613 | 1 | 0 | 2 | 0 | 6 | 24 | + | 4216271 |
| LI22 | LigIV^–/–^ | p4A16.3a | 31 | 4175 | 0 | 4 | 4 | 0 | 9 | 2 | – | 51291146 |
| LI23 | LigIV^–/–^ | p4A16.4a | 19 | 3058 | 3 | 0 | 2 | 0 | 1 | Z | – | 21639243 |
| LI24 | LigIV^–/–^ | p4A15.6a | 22 | 5502^a^ | 2 | 0 | 3 | 0 | 5 | 2 | + | 27766470 |
| LI25 | LigIV^–/–^ | p4A17.3a | 26 | 2789 | 0 | 0 | 4 | 0 | 5 | 13 | + | 14718430 |
| LI26 | LigIV^–/–^ | p4A19.3a | 21 | 2456 | 0 | 6 | 3 | 0 | 6 | 9 | – | 23552583 |
| LI27 | LigIV^–/–^ | p4A20.1a | 24 | 2413 | 0 | 4 | 2 | 0 | –1266 | 12 | – | 18444020 |

LR, the length of the 3' terminal repeat (TGTAAA). LI, the length of the insertion. 5MH, the length of the 5' microhomology. 5Ext, the length of the 5' extra nucleotides. 3MH, the length of the 3' microhomology. 3Ext, the length of the 3' extra nucleotides. TSA, target site alterations; positive numbers indicate the length of target site duplication, zero indicates blunt end joining, negative numbers indicate the length of target site truncation. Chr., the chromosome in which each insertion occurred. Direction, the direction of each insertion. Position, the nucleotide position of each insertion in the chromosome. ND, could not be determined. ^a^Full-length insertions.
