# Supplementary material for: Genetic Evidence That the Non-Homologous End-Joining Repair Pathway Is Involved in LINE Retrotransposition
Source: PLoS Genet. 2009 Apr 24;5(4):e1000461. doi: 10.1371/journal.pgen.1000461 (PMC2666801; doi:10.1371/journal.pgen.1000461)
Supplement: Table S5 — Summary of ZfL2-2 insertions in chicken DT40 cells. (0.05 MB DOC) [file pgen.1000461.s020.doc]

Table S5: Summary of ZfL2-2 insertions in chicken DT40 cells

|  |  | **WT** | **Ku^–/–^** | **Art^–/–^** | **LigIV^–/–^** |
| --- | --- | --- | --- | --- | --- |
|  | Total number analyzed | 26 | 25 | 24 | 27 |
| **5' junction** | Microhomology | 12 (46%) | 10 (40%) | 10 (42%) | 10 (37%) |
|  | direct joining | 2 (8%) | 2 (8%) | 0 (0%) | 3 (11%) |
|  | extra nucleotides | 12 (46%) | 13 (52%) | 14 (58%) | 14 (52%) |
| **3' junction** | Microhomology | 25 (96%) | 25 (100%) | 24 (100%) | 27 (100%) |
|  | direct joining | 0 (0%) | 0 (0%) | 0 (0%) | 0 (0%) |
|  | extra nucleotides | 1 (4%) | 0 (0%) | 0 (0%) | 0 (0%) |
| **Insertion type** | full-length^a^ | 0 (0%) | 6 (24%) | 4 (17%) | 2 (7%) |
|  | 5' truncated | 26 (100%) | 19 (76%) | 20 (83%) | 25 (93%) |
|  | Total number analyzed | 25 | 25 | 23 | 27 |
| **Target site** | duplication (long, 1228 bp) | 1 (4%) | 0 (0%) | 0 (0%) | 0 (0%) |
|  | duplication (short, ≤20 bp) | 19 (76%) | 16 (64%) | 21 (91%) | 26 (96%) |
|  | blunt end joining | 0 (0%) | 1 (4%) | 1 (4%) | 0 (0%) |
|  | truncation (short, ≤20 bp) | 5 (20%) | 3 (12%) | 1 (4%) | 0 (0%) |
|  | truncation (long, 343–50187 bp) | 0 (0%) | 5 (20%) | 0 (0%) | 1 (4%) |

^a^ Insertions having a 5' end start site within the first five bases of the transcription start site are categorized as full-length.
