# Supplementary material for: Genetic Evidence That the Non-Homologous End-Joining Repair Pathway Is Involved in LINE Retrotransposition
Source: PLoS Genet. 2009 Apr 24;5(4):e1000461. doi: 10.1371/journal.pgen.1000461 (PMC2666801; doi:10.1371/journal.pgen.1000461)
Supplement: Table S6 — ZfL2-2 retrotransposition assay in HeLa cells with NU7026. (0.04 MB DOC) [file pgen.1000461.s021.doc]

Table S6: ZfL2-2 retrotransposition assay in HeLa cells with NU7026

| NU7026 | n^a^ | Number of G418^R^ colonies per dish^b^ | Plating efficiency^c^ (%) | Retrotransposition frequency  (mean ± SD) | Relative retrotransposition frequency ^d^ |
| --- | --- | --- | --- | --- | --- |
| 0 μM | 3 | 611 ± 148 | 6 ± 2 | 0.11 ± 0.02 | 100% |
| 5 μM | 3 | 277 ± 97 | 5 ± 2 | 0.05 ± 0.004 | 54% |
| 10 μM | 3 | 96 ± 43 | 3 ± 2 | 0.03 ± 0.01 | 28% |

^a^n indicates the number of independent experiments. ^b^Mean ± standard deviation of G418-resistant colonies per dish in which ~1 × 10^5^ electroporated HeLa cells were plated (see Materials and Methods). ^c^The plating efficiency was calculated as the percentage of the number of colonies formed in a 100-mm dish with no antibiotic relative to 1 × 10^4^ plated HeLa cells with Hyg^R^ (see Materials and Methods). ^d^The relative retrotransposition frequency of ZfL2-2 was calculated as a percentage of the retrotransposition frequency of ZfL2-2 without NU7026.
