# Supplementary material for: Genetic Evidence That the Non-Homologous End-Joining Repair Pathway Is Involved in LINE Retrotransposition
Source: PLoS Genet. 2009 Apr 24;5(4):e1000461. doi: 10.1371/journal.pgen.1000461 (PMC2666801; doi:10.1371/journal.pgen.1000461)
Supplement: Table S7 — L1 retrotransposition assay in HeLa cells with NU7026. (0.04 MB DOC) [file pgen.1000461.s022.doc]

Table S7: L1 retrotransposition assay in HeLa cells with NU7026

| NU7026 | n^a^ | Number of G418^R^ colonies per dish^b^ | Plating efficiency^c^ (%) | Retrotransposition frequency  (mean ± SD) | Relative retrotransposition frequency ^d^ |
| --- | --- | --- | --- | --- | --- |
| 0 μM | 3 | 630 ± 127 | 32 ± 5 | 0.39 ± 0.03 | 100% |
| 5 μM | 3 | 439 ± 106 | 28 ± 5 | 0.32 ± 0.02 | 80% |
| 10 μM | 3 | 269 ± 46 | 20 ± 2 | 0.27 ± 0.02 | 67% |

^a^n indicates the number of independent experiments. ^b^Mean ± standard deviation of G418-resistant colonies per dish in which ~5 × 10^3^ electroporated HeLa cells were plated (see Materials and Methods). ^c^The plating efficiency was calculated as the percentage of the number of colonies formed in a 100-mm dish with no antibiotic relative to 2 × 10^3^ plated HeLa cells with Hyg^R^ (see Materials and Methods). ^d^The relative retrotransposition frequency of L1 was calculated as a percentage of the retrotransposition frequency of L1 without NU7026.
